# The use of ketamine as a neuroprotective agent following cardiac arrest: A scoping review of current literature

**DOI:** 10.1111/cns.13983

**Published:** 2022-10-02

**Authors:** Marlena Ornowska, Andrew Wormsbecker, Gary Andolfatto, Tim S. Leung, Idan Khan, George Medvedev

**Affiliations:** ^1^ Department of Biomedical Physiology Simon Fraser University Burnaby British Columbia Canada; ^2^ Royal Columbian Hospital, Fraser Health Authority New Westminster British Columbia Canada; ^3^ Division of Critical Care, Department of Medicine University of British Columbia Vancouver British Columbia Canada; ^4^ Department of Emergency Medicine, Department of Medicine University of British Columbia Vancouver British Columbia Canada; ^5^ Faculty of Pharmaceutical Sciences University of British Columbia Vancouver British Columbia Canada; ^6^ Lower Mainland Pharmacy Services Vancouver British Columbia Canada; ^7^ Division of Neurology, Department of Medicine University of British Columbia Vancouver British Columbia Canada

**Keywords:** cardiopulmonary resuscitation, heart arrest, ketamine, neuroprotection, neuroprotective agents

## Abstract

**Aims:**

The objective of this article is to summarize the state of the literature surrounding the use of ketamine as a neuroprotective agent following cardiac arrest.

**Methods:**

Five electronic databases were used to search for studies related to the use of ketamine for neuroprotection following cardiac arrest. This search was performed once in May 2020, and an updated search was conducted in May 2021 and March 2022.

**Results:**

All searches combined retrieved 181 results; no clinical trials were identified. As such, the authors were limited to writing a scoping review of the literature rather than a systematic review.

**Conclusions:**

The current state of the literature describes the mechanism of action of ketamine as a neuroprotective agent through its action as an NMDA antagonist. There is evidence of its efficacy as a neuroprotective agent in preclinical models of cardiac arrest. Current published clinical work supports the use of ketamine ameliorating neurologic outcomes in other conditions such as epilepsy, traumatic brain injury, and depression. The current state of the literature is reflective of the notion that the use of ketamine following cardiac arrest may result in improved neurologic outcomes. Future research directions should focus on the use of ketamine as a possible clinical intervention following cardiac arrest.

## Graphical abstract

Ketamine holds promise as a neuroprotective agent through attenuating damage caused by the excitotoxic cascade. Though extensively studied in a stroke model, application to post‐cardiac arrest brain injury has been overlooked. This scoping review summarizes the mechanism of ketamine neuroprotection as applicable to post‐cardiac arrest brain injury, and existing evidence for the benefits of its use.

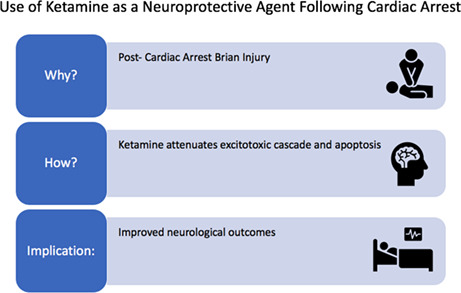

## 1 INTRODUCTION

Out‐of‐hospital cardiac arrest (CA) is defined as an abrupt loss of cardiac function and systemic circulation occurring outside of a hospital setting.^1^ CA patients experience poor neurologic outcomes; the majority of survivors remain afflicted by cognitive dysfunction secondary to CA.^2^ As a result, patients and their caregivers often need to make major life adjustments to cope with changes in brain function following CA. Those left with significant neurological deficits may become reliant on the healthcare system for additional support for years following their CA. This contributes to the costs of rehabilitation, neurologic, and psychological services.^3^ The development of post‐cardiac arrest brain injury (PCABI) is a significant determinant of mortality and neurologic outcome following CA.^4^ PCABI is the primary cause of death in 68% of out‐of‐hospital CA patients, regardless of the etiology of the arrest.^5^ In an effort to improve neurological outcomes, current CA guidelines include target temperature management (TTM) after the return of spontaneous circulation (ROSC). This is defined as cooling a comatose patient to a temperature of 33°C for at least 28 h, followed by gradual rewarming to 37°C.^6^ Initially called therapeutic hypothermia, the positive effect of cooler temperatures on neurologic outcomes in the setting of CA was established in 2002.^7^, ^8^ This notion was challenged in 2013 when a large multicenter trial showed non‐inferiority in mortality and neurologic functional outcomes in patients randomized to the controlled hypothermia (at 32–34°C) condition compared to controlled normothermia condition.^9^ This result was repeated in 2021, when Dankiewicz et al.^6^ showed no significant difference in death and functional outcome at 6 months in patients randomized to hypothermia versus normothermia groups. Experts suggest this may be the avoidance of hyperthermia, rather than induction of hypothermia, which is beneficial to the patient.^6^, ^10^, ^11^ Although TTM is a relatively effective tool, patient's neurological outcomes may be further improved by introducing neuroprotective agents such as ketamine during post‐CA care.^12^, ^13^, ^14^ In the emergency clinical setting, ketamine is one option that may be used for sedation of CA patients during routine resuscitation procedures. The purpose of this scoping review is to explore the utility of ketamine in post‐CA management to improve neurological outcomes of CA survivors.

## 2 METHODS

The search strategy included searching five electronic databases (Medline, CINAHL, EMBASE, Cochrane Database of Systematic Reviews, Cochrane Central Register of Controlled Trial) using “Ketamine”, “Neuroprotective Agents”, “Neuroprotection” and “Heart Arrest” as search terms, including MeSH headings. The first search was completed by a health sciences research librarian on May 28, 2020. The search was then updated, and repeated on May 28, 2021, and March 4, 2022, each time with dates limited to the time between searches. Results of each search are available as Appendices [Link], [Link]. All papers identified by both searches were reviewed by the study authors. All work related to this review was performed at the Royal Research Library. The study methodology did not require ethical approval. This study was unfunded.

## 3 RESULTS

The results of the first search revealed 37 papers. A total of 73 articles were retrieved based on the results of the second search and 71 during the last search. All three sets of results revealed no clinical trials that have directly investigated the application of ketamine as sedation to improve neurological outcomes. As such, it was not possible for the authors to conduct a systematic review. Instead, detailed literature and scoping review based on the results of our search were undertaken to summarize the evidence supporting the use of ketamine as a neuroprotective agent in the context of CA. Results of all searches are available in Appendices [Link], [Link].

## 4 DISCUSSION

As the cerebrum requires 15%–20% of cardiac output to maintain function,^15^ any loss of blood flow may result in ischemic damage. Sekhon and colleagues define this primary ischemia as the first hit in the “two‐hit” model of PCABI.^4^, ^16^ A major cellular mechanism contributing to primary injury is excitotoxicity. The excitotoxic cascade is initiated within minutes of ischemia onset, triggered by the dysfunction of the sodium‐potassium pump of neuronal cell membranes.^4^, ^17^ As the sodium‐potassium pump is coupled with a calcium exchanger, failure of the pump to maintain physiological gradients of sodium and potassium initiates a cellular cascade that promotes an increase in intracellular calcium, causing uncontrolled fusion of glutamate‐containing vesicles with the presynaptic membrane.^18^, ^19^

Upon resuscitation of the patient and subsequent ROSC, oxygen is returned to injured tissues that have already adapted to consumption at diminished rates. This imbalance of oxygen delivery and utilization gives rise to a secondary form of damage known as the “second hit” or “reperfusion injury” of PCABI.^4^, ^16^ Increase in intracellular calcium levels during the primary ischemic event results in glutamate exocytosis onto the post‐synaptic cell and subsequent upregulated expression of calcium‐permeable NMDA receptors. This causes a further increase of intracellular calcium in the postsynaptic cell.^19^ The elevated intracellular calcium in both pre‐ and post‐synaptic neurons leads to increased generation of reactive oxygen species (ROS), activation of proteases and phospholipases,^16^ and initiation of apoptotic cascades.^20^, ^21^ There is also evidence of an innate immune activation during reperfusion injury, resulting in increased permeability of the blood–brain barrier and vasogenic edema leading to intracranial hypotension.^16^, ^22^

Other pathophysiological conditions resulting from secondary injury include anemia, impaired autoregulation, hypo‐ or hypercapnia‐associated vascular response, hyperoxia leading to increased free radicals, and hyperthermia.^4^, ^16^, ^23^ Reperfusion injury may begin to form immediately after ROSC and continue for up to 72 h following CA, into the intermediate phase of post‐CA syndrome.^24^, ^25^ When combined with other mechanisms of secondary injury following PCABI, excitotoxicity serves to exacerbate inflammation and propagate free‐radical damage.^4^

PCABI results in apoptosis of neurons, particularly in the highly metabolically active hippocampal and neocortical areas. Clinical manifestations of this damage include impairments in memory, executive functioning, and visual‐motor skills.^2^ Additionally, PCABI may result in psychiatric comorbidities including depression, anxiety, and post‐traumatic stress disorder. This may significantly impact the quality of life of CA survivors.^26^ Despite having enormous consequence on post‐CA life of survivors, preventing PCABI is not a primary focus of current standard of care, which is mostly concerned with resuscitation of the heart and restoration of spontaneous circulation. Additional efforts could be added to post‐CA standard of care with an additional focus on neuroprotection.

One opportunity for clinical intervention to attenuate damage caused by excitotoxicity is the administration of ketamine. The role of ketamine in inhibiting the excitotoxic process is two‐fold; in interfering with the ability of glutamate‐containing vesicles to bind to the presynaptic membrane (via reduction of presynaptic protein attachment receptors) and in non‐competitive antagonism of NMDA receptors, specifically those with the GluN2B subunit.^20^ By preventing glutamate exocytosis and blocking calcium entry into the post‐synaptic cell, intracellular calcium buildup may be diminished. If ketamine is administered following CA upon hospital admission for sedation, it may be effective in inhibiting the above‐mentioned apoptotic cascades, thus resulting in improved neurologic outcomes.

Indeed, there is evidence in preclinical models of the protective effects of ketamine on neuronal apoptosis. A summary of these studies can be found in Table 1. Church et al.^12^ used a rat model of ischemia induced by occlusion of carotid arteries to investigate the effect of ketamine and another NDMA receptor agonist on levels of brain injury based on histologic scoring of the CA1 and CA3 regions of the hippocampus. This group explored several variations of ketamine administration, including the effects of 20 mg/kg of administered pre‐ischemia and a low versus high dose administered both pre‐ and post‐induction of ischemia (5 and 10 mg/kg, 10, and 20 mg/kg, respectively). Results were compared to sham‐operated and non‐treated ischemia controls. Findings suggested that ketamine offered significant protection against apoptosis, with greater neuronal preservation in the higher dose group.^12^ Similar results were also achieved in a study by Xu et al.,^27^ who used a zebrafish model to show that pretreatment with midazolam or ketamine significantly inhibited calcium wave propagation post‐CA as compared to non‐treated controls, as evidenced by confocal imaging techniques. Results of subsequent TUNEL staining confirmed significantly lower levels of neuronal apoptosis in the pre‐treated groups. No significant difference was detected between ketamine and midazolam conditions.^27^

**TABLE 1:** Main findings of reviewed articles investigating use of ketamine for neuroprotection in the setting of cardiac arrest, cardiac surgery, or ischemia

First author	Study type	Sample size (*n*) per group	Ketamine dose(s)	Timing	Results
Church et al.	Preclinical, rat model using complete forebrain ischemia	7	5, 10, and/or 20 mg/kg	Pre‐ischemia, post‐ischemia, or pre‐ and post‐ischemia	Significantly greater percentage of normal CA1 hippocampal pyramidal neuron counts in pre‐ and post‐ischemia treated groups compared to non‐treated ischemic controls
Xu et al.	Preclinical, zebrafish model using cardiac arrest	15	2.5 mM	Pre‐cardiac arrest	Significantly decreased calcium wave propagation and neuronal apoptosis, and increased 5‐day survival rate compared to untreated CA controls and midazolam pretreatment groups
Giuliano et al.	Preclinical, canine model using hypothermic circulatory arrest followed by cardiopulmonary bypass	5	1.425 mg/kg	Pre‐ and post‐cardiac arrest and bypass	Significantly lower neurobehavioral deficit scores 24 and 48 h post‐arrest, significantly lower phosphorylated neurofilament‐H and neuron‐specific enolase compared to saline treatment
Proescholdt et al.	Preclinical, rat model using global forebrain ischemia	6	30, 60, or 90 mg/kg S(+), 90 mg/kg R(−)	Post‐ischemia	Significantly greater cortical oxygen saturation in S(+) groups compared to saline treatment
Engelhard et al.	Preclinical, rat model using incomplete cerebral ischemia	8	1 mg/kg/min S(+)	Pre‐ischemia	Significantly lower expression of hippocampal proapoptotic (Bax) and trend toward increased expression of antiapoptotic proteins (Bcl‐2) compared to sham‐operated control
Hudetz et al.	Clinical, male cardiac surgery patients recruited from a single center	26	0.5 mg/kg	Pre‐surgery	Significantly lower CRP concentrations one day post‐op, and higher scores in non‐verbal memory, verbal memory, and executive function testing 1 week post‐op as compared to placebo group

A more recent preclinical study published by Giuliano et al.^28^ examined the effect of ketamine administered following cardiopulmonary bypass and subsequent circulatory arrest in a canine model. In this study, 1.425 mg/kg of ketamine was administered prior to the bypass procedure, and another 1.425 mg/kg following hypothermic circulatory arrest in the experimental group. Subjects who received ketamine exhibited significantly lower neurobehavioral deficit scores (as measured by the Pittsburgh Veterinary score) compared to untreated controls. Scores were evaluated at 24 and 48 h post‐ketamine administration. Scores were also lower at the 72‐h evaluation, but did not reach significance (30 vs. 89, *p* = 0.069). Markers of neuronal injury (neurofilament‐H and neuron‐specific enolase) were also significantly lower in subjects who received ketamine treatment.^28^

Investigations into relative neuroprotective effects of different isomeric forms of ketamine have also been completed. Proescholdt et al. investigated the effect of S(+) versus R(−) stereoisomers of ketamine on functional and structural outcomes following induction of global forebrain ischemia in a rat model.^13^ Results from microphotospectrometry and histology indicated improved cortical O_2_ saturation and reduced neuronal cell loss in rats treated with S(+) ketamine; this effect was more pronounced in animals randomized to the higher dose group (90 mg). Finally, a study conducted by Engelhard et al. showed that administration of 1 mg/kg/min of S(+)‐ketamine and O_2_ prior to incomplete cerebral ischemia in rats resulted in reduced expression of pro‐apoptotic proteins (Bax) and increased in anti‐apoptotic proteins (Bcl2), as evidenced by the results of Western Blot analysis.^14^

In addition to preclinical research, there is a growing body of evidence from clinical literature about the use of ketamine for neuroprotection following a variety of neurologic insults. For example, the use of ketamine in the setting of severe epilepsy resistant to anticonvulsants and benzodiazepines has been met with success.^29^ This is especially relevant to the CA population as post‐anoxic electrographic seizure activity is a common consequence of CA.^30^, ^31^ Similarly, recent reviews by Mihaljevic et al. and Pribish et al. outline its potential for use modulating glutamate levels in the treatment of depression.^32^, ^33^

Additionally, Hudetz et al. conducted a study investigating the effect of ketamine on postoperative cognitive dysfunction following cardiac surgery.^34^ During this trial, one group of patients was randomized to receive a 0.5‐mg/kg ketamine bolus during anesthetic induction; results of verbal and non‐verbal memory and executive functioning tests, as well as levels of C‐Reactive Protein (CRP), were compared to a control group receiving a saline bolus and to non‐surgical control. Results indicated a decrease in neurocognitive functioning compared to baseline in seven patients in the ketamine group versus 21 patients in the placebo group. In addition, levels of CRP were found to be lower across all subjects in the ketamine group compared to the control.^34^ Further information is summarized in Table 1.

Finally, the administration of ketamine has also been found to reduce spreading depolarizations accompanying acute brain injury in patients suffering from traumatic brain injury, subarachnoid hemorrhage, and malignant hemispheric stroke. These depolarizations are believed to lead to the breakdown of functional neurons and are associated with poor patient outcomes.^35^ One preclinical study demonstrated improved post‐TBI behavioral changes in mice administered ketamine, and ketamine combined with another sedative.^36^

The results of this scoping review demonstrate a clear link between the mechanism of PCABI and the ability of ketamine to inhibit its progression. Early work suggests potential for benefit of using ketamine as a sedative in the emergency post‐CA setting to improve the neurological outcome of CA patients. However, the application of ketamine for neuroprotection following CA has not been widely investigated in the clinical setting. The authors feel the lack of trials directly investigating this relationship may be caused by the tendency of researchers to use a stroke model to represent ischemic damage in both the preclinical and clinical settings. The lack of CA‐based neuroprotection trials results in a missed opportunity for the patient population that may benefit from such interventions the most. Similarly, the use of ketamine to improve neurological outcomes has been investigated in many different types of neurological conditions but has yet to be applied to CA patients. Together, the use of CA as an appropriate experimental model as well the application of neuroprotective effects of ketamine to this population have been overlooked in work done to date, setting the stage for an excellent future research opportunity.

Differences in the receptor‐antagonist mechanism of ketamine versus memantine have been the focus of many recent studies. It is hypothesized that memantine is more effective at inhibiting NMDA receptor subtypes containing the GluN2A subunit, in contrast to those with the 2B subunit targeted by ketamine.^20^, ^37^ Both GuN2A and GLuN2B subtypes are expressed in synaptic and extrasynaptic locations, with 2A predominating as the primary synaptic receptor in many types of neurons.^38^, ^39^ Excitotoxicity is modulated by both extrasynaptic and synaptic NMDA receptors, rendering memantine another promising choice for neuroprotection.^37^, ^40^, ^41^, ^42^, ^43^

Newer work by Glasgow et al.^37^ indicates that antagonistic activity of memantine on NMDA receptors may also be dependent upon intracellular calcium concentration, in addition to receptor subtype and location. Under heightened intracellular calcium concentration conditions, NMDA receptors have been shown to undergo calcium‐dependent desensitization via binding of calcium to the intracellular portion of the receptor after its entrance through the pore.^44^ As such, desensitization serves to limit further calcium entry under pathological conditions. Memantine has been shown to slow recovery from calcium‐dependent desensitization in the 2A NMDA receptor subtype.^37^ This mechanism may allow memantine to preferentially act on NMDA receptors in regions of excessive intracellular calcium accumulation, such as those explicitly affected by excitotoxicity following CA.^37^, ^44^ Future research should also focus on comparing the efficacy of ketamine to that of memantine in the context of neuroprotection in CA survivors, in addition to other NMDA receptor antagonists, such as dextromethorphan.^45^

Although a promising future research direction, the results of this scoping review should be interpreted primarily as hypothesis‐generating. Results of rigorous clinical studies are needed to provide a higher level of evidence for the use ketamine for neuroprotection. Other NMDA receptor antagonists, such as memantine, may also be considered.^46^

### 4.1 Other considerations

In the past, clinicians have been trained to consider neurological injury as a contraindication for the use of ketamine as a sedative.^47^, ^48^ This contraindication originated from the results of six studies published from 1971 to 1972 that reported an increase in intracranial pressure (ICP) after ketamine administration.^49^, ^50^, ^51^, ^52^, ^53^, ^54^ These studies have since been the subject of scrutiny in the academic community and have been criticized for being underpowered and confounded due to the inclusion of patients with abnormal cerebrospinal fluid pathways.^50^, ^51^, ^52^, ^53^, ^54^, ^55^ Additionally, many of these studies reported no change in ICP in healthy individuals and did not report concurrent changes in mean arterial pressure and calculated cerebral perfusion pressure.^48^ As such, current opinions consider the rise in ICP caused by ketamine to be a myth.^33^, ^56^, ^57^ In fact, in more recent preclinical trials modeling increases in ICP, racemic ketamine (10 mg/kg) was shown to decrease ICP by a maximum of 10.8%.^58^ Additionally, results of a systematic review by Cohen et al. showed that the use of ketamine in critically ill patients did not significantly affect cerebral perfusion pressure, neurologic outcomes, ICU length of stay, or mortality as compared to other sedatives including sufentanil, etomidate, remifentanil, and fentanyl.^59^ Current expert opinions state that ketamine should be considered for all patients with normal cerebrospinal fluid flow in a variety of situations, including procedural sedation, rapid sequence intubation, and analgesia.^55^

## 5 LIMITATIONS

Limitations of this article include the lack of systematic review procedures to appraise available evidence and meta‐analyze research results. This limitation could not be addressed due to the lack of clinical trials evaluating the topic of this literature review, in addition to the large heterogeneity of types of studies and reports retrieved by the results of the literature search. As such, the body of knowledge summarized in this article is intended to serve as a scoping review only and not a formal analysis of available evidence.

## 6 CONCLUSION

Ketamine has shown promise in attenuating neurologic injury caused by CA. The authors propose that the administration of ketamine following ROSC in CA patients may result in improved neurologic outcomes. However, evidence from the literature is indirect, based on in vitro or other neurological models, and thus should be interpreted cautiously. CA provides an excellent model to study the neuroprotective effects of ketamine in future research efforts.

## AUTHOR CONTRIBUTIONS

The manuscript complies with all instructions to the authors. All authors have met authorship requirements, and the final manuscript was approved by all authors. This manuscript has not been published elsewhere and is not under consideration by another journal. MO, AW, GA, TL, IK, and GM contributed to developing the concepts and layout of the article. GM was responsible for coming up with search terms and concept for the literature review. MO was responsible for conducting the literature search alongside research librarians, reviewing relevant sources, writing the manuscript, and referencing the works cited. AW, GA, TL, IK, and GM contributed to editing the article and adding relevant information about supporting studies or physiological concepts mentioned.

## CONFLICT OF INTEREST

No authors hold any conflicts of interest.

## Supporting information


Appendix S1

Appendix S2

Appendix S3

## Data Availability

The data that support the findings of this study are available from the corresponding author upon reasonable request.
