# Supplementary material for: The use of ketamine as a neuroprotective agent following cardiac arrest: A scoping review of current literature
Source: CNS Neurosci Ther. 2022 Oct 2;29(1):104–10. doi: 10.1111/cns.13983 (PMC9804040; doi:10.1111/cns.13983)
Supplement: Supplementary file 1 — Appendix S1 [file CNS-29-104-s002.pdf]

Literature Search Results

Date Completed: 2022-03-04
Requestor: Marlena Ornowska

Request: Update search from 2021-05-28 (request ID 9252)

1) information about cardiac arrest as a model used for neuro-protection studies. Most of the time in these types of studies, a stroke model is used. We are interested in finding out whether anyone has investigated usage of cardiac arrest as a model for neurological injury.

2) Neuronal injury following cardiac arrest and neuro-protection.

3) General literature search about ketamine in clinical practice in neuroprotection. Perhaps this search can include The following terms: ketamine, neuroprotection, experimental (any work)?

Completed By: Brooke Ballantyne Scott

Search Strategy:

Databases Searched:
Medline
EMBASE
CINAHL
Cochrane Database of Systematic Reviews
Cochrane Central Register of Controlled Trials

Terms searched:

MeSH:
Ketamine
Neuroprotective Agents
Neuroprotection
Heart Arrest

CINAHL:
Ketamine
Neuroprotective Agents
Heart Arrest

Embase SH:
ketamine
neuroprotection
heart arrest

Keywords:
neuron* n3 injur*
neuroprotect*

Results:

1. S., N., M.C., T.-C., B.J., R., S.C., T., W.M., van den B., N.A., F., … Hofmeijer J. (2022). **Effects of targeted temperature management at 33 degreeC vs. 36 degreeC on comatose patients after cardiac arrest stratified by the severity of encephalopathy**. *Resuscitation*. https://doi.org/https://dx.doi.org/10.1016/j.resuscitation.2022.01.026
Objectives: To assess neurological outcome after targeted temperature management (TTM) at 33 degreeC vs. 36 degreeC, stratified by the severity of encephalopathy based on EEG-patterns at 12 and 24 h. Design(s): Post hoc analysis of prospective cohort study. Setting(s): Five Dutch Intensive Care units. Patient(s): 479 adult comatose post-cardiac arrest patients. Intervention(s): TTM at 33 degreeC (n = 270) or 36 degreeC (n = 209) and continuous EEG monitoring. Measurements and Main Results: Outcome according to the cerebral performance category (CPC) score at 6

months post-cardiac arrest was similar after 33 degreeC and 36 degreeC. However, when stratified by the severity of encephalopathy based on EEG-patterns at 12 and 24 h after cardiac arrest, the proportion of good outcome (CPC 1-2) in patients with moderate encephalopathy was significantly larger after TTM at 33 degreeC (66% vs. 45%; Odds Ratios 2.38, 95% CI = 1.32-4.30; p = 0.004). In contrast, with mild encephalopathy, there was no statistically significant difference in the proportion of patients with good outcome between 33 degreeC and 36 degreeC (88% vs. 81%; OR 1.68, 95% CI = 0.65-4.38; p = 0.282). Ordinal regression analysis showed a shift towards higher CPC scores when treated with TTM 33 degreeC as compared with 36 degreeC in moderate encephalopathy (cOR 2.39; 95% CI = 1.40-4.08; p = 0.001), but not in mild encephalopathy (cOR 0.81 95% CI = 0.41-1.59; p = 0.537). Adjustment for initial cardiac rhythm and cause of arrest did not change this relationship. Conclusion(s): Effects of TTM probably depend on the severity of encephalopathy in comatose patients after cardiac arrest. These results support inclusion of predefined subgroup analyses based on EEG measures of the severity of encephalopathy in future clinical trials.Copyright © 2022 Elsevier B.V.

2. K., G., E., E., A.K., V., M.A., W., M.E., B., J.C., T., … Lawton J.S. AO - Giuliano, K. O. https://orcid. org/000.-0003-2260-1854. (2022). **Ketamine Mitigates Neurobehavioral Deficits in a Canine Model of Hypothermic Circulatory Arrest**. *Seminars in Thoracic and Cardiovascular Surgery*. https://doi.org/https://dx.doi.org/10.1053/j.semtcvs.2021.12.004
Hypothermic circulatory arrest is a protective technique used when complete cessation of circulation is required during cardiac surgery. Prior efforts to decrease neurologic injury with the NMDA receptor antagonist MK801 were limited by unacceptable side effects. We hypothesized that ketamine would provide neuroprotection without dose-limiting side effects. Canines were peripherally cannulated for cardiopulmonary bypass, cooled to 18degreeC, and underwent 90 minutes of circulatory arrest. Ketamine-treated canines (n = 5; total dose 2.85 mg/kg) were compared to untreated controls (n = 10). A validated neurobehavioral deficit score was obtained at 24, 48, and 72 hours (0 = no deficits/normal exam; higher score represents increasing deficits). Biomarkers of neuronal injury in the cerebrospinal fluid were examined at baseline and at 8, 24, 48, and 72 hours. Brain histopathologic injury was scored at 72 hours (higher score indicates more necrosis and apoptosis). Ketamine-treated canines had significantly improved, lower neurobehavioral deficit scores compared to controls (overall P = 0.003; 24 hours: median 72 vs 112, P = 0.030; 48 hours: 47 vs 90, P = 0.021; 72 hours: 30 vs 89, P = 0.069). Although the histopathologic injury scores of ketamine-treated canines (median 12) were lower than controls (16), there was no statistical difference (P = 0.10). Levels of phosphorylated neurofilament-H and neuron specific enolase, markers of neuronal injury, were significantly lower in ketamine-treated animals (P = 0.010 and = 0.039, respectively). Ketamine significantly reduced neurologic deficits and biomarkers of injury in canines after hypothermic circulatory arrest. Ketamine represents a safe and approved medication that may be useful as a pharmacologic neuroprotectant during cardiac surgery with circulatory arrest.Copyright © 2021 Elsevier Inc.

3. R., Z., T.D., B., G.M., F., J., L., S., R., J., M., … Sanderson T H. (2022). **Rapid Treatment with Intramuscular Magnesium Sulfate During Cardiopulmonary Resuscitation Does Not Provide Neuroprotection Following Cardiac Arrest**. *Molecular Neurobiology*. https://doi.org/https://dx.doi.org/10.1007/s12035-021-02645-x
Brain injury is the most common cause of death for patients resuscitated from cardiac arrest. Magnesium is an attractive neuroprotective compound which protects neurons from ischemic injury by reducing neuronal calcium overload via NMDA receptor modulation and preventing calcium-induced mitochondrial permeability transition. Intramuscular (IM) delivery of MgSO4 during CPR has the potential to target these mechanisms within an early therapeutic window. We hypothesize that IM MgSO4 administrated during CPR could achieve therapeutic serum magnesium levels within 15 min after ROSC and improve neurologic outcomes in a rat model of asphyxial cardiac arrest. Male Long Evans rats were subjected to 8-min asphyxial cardiac arrest and block randomized to receive placebo, 107 mg/kg, 215 mg/kg, or 430 mg/kg MgSO4 IM at the onset of CPR. Serum magnesium concentrations increased rapidly with IM delivery during CPR, achieving twofold to fourfold increase by 15 min after ROSC in all magnesium dose groups. Rats subjected to cardiac arrest or sham surgery were block randomized to treatment groups for assessment of neurological outcomes. We found that IM MgSO4 during CPR had no effect on ROSC rate (p > 0.05). IM MgSO4 treatment had no statistically significant effect on 10-day survival with good neurologic function or hippocampal CA1 pyramidal neuron survival compared to placebo treatment. In conclusion, a single dose IM MgSO4 during CPR achieves up to fourfold baseline serum magnesium levels within 15 min after ROSC; however, this treatment strategy did not improve survival, recovery of neurologic function, or neuron survival. Future studies with repeated dosing or in combination with hypothermic targeted temperature management may be indicated.Copyright © 2021, The Author(s), under exclusive licence to Springer Science+Business Media, LLC, part of Springer Nature.

4. Augoustides J G. (2022). **Commentary: Neuroprotection in Hypothermic Circulatory Arrest - is Ketamine a Magic Bullet?** *Seminars in Thoracic and Cardiovascular Surgery*. https://doi.org/https://dx.doi.org/10.1053/j.semtcvs.2021.12.008

5. Shoaib, M., & Becker, L. B. (2022). **A walk through the progression of resuscitation medicine.** *Annals of the New York Academy of Sciences*, *1507*(1), 23–36. https://doi.org/https://dx.doi.org/10.1111/nyas.14507
Cardiac arrest (CA) is a sudden and devastating disease process resulting in more deaths in the United States than many cancers, metabolic diseases, and even car accidents. Despite such a heavy mortality burden, effective treatments have

remained elusive. The past century has been productive in establishing the guidelines for resuscitation, known as cardiopulmonary resuscitation (CPR), as well as developing a scientific field whose aim is to elucidate the underlying mechanisms of CA and develop therapies to save lives. CPR has been successful in reinitiating the heart after arrest, enabling a survival rate of approximately 10% in out-of-hospital CA. Although current advanced resuscitation methods, including hypothermia and extracorporeal membrane oxygenation, have improved survival in some patients, they are unlikely to significantly improve the national survival rate any further without a paradigm shift. Such a change is possible with sustained efforts in the basic and clinical sciences of resuscitation and their implementation. This review seeks to discuss the current landscape in resuscitation medicine-how we got here and where we are going. Copyright © 2020 New York Academy of Sciences.

6. A., P., Perman S.M. AO - Presciutti Sarah M.; ORCID: https://orcid.org/0000-0002-0849-0847, A. O. https://orcid. org/000.-0002-5911-6142 A. O.-P., Presciutti, A., & Perman, S. M. (2022). **The evolution of hypothermia for neuroprotection after cardiac arrest: a history in the making**. *Annals of the New York Academy of Sciences*, *1507*(1), 60–69. https://doi.org/https://dx.doi.org/10.1111/nyas.14676
While much has been observed regarding hypothermia by way of environmental exposure, it is modern day medicine that deployed hypothermia as a therapeutic. From the early 1930s, when Temple Fay deployed "refrigeration" to treat pain, to the work of Wilfred Bigelow and Charles Drew, who utilized hypothermia in open heart surgery-the opportunities seemed endless. However, questions arose surrounding appropriate temperatures to achieve best outcomes and how to minimize adverse events, such as coagulopathy and infection. In the 1980s, hypothermia underwent a resurgence through Peter Safar's critical studies in large animals, which quickly translated into feasibility studies and the landmark trials of 2002 that paved the way for postcardiac arrest care as we currently know it. Through clinical and observational trials, modern-day targeted temperature management continues to adapt, striving to improve patient outcomes. While hypothermia has come a long way from the writings of Hippocrates, the ideal therapy has not yet been defined, and more work is needed. While the history is long, there is more to be written and advances to be achieved as we optimize the neuroprotective effects of hypothermia for comatose survivors of cardiac arrest. Copyright © 2021 New York Academy of Sciences.

7. Katz, A., Brosnahan, S. B., Papadopoulos, J., Parnia, S., Lam, J. Q., A., K., … Lam J.Q. AO - Brosnahan, S. B. . O. https://orcid. org/000.-0002-2092-3633. (2022). **Pharmacologic neuroprotection in ischemic brain injury after cardiac arrest.** *Annals of the New York Academy of Sciences*, *1507*(1), 49–59. https://doi.org/https://dx.doi.org/10.1111/nyas.14613
Cardiac arrest has many implications for morbidity and mortality. Few interventions have been shown to improve return of spontaneous circulation (ROSC) and long-term outcomes after cardiac arrest. Ischemic-reperfusion injury upon achieving ROSC creates an imbalance between oxygen supply and demand. Multiple events occur in the postcardiac arrest period, including excitotoxicity, mitochondrial dysfunction, and oxidative stress and inflammation, all of which contribute to ongoing brain injury and cellular death. Given that complex pathophysiology underlies global brain hypoxic ischemia, neuroprotective strategies targeting multiple stages of the neuropathologic cascade should be considered as a means of mitigating secondary neuronal injury and improving neurologic outcomes and survival in cardiac arrest victims. In this review article, we discuss a number of different pharmacologic agents that may have a potential role in targeting these injurious pathways following cardiac arrest. Pharmacologic therapies most relevant for discussion currently include memantine, perampanel, magnesium, propofol, thiamine, methylene blue, vitamin C, vitamin E, coenzyme Q10 , minocycline, steroids, and aspirin. Copyright © 2021 New York Academy of Sciences.

8. Wisloff-Aase, K., Skulstad, H., Beitnes, J. O., Lundblad, R., Halvorsen, P. S., Fiane, A., … Espinoza, A. (2022). **Left Ventricular Function Changes Induced by Moderate Hypothermia Are Rapidly Reversed After Rewarming-A Clinical Study.** *Critical Care Medicine*, *50*(1), e52–e60. https://doi.org/10.1097/CCM.0000000000005170
OBJECTIVES: Targeted temperature management (32-36degreeC) is used for neuroprotection in cardiac arrest survivors. The isolated effects of hypothermia on myocardial function, as used in clinical practice, remain unclear. Based on experimental results, we hypothesized that hypothermia would reversibly impair diastolic function with less tolerance to increased heart rate in patients with uninsulted hearts., DESIGN: Prospective clinical study, from June 2015 to May 2018., SETTING: Cardiothoracic surgery operation room, Oslo University Hospital., PATIENTS: Twenty patients with left ventricular ejection fraction greater than 55%, undergoing ascending aorta graft-replacement connected to cardiopulmonary bypass were included., INTERVENTIONS: Left ventricular function was assessed during reduced cardiopulmonary bypass support at 36degreeC, 32degreeC prior to graft-replacement, and at 36degreeC postsurgery. Electrocardiogram, hemodynamic, and echocardiographic recordings were made at spontaneous heart rate and 90 beats per minute at comparable loading conditions., MEASUREMENTS AND MAIN RESULTS: Hypothermia decreased spontaneous heart rate, and R-R interval was prolonged (862 +/- 170 to 1,156 +/- 254 ms, $p < 0.001$). Although systolic and diastolic fractions of R-R interval were preserved (0.43 +/- 0.07 and 0.57 +/- 0.07), isovolumic relaxation time increased and diastolic filling time was shortened. Filling pattern changed from early to late filling. Systolic function was preserved with unchanged myocardial strain and stroke volume index, but cardiac index was reduced with maintained mixed venous oxygen saturation. At increased heart rate, systolic fraction exceeded diastolic fraction (0.53 +/- 0.05 and 0.47 +/- 0.05) with diastolic impairment. Strain and stroke volume index were reduced, the latter to 65% of stroke volume index at spontaneous heart rate. Cardiac index decreased, but mixed venous oxygen saturation was

maintained. After rewarming, myocardial function was restored., CONCLUSIONS: In patients with normal left ventricular function, hypothermia impaired diastolic function. At increased heart rate, systolic function was subsequently reduced due to impeded filling. Changes in left ventricular function were rapidly reversed after rewarming. Copyright © 2021 by the Society of Critical Care Medicine and Wolters Kluwer Health, Inc. All Rights Reserved.

9. Jimenez-Cuja, R., Salazar-Orihuela, V., Llanco-Albornoz, L., Yap, S. H. K., Andersen, L. W., Granfeldt, A., … Smith, A. (2022). **Vasopressin and Methylprednisolone vs Placebo and Return of Spontaneous Circulation in Patients With In-Hospital Cardiac Arrest.** *JAMA*, *327*(5), 486–487. https://doi.org/https://dx.doi.org/10.1001/jama.2021.23051

10. Salamah A Faheem A, El Amrousy D, M. M. (2021). **Efficacy of Citicoline as a Neuroprotector in children with post cardiac arrest: a randomized controlled clinical trial**. *European Journal of Pediatrics*, *180*(4), 1249. Retrieved from http://ovidsp.ovid.com/ovidweb.cgi?T=JS&PAGE=reference&D=cctr&NEWS=N&AN=CN-02204781
Brain hypoxia after cardiac arrest leads to damage of the neuronal cell membrane. Citicoline is necessary for the synthesis of cell membrane. We planned to assess the neuroprotective effect of citicoline in children after cardiac arrest. This randomized controlled trial was carried out at pediatric intensive care units (PICU) and surgical ICU at Tanta university hospital on 80 consecutive children surviving in-hospital cardiac arrest who were subdivided into two groups. Group I (citicoline group) included 40 children with post-cardiac arrest who received citicoline 10 mg /kg /12 h IV for 6 weeks plus other supportive measures and group II (control group) included 40 children with post-cardiac arrest who were managed with only supportive measures. All patients were evaluated for Glasgow coma score (GCS), modified Rankin scale (mRS) for children, seizures frequency, type and duration, and serum neuron-specific enolase (NSE) before and 3 months after the treatment. GCS and mRS significantly improved in citicholine group compared to the control group. Seizure frequency and duration, mortality, PICU and hospital stay significantly decreased in citicholine group compared to the control group. Serum NSE levels significantly decreased in citicholine group only. No side effects were recorded. Conclusion: Citicoline is a promising neuroprotective drug in children with post-cardiac arrest. Trial Registration: The study was registered at Pan African Clinical Trials Registry (PACTR) www.pactr.samrc.ac.za with trial number PACTR201907742119058.What is known?* Post-resuscitation brain injury is one of the major complications that can lead to death or disability.* CDP-choline has been studied for acute ischemic stroke in several adult studies because of its reparative effect.What is new?* Our study was the first in pediatrics that assessed the neuroprotective effect of CDP-choline on the brain in children after cardiac arrest.* We found that Citicoline is a promising neuroprotective drug in children with post-cardiac arrest.

11. Wouters A Plessers S, Peeters R, Cappelle S, Demaerel P, Van Paesschen W, Ferdinande B, Dupont M, Dens J, Janssens S, Ameloot K, Lemmens R, S. L. (2021). **Added Value of Quantitative Apparent Diffusion Coefficient Values for Neuroprognostication After Cardiac Arrest**. *Neurology*, *96*(21), e2611. Retrieved from http://ovidsp.ovid.com/ovidweb.cgi?T=JS&PAGE=reference&D=cctr&NEWS=N&AN=CN-02263563
OBJECTIVE: To test the prognostic value of brain MRI in addition to clinical and electrophysiologic variables in patients post-cardiac arrest (CA), we explored data from the randomized Neuroprotect Post-CA trial (NCT02541591)., METHODS: In this trial, brain MRIs were prospectively obtained. We calculated receiver operating characteristic (ROC) curves for the average apparent diffusion coefficient (ADC) value and percentage of brain voxels with an ADC value <650 x 10-6 mm2/s and <450 x 10-6 mm2/s. We constructed multivariable logistic regression models with clinical characteristics, EEG, somatosensory evoked potentials (SSEP), and ADC value as independent variables to predict good neurologic recovery., RESULTS: In 79/102 patients, MRI data were available and in 58/79 patients all other data were available. At 180 days post-CA, 25/58 (43%) patients had good neurologic recovery. In univariable analysis of all tested MRI measures, average ADC value in the postcentral cortex had the highest accuracy to predict good neurologic recovery, with an area under the ROC curve (AUC) of 0.78. In the most optimal multivariable model, which also included corneal reflexes and EEG, this measure remained an independent predictor of good neurologic recovery (AUC 0.96, false-positive 27%). This model provided a more accurate prediction compared to the most optimal combination of EEG, corneal reflexes, and SSEP (p = 0.03)., CONCLUSIONS: Adding information on brain MRI in a multivariable model may improve the prediction of good neurologic recovery in patients post-CA., CLASSIFICATION OF EVIDENCE: This study provides Class III evidence that MRI ADC features predict neurologic recovery in patients post-CA.

12. D., C., A.E., A., K.F., T., & Krishnan. (2021). **Ketamine: Neuroprotective or Neurotoxic?** *Frontiers in Neuroscience*, *15*, 672526. https://doi.org/https://dx.doi.org/10.3389/fnins.2021.672526
Ketamine, a non-competitive N-methyl-D-aspartate receptor (NMDAR) antagonist, has been employed clinically as an intravenous anesthetic since the 1970s. More recently, ketamine has received attention for its rapid antidepressant effects and is actively being explored as a treatment for a wide range of neuropsychiatric syndromes. In model systems, ketamine appears to display a combination of neurotoxic and neuroprotective properties that are context dependent. At anesthetic doses applied during neurodevelopmental windows, ketamine contributes to inflammation, autophagy, apoptosis, and enhances levels of reactive oxygen species. At the same time, subanesthetic dose ketamine is a powerful activator of multiple parallel neurotrophic signaling cascades with neuroprotective actions that are not always NMDAR-dependent. Here, we summarize results from an array of preclinical studies that highlight a complex landscape of intracellular signaling pathways modulated by ketamine and juxtapose the somewhat contrasting neuroprotective and

neurotoxic features of this drug.© Copyright © 2021 Choudhury, Autry, Tolias and Krishnan.

13. I.O., I., B., B.-A., O.A., A., A.M., A., I.L., O., K.E., E., & Adeyemi O.O. AO - Ben-Azu, B. O. https://orcid.org/000.-0003-3569-3575. (2021). **Prevention and reversal of ketamine-induced experimental psychosis in mice by the neuroactive flavonoid, hesperidin: The role of oxidative and cholinergic mechanisms**. *Brain Research Bulletin*, *177*, 239–251. https://doi.org/https://dx.doi.org/10.1016/j.brainresbull.2021.10.007
Currently, prevailing evidence have identified cholinergic and oxidative pathways as important therapeutic targets for abating ketamine-induced schizophrenia-like behavior. Thus, this study evaluated the ability of hesperidin, a naturally occurring antioxidant and neuroprotective flavonoid, to prevent and reverse ketamine-induced schizophrenia-like behaviors and changes in cholinergic, oxidative and nitrergic status in mice. Forty-eight male Swiss mice were allotted into the preventive and reversal studies with 4 groups (n = 6) each. In the preventive study, groups 1 and 2 received vehicle (10 mL/kg/p.o./day), while groups 3 and 4 had hesperidin (100 mg/kg/p.o./day) for 14 days, but ketamine (20 mg/kg/i.p./day) was concurrently given to groups 2 and 4 from days 8-14. In the reversal study, groups 1 and 3 received vehicle, groups 2 and 4 were pretreated with ketamine for 14 days. Nevertheless, groups 3 and 4 additionally received hesperidin from days 8-14. Thereafter, schizophrenia-like behavior from exploratory activity, open-field (positive symptoms), Y-maze (cognitive symptoms) and social interaction (negative symptoms) tests were evaluated. Brain levels of oxidative/nitrergic (glutathione, superoxide-dismutase, malondialdehyde and nitrite levels) and cholinergic (acetylcholinesterase activity) markers were measured in the prefrontal-cortex, striatum and hippocampus. Hesperidin prevents and reverses ketamine-induced hyperactivities, social withdrawal and cognitive impairment. Also, hesperidin prevented and reversed ketamine-induced decrease in glutathione and superoxide-dismutase levels in the prefrontal-cortical, striatal and hippocampal brain regions in mice. Consequently, hesperidin attenuated ketamine-induced increase in malondialdehyde, nitrite levels and acetylcholinesterase activities in the prefrontal-cortex, striatum and hippocampus, respectively. The study showed that hesperidin prevents and reverses ketamine-induced schizophrenia-like behavior through inhibition of oxidative/nitrergic stress and acetylcholinesterase activity in mice brains. Therefore, these findings suggest that hesperidin dietary supplementation could provide natural nutritional intervention to protect against epigenetic-induced mental ill-health like schizophrenia, and thus serve as an important agent for nutritional psychiatry.Copyright © 2021 Elsevier Inc.

14. N.L., R., N.E., C., J.E., T., P.S., H., & Bayer K U. (2021). **CaMKIIalpha knockout protects from ischemic neuronal cell death after resuscitation from cardiac arrest**. *Brain Research*, *1773*, 147699. https://doi.org/https://dx.doi.org/10.1016/j.brainres.2021.147699
CaMKIIalpha plays a dual role in synaptic plasticity, as it can mediate synaptic changes in opposing directions. We hypothesized that CaMKIIalpha plays a similar dual role also in neuronal cell death and survival. Indeed, the CaMKII inhibitor tatCN21 is neuroprotective when added during or after excitotoxic/ischemic insults, but was described to cause sensitization when applied long-term prior to such insult. However, when comparing long-term CaMKII inhibition by several different inhibitors in neuronal cultures, we did not detect any sensitization. Likewise, in a mouse in vivo model of global cerebral ischemia (cardiac arrest followed by cardiopulmonary resuscitation), complete knockout of the neuronal CaMKIIalpha isoform did not cause sensitization but instead significant neuroprotection.Copyright © 2021 Elsevier B.V.

15. U., B., A., Di., G., C., R., U., G., M., G., K., … Akowuah E. (2021). **Neuroprotective strategies in acute aortic dissection: An analysis of the UK National Adult Cardiac Surgical Audit**. *European Journal of Cardio-Thoracic Surgery*, *60*(6), 1437–1444. https://doi.org/https://dx.doi.org/10.1093/ejcts/ezab192
OBJECTIVES: The risk of brain injury following surgery for type A aortic dissection (TAAD) remains substantial and no consensus has still been reached on which neuroprotective technique should be preferred. We aimed to investigate the association between neuroprotective strategies and clinical outcomes following TAAD repair. METHOD(S): Using the UK National Adult Cardiac Surgical Audit, we identified 1929 patients undergoing surgery for TAAD (2011-2018). Deep hypothermic circulatory arrest (DHCA) only, unilateral (uACP), bilateral antegrade cerebral perfusion (bACP) and retrograde cerebral perfusion were used in 830, 117, 760 and 222 patients, respectively. The primary end point was a composite of death and/or cerebrovascular accident (CVA). Generalized linear mixed model was used to adjust the effect of neuroprotective strategies for other confounders. RESULT(S): The use of bACP was associated with longer circulatory arrest (CA) compared to other strategies. There was a trend towards lower incidence of death and/or CVA using uACP only for shorter CA. In particular, primary end point rate was 27.7% overall and 26.5%, 12.5%, 28.0% and 22.9% for CA <30 min and 28.6%, 30.4%, 33.3% and 33.0% for CA >=30 min with DHCA only, uACP, bACP and retrograde cerebral perfusion, respectively. The use of DHCA only was associated with five-fold [odds ratio (OR) 5.35, 95% confidence interval (CI) 1.36-21.02] and two-fold (OR 1.77, 95% CI 1.01-3.09) increased risk of death and/or CVA compared to uACP and bACP, respectively, but the effect of uACP was significantly associated with CA duration (hazard ratio 0.97, 95% CI 0.94-0.99; P = 0.04). CONCLUSION(S): In TAAD repair, the use of uACP and bACP was associated with a lower adjusted risk of death and/or CVA when compared to DHCA. uACP can offer some advantage but only for a shorter CA duration. Copyright © 2021 The Author(s) 2021. Published by Oxford University Press on behalf of the European Association for Cardio-Thoracic Surgery.

16. J.R., H., E.L., F., A., F., A.K., A., R.P., B., K., J.-F., … Jackson T.C. AO - Herrmann Alicia K.; ORCID:

https://orcid.org/0000-0001-6734-1262 AO - Kochanek, Patrick M.; ORCID: https://orcid.org/0000-0002-2627-913X AO - Fabio, Anthony; ORCID: https://orcid.org/0000-0002-6808-4939, J. R. . O. https://orcid. org/000.-0002-3342-0189 A. O.-A. (2021). **Serum levels of the cold stress hormones FGF21 and GDF-15 after cardiac arrest in infants and children enrolled in single center therapeutic hypothermia clinical trials**. *Resuscitation*. https://doi.org/https://dx.doi.org/10.1016/j.resuscitation.2021.11.016
Objective: Fibroblast Growth Factor 21 (FGF21) and Growth Differentiation Factor-15 (GDF-15) are putative neuroprotective cold stress hormones (CSHs) provoked by cold exposure that may be age-dependent. We sought to characterize serum FGF21 and GDF-15 levels in pediatric cardiac arrest (CA) patients and their association with use of therapeutic hypothermia (TH). Method(s): Secondary analysis of serum samples from clinical trials. We measured FGF21 and GDF-15 levels in pediatric patients post-CA and compared levels to both pediatric intensive care (PICU) and healthy controls. Post-CA, we compared normothermia (NT) vs TH (33 degreeC for 72 h) treated cohorts at < 24 h, 24 h, 48 h, 72 h, and examined the change in CSHs over 72 h. We also assessed association between hospital mortality and initial levels. Result(s): We assessed 144 samples from 68 patients (27 CA [14 TH, 13 NT], 9 PICU and 32 healthy controls). Median initial FGF21 levels were higher post-CA vs. healthy controls (392 vs. 40 pg/mL, respectively, P < 0.001). Median GDF-15 levels were higher post-CA vs. healthy controls (7,089 vs. 396 pg/mL, respectively, P < 0.001). In the CA group, the median change in FGF21 from PICU day 1-3 (after 72 h of temperature control), was higher in TH vs. NT (231 vs. -20 pg/mL, respectively, P < 0.05), with no difference in GDF-15 over time. Serum GDF-15 levels were higher in CA patients that died vs. survived (19,450 vs. 5,337 pg/mL, respectively, P < 0.05), whereas serum FGF21 levels were not associated with mortality. Conclusion(s): Serum levels of FGF21 and GDF-15 increased after pediatric CA, and FGF21 appears to be augmented by TH.Copyright © 2021 Elsevier B.V.

17. I.F., B., B.S., B., O.I., R., V.P., R., O.G., A., & Makyeyeva L.V. AO - Belenichev Bogdan S.; ORCID: http://orcid.org/0000-0003-4539-7331 AO - Ryzhenko, Victor P.; ORCID: http://orcid.org/0000-0003-3466-7148 AO - Aliyeva, Olena G.; ORCID: http://orcid.org/0000-0003-1287-674X AO - Makyeyeva, Lyudmyla V.; ORCID: http://orcid.org/0000-0002-3188-2638, I. F. . O. http://orcid. org/000.-0003-1273-5314 A. O.-B. (2021). **Neuroprotective and anti-apoptotic activity of the il-1 antagonist rail-gel in rats after ketamine anesthesia**. *Pharmakeftiki*, *33*(2), 97–106. Retrieved from http://www.hsmc.gr/index.php/pharmakeftiki
Introduction: The choice of medicines for total intravenous anesthesia remains a relevant issue in practical anesthesiology. Ketamine is a well-known drug that has been widely used in the world, however its' effect on the CNS is debatable. It is reasonable to question the use of neuroprotective agents to protect against the negative effects of general anesthesia. Some studies have shown the neuroprotective activity of the RAIL. A new dosage form of RAIL-a gel for intranasal administration has been developed. This study was designed to evaluate the neuroprotective and anti-apoptotic activity of RAIL-gel in comparison with Citicoline and Piracetam during ketamine anesthesia Methods: In this study, 50 white nonlinear rats were randomly assigned to 5 groups: intact, ketamine anesthesia group, ketamine anesthesia + Piracetam (500 mg/kg, intraperitoneally) group, ketamine anesthesia + Citicoline (500 mg/kg, intraperitoneally) group, ketamine anesthesia + RAIL-gel (1 mg / kg intranasally) group. Expression of c-fos in the CA1 zone of the hippocampus and concentration of bcl-2 protein in the cytoplasmic fraction of the brain were determined by indirect immunofluorescence and Western blot analysis respectively. Result(s): Our research demonstrated the neurodegradative effect of ketamine anesthesia. The use of neuroprotective agents (Piracetam, Citicoline, RAIL-gel) in rats after general anesthesia led to a decrease in the neurodegradative effect of ketamine. The neuroprotective effect of RAIL-gel was significantly higher compared to reference drugs (p < 0.05). Conclusion(s): The neuroprotective effect of RAIL-gel is an experimental justification for further study of IL-1beta RAIL antagonist as a potential neuroprotective agent.Copyright © 2021, Zita Medical Managent. All rights reserved.

18. A.E., P., M.J., B., S.J., S., T., F., & Morrison H.W. AO - Pottenger Mitchell J.; ORCID: http://orcid.org/0000-0001-8280-5299 AO - Falk, Torsten; ORCID: http://orcid.org/0000-0003-4999-3309 AO - Morrison, Helena W.; ORCID: http://orcid.org/0000-0003-3791-0190, A. E. . O. http://orcid. org/000.-0002-0383-2975 A. O.-B. (2021). **Evaluation of microglia in a rodent model of Parkinson's disease primed with L-DOPA after sub-anesthetic ketamine treatment**. *Neuroscience Letters*, *765*, 136251. https://doi.org/http://dx.doi.org/10.1016/j.neulet.2021.136251
Parkinson's disease (PD) is a neurodegenerative disease caused by the death of dopaminergic neurons in the substantia nigra pars compacta (SNpc), characterized by motor dysfunction. While PD symptoms are well treated with L-DOPA, continuous use can cause L-DOPA-induced dyskinesia (LID). We have previously demonstrated that sub-anesthetic ketamine attenuated LID development in rodents, measured by abnormal involuntary movements (AIMs), and reduced the density of maladaptive striatal dendritic mushroom spines. Microglia may play a role by phagocytosing maladaptive neuronal spines. In this exploratory study, we hypothesized that ketamine would prevent AIMs and change microglia ramified morphology - an indicator of a microglia response. Unilaterally 6-hydroxydopamine (6-OHDA)-lesioned rats were primed with daily injections of L-DOPA for 14 days, treated on days 0 and 7 for 10-hours with sub-anesthetic ketamine (i.p.), and we replicated that this attenuated LID development. We further extended our prior work by showing that while ketamine treatment did lead to an increase of striatal interleukin-6 in dyskinetic rats, indicating a modulation of an inflammatory response, it did not change microglia number or morphology in the dyskinetic striatum. Yet an increase of CD68 in the SNpc of 6-OHDA-lesioned hemispheres post-ketamine indicates increased microglia phagocytosis suggestive of a lingering microglial response to 6-OHDA injury in the SNpc pointing to possible anti-inflammatory action in the PD

model in addition to anti-dyskinetic action. In conclusion, we provide further support for sub-anesthetic ketamine treatment of LID. The mechanisms of action for ketamine, specifically related to inflammation and microglia phagocytic functions, are emerging, and require further examination.Copyright © 2021 Elsevier B.V.

19. A.H., M., K., M., B., B., S.R.M., S., & Zare M. (2021). **Neuroprotective effects of curcumin-loaded nanophytosome on ketamine-induced schizophrenia-like behaviors and oxidative damage in male mice**. *Neuroscience Letters*, *765*, 136249. https://doi.org/http://dx.doi.org/10.1016/j.neulet.2021.136249
Curcumin as an antioxidant natural herb has shown numerous pharmacological effects. However, the poor bioavailability of curcumin is a significant pharmacological barrier for its antioxidant activities. The present study was conducted to develop curcumin-loaded nanophytosome (CNP) and explore their therapeutic potential in a ketamine (KET)-induced schizophrenia (SCZ) model. The mice in our experiment were treated orally with curcumin and CNP (20 mg/kg) for 30 consecutive days. In addition, the animals received intraperitoneal injection of KET (30 mg/kg/day) from the 16th to the 30th day. SCZ-like behaviors were evaluated employing forced swimming test (FST), open field test (OFT), and novel object recognition test (NORT), and oxidative stress markers in the brain were estimated. Our results revealed that CNP has a greater neuroprotective effect compared to free curcumin. CNP pretreatment significantly ameliorated KET-induced brain injury evidenced by a marked reduction in the depressive and anxiety-like behaviors, memory deficits, and oxidative stress markers in cortical and subcortical tissues. Therefore, CNP, as a suitable drug delivery system, may improve curcumin bioavailability and confer stronger neuroprotective effects against KET-induced behavioral deficits and oxidative damages.Copyright © 2021 Elsevier B.V.

20. R.C., C., M., S., S., S., D.M., R., D., J., S.J., M., … Becker L B. (2021). **Pharmacological Approach for Neuroprotection After Cardiac Arrest-A Narrative Review of Current Therapies and Future Neuroprotective Cocktail**. *Frontiers in Medicine*, *8*, 636651. https://doi.org/http://dx.doi.org/10.3389/fmed.2021.636651
Cardiac arrest (CA) results in global ischemia-reperfusion injury damaging tissues in the whole body. The landscape of therapeutic interventions in resuscitation medicine has evolved from focusing solely on achieving return of circulation to now exploring options to mitigate brain injury and preserve brain function after CA. CA pathology includes mitochondrial damage and endoplasmic reticulum stress response, increased generation of reactive oxygen species, neuroinflammation, and neuronal excitotoxic death. Current non-pharmacologic therapies, such as therapeutic hypothermia and extracorporeal cardiopulmonary resuscitation, have shown benefits in protecting against ischemic brain injury and improving neurological outcomes post-CA, yet their application is difficult to institute ubiquitously. The current preclinical pharmacopeia to address CA and the resulting brain injury utilizes drugs that often target singular pathways and have been difficult to translate from the bench to the clinic. Furthermore, the limited combination therapies that have been attempted have shown mixed effects in conferring neuroprotection and improving survival post-CA. The global scale of CA damage and its resultant brain injury necessitates the future of CA interventions to simultaneously target multiple pathways and alleviate the hemodynamic, mitochondrial, metabolic, oxidative, and inflammatory processes in the brain. This narrative review seeks to highlight the current field of post-CA neuroprotective pharmaceutical therapies, both singular and combination, and discuss the use of an extensive multi-drug cocktail therapy as a novel approach to treat CA-mediated dysregulation of multiple pathways, enhancing survival, and neuroprotection.© Copyright © 2021 Choudhary, Shoaib, Sohnen, Rolston, Jafari, Miyara, Hayashida, Molmenti, Kim and Becker.

21. Wright D R. (2021). **Ketamine in Modern Neuroanesthesia Practice**. *Current Anesthesiology Reports*, *11*(3), 189–194. https://doi.org/http://dx.doi.org/10.1007/s40140-021-00465-2
Purpose of Review: Ketamine has a number of clinical uses and properties that suggest a role for the drug in neuroanestheisa practice. "Dogma" and "myths" persist with regard to its effects on cerebral hemodynamics and intracranial pressure which have limited its use in Neuroanesthesia and care of the critically ill brain-injured patient. This review aims to educate the clinician on the possible role of ketamine in modern neuroanesthesia practice. Recent Findings: A number of systemic reviews support the use of ketamine in patients with acute brain injury and raised intracranial pressure (ICP). Pre-clinical work suggests that ketamine may have mechanisms of action compatible with neuroprotection including modifying glutamate excitatory-driven mechanisms of brain injury. There is emerging clinical evidence to suggest that ketamine may inhibit spreading depolarizations (SDs), a cortical electrical phenomenon associated with brain injury. Summary: Ketamine is no longer contraindicated in the care of the brain-injured patient, and its properties of potent analgesia, dissociative anesthesia, and minimal effects on both the hemodynamic and respiratory system are being utilized in the pre-hospital and emergency room setting. Good grade data on meaningful clinical outcomes is presently lacking to support the use of ketamine as a drug with neuroprotection properties but is an area of ongoing interest.Copyright © 2021, The Author(s), under exclusive licence to Springer Science+Business Media, LLC, part of Springer Nature.

22. H., S., P., P., G., W., J., W., Y., W., & Huang H. (2021). **Neuroprotective Effects of Dexmedetomidine on the Ketamine-Induced Disruption of the Proliferation and Differentiation of Developing Neural Stem Cells in the Subventricular Zone**. *Frontiers in Pediatrics*, *9*, 649284. https://doi.org/http://dx.doi.org/10.3389/fped.2021.649284
Background: Ketamine disrupts the proliferation and differentiation of developing neural stem cells (NSCs). Therefore,

the safe use of ketamine in pediatric anesthesia has been an issue of increasing concern among anesthesiologists and children's parents. Dexmedetomidine (DEX) is widely used in sedation as an antianxiety agent and for analgesia. DEX has recently been shown to provide neuroprotection against anesthetic-induced neurotoxicity in the developing brain. The aim of this in vivo study was to investigate whether DEX exerted neuroprotective effects on the proliferation and differentiation of NSCs in the subventricular zone (SVZ) following neonatal ketamine exposure. Method(s): Postnatal day 7 (PND-7) male Sprague-Dawley rats were equally divided into the following five groups: control group (n = 8), ketamine group (n = 8), 1 mug/kg DEX+ketamine group (n = 8), 5 mug/kg DEX+ketamine group (n = 8) and 10 mug/kg DEX+ketamine group (n = 8). Immediately after treatment, rats received a single intraperitoneal injection of BrdU, and the proliferation and differentiation of NSCs in the SVZ were assessed using immunostaining at 24 h after the BrdU injection. In the olfactory behavioral tests, rats in each group were raised until 2 months old, and the buried food test and olfactory memory test were performed. Result(s): The proliferation of NSCs and astrocytic differentiation in the SVZ were significantly inhibited at 24 h after repeated ketamine exposure in the neonatal period, and neuronal differentiation was markedly increased. Furthermore, pretreatment with moderately high (5 mug/kg) or high doses (10 mug/kg) of DEX reversed ketamine-induced disturbances in the proliferation and differentiation of NSCs. In the behavior tests, repeated neonatal ketamine exposure induced olfactory cognitive dysfunction in the adult stage, and moderately high and high doses of DEX reversed the olfactory cognitive dysfunction induced by ketamine. Conclusion(s): Based on the present findings, pretreatment with a moderately high (5 mug/kg) or high dose (10 mug/kg) of DEX may alleviate the developmental neurogenesis disorder in the SVZ at 24 h after repeated ketamine exposure and improve olfactory cognitive dysfunction in adulthood.© Copyright © 2021 Sha, Peng, Wei, Wang, Wu and Huang.

23. A., G., A., N.-C., I., E., S., B., R., G., R., E., … Lazar S. (2021). **Neuroprotection by delayed triple therapy following sarin nerve agent insult in the rat**. *Toxicology and Applied Pharmacology*, *419*, 115519. https://doi.org/http://dx.doi.org/10.1016/j.taap.2021.115519
The development of refractory status epilepticus (SE) induced by sarin intoxication presents a therapeutic challenge. In our current research we evaluate the efficacy of a delayed combined triple treatment in ending the abnormal epileptiform seizure activity (ESA) and the ensuing of long-term neuronal insult. SE was induced in male Sprague-Dawley rats by exposure to 1.2LD50 sarin insufficiently treated by atropine and TMB4 (TA) 1 min later. Triple treatment of ketamine, midazolam and valproic acid was administered 30 min or 1 h post exposure and was compared to a delayed single treatment with midazolam alone. Toxicity and electrocorticogram activity were monitored during the first week and behavioral evaluation performed 3 weeks post exposure followed by brain biochemical and immunohistopathological analyses. The addition of both single and triple treatments reduced mortality and enhanced weight recovery compared to the TA-only treated group. The triple treatment also significantly minimized the duration of the ESA, reduced the sarin-induced increase in the neuroinflammatory marker PGE2, the brain damage marker TSPO, decreased the gliosis, astrocytosis and neuronal damage compared to the TA+ midazolam or only TA treated groups. Finally, the triple treatment eliminated the sarin exposed increased open field activity, as well as impairing recognition memory as seen in the other experimental groups. The delayed triple treatment may serve as an efficient therapy, which prevents brain insult propagation following sarin-induced refractory SE, even if treatment is postponed for up to 1 h.Copyright © 2021 Elsevier Inc.

24. T., C., M., T., P., J., B., Z., X., W., Q., C., … Cai H. (2021). **A Potential Mechanism Underlying the Therapeutic Effects of Progesterone and Allopregnanolone on Ketamine-Induced Cognitive Deficits**. *Frontiers in Pharmacology*, *12*, 612083. https://doi.org/https://dx.doi.org/10.3389/fphar.2021.612083
Ketamine exposure can model cognitive deficits associated with schizophrenia. Progesterone (PROG) and its active metabolite allopregnanolone (ALLO) have neuroprotective effects and the pathway involving progesterone receptor membrane component 1 (PGRMC1), epidermal growth factor receptor (EGFR), glucagon-like peptide-1 receptor (GLP-1R), phosphatidylinositol 3 kinase (PI3K), and protein kinase B (Akt) appears to play a key role in their neuroprotection. The present study aimed to investigate the effects of PROG (8,16 mg kg-1) and ALLO (8,16 mg kg-1) on the reversal of cognitive deficits induced by ketamine (30 mg kg-1) via the PGRMC1 pathway in rat brains, including hippocampus and prefrontal cortex (PFC). Cognitive performance was evaluated by Morris water maze (MWM) test. Western blot and real-time quantitative polymerase chain reaction were utilized to assess the expression changes of protein and mRNA. Additionally, concentrations of PROG and ALLO in plasma, hippocampus and PFC were measured by a liquid chromatography-tandem mass spectrometry method. We demonstrated that PROG or ALLO could reverse the impaired spatial learning and memory abilities induced by ketamine, accompanied with the upregulation of PGRMC1/EGFR/GLP-1R/PI3K/Akt pathway. Additionally, the coadministration of AG205 abolished their neuroprotective effects and induced cognitive deficits similar with ketamine. More importantly, PROG concentrations were markedly elevated in PROG-treated groups in hippocampus, PFC and plasma, so as for ALLO concentrations in ALLO-treated groups. Interestingly, ALLO (16 mg kg-1) significantly increased the levels of PROG. These findings suggest that PROG can exert its neuroprotective effects via activating the PGRMC1/EGFR/GLP-1R/PI3K/Akt pathway in the brain, whereas ALLO also restores cognitive deficits partially via increasing the level of PROG in the brain to activate the PGRMC1 pathway.© Copyright © 2021 Cao, Tang, Jiang, Zhang, Wu, Chen, Zeng, Li, Zhang and Cai.

25. Q., M., L., F., T., W., Y., L., Z., L., B., Z., … Fan D. (2021). **2020 expert consensus statement on neuro-protection after cardiac arrest in China**. *Annals of Translational Medicine*, *9*(2), 175.

https://doi.org/https://dx.doi.org/10.21037/atm-20-7853

26. K., G., E., E., X., Z., C., L., A., S.-P., R., S., … Lawton J. AO - Giuliano Xun; ORCID: https://orcid.org/0000-0001-5179-5032, K. O. https://orcid. org/000.-0003-2260-1854 A. O.-Z. (2021). **NMDA Receptor Antagonism for Neuroprotection in a Canine Model of Hypothermic Circulatory Arrest**. *Journal of Surgical Research*, *260*, 177–189. https://doi.org/https://dx.doi.org/10.1016/j.jss.2020.11.075
Background: Hypothermic circulatory arrest (HCA) is associated with neurologic morbidity, in part mediated by activation of the N-methyl-D-aspartate glutamate receptor causing excitotoxicity and neuronal apoptosis. Using a canine model, we hypothesized that the N-methyl-D-aspartate receptor antagonist MK801 would provide neuroprotection and that MK801 conjugation to dendrimer nanoparticles would improve efficacy. Material(s) and Method(s): Male hound dogs were placed on cardiopulmonary bypass, cooled to 18degreeC, and underwent 90 min of HCA. Dendrimer conjugates (d-MK801) were prepared by covalently linking dendrimer surface OH groups to MK801. Six experimental groups received either saline (control), medium- (0.15 mg/kg) or high-dose (1.56 mg/kg) MK801, or low- (0.05 mg/kg), medium-, or high-dose d-MK801. At 24, 48, and 72 h after HCA, animals were scored by a standardized neurobehavioral paradigm (higher scores indicate increasing deficits). Cerebrospinal fluid was obtained at baseline, eight, 24, 48, and 72 h after HCA. At 72 h, brains were examined for histopathologic injury in a blinded manner (higher scores indicate more injury). Result(s): Neurobehavioral deficit scores were reduced by low-dose d-MK801 on postoperative day two ($P < 0.05$) and by medium-dose d-MK801 on postoperative day 3 ($P = 0.05$) compared with saline controls, but free drug had no effect. In contrast, high-dose free MK801 significantly improved histopathology scores compared with saline ($P < 0.05$) and altered biomarkers of injury in cerebrospinal fluid, with a significant reduction in phosphorylated neurofilament-H for high-dose MK801 versus saline ($P < 0.05$). Conclusion(s): Treatment with MK-801 demonstrated significant improvement in neurobehavioral and histopathology scores after HCA, although not consistently across doses and conjugates.Copyright © 2020 Elsevier Inc.

27. Morris, P. J., Burke, R. D., Sharma, A. K., Lynch, D. C., Lemke-Boutcher, L. E., Mathew, S., … Thomas, C. J. (2021). **A comparison of the pharmacokinetics and NMDAR antagonism-associated neurotoxicity of ketamine, (2R,6R)-hydroxynorketamine and MK-801.** *Neurotoxicology and Teratology*, *87*, 106993. https://doi.org/https://dx.doi.org/10.1016/j.ntt.2021.106993
With the increasing use of ketamine as an off-label treatment for depression and the recent FDA approval of (S)-ketamine for treatment-resistant depression, there is an increased need to understand the long-term safety profile of chronic ketamine administration. Of particular concern is the neurotoxicity previously observed in rat models following acute exposure to high doses of ketamine, broadly referred to as "Olney's lesions". This type of toxicity presents as abnormal neuronal cellular vacuolization, followed by neuronal death and has been associated with ketamine's inhibition of the N-methyl-d-aspartate receptor (NMDAR). In this study, a pharmacological and neuropathological analysis of ketamine, the potent NMDAR antagonist MK-801, and the ketamine metabolite (2R,6R)-hydroxynorketamine [(2R,6R)-HNK)] in rats is described following both single dose and repeat dose drug exposures. Ketamine dosing was studied up to 20 mg/kg intravenously for the single-dose neuropathology study and up to 60 mg/kg intraperitoneally for the multiple-dose neuropathology study. MK-801 dosing was studied up to 0.8 mg/kg subcutaneously for both the single and multiple-dose neuropathology studies, while (2R,6R)-HNK dosing was studied up to 160 mg/kg intravenously in both studies. These studies confirm dose-dependent induction of "Olney's lesions" following both single dose and repeat dosing of MK-801. Ketamine exposure, while showing common behavioral effects, did not induce wide-spread Olney's lesions. Treatment with (2R,6R)-HNK did not produce behavioral effects, toxicity or any evidence of Olney's lesion formation. Based on these results, future NMDAR-antagonist neurotoxicity studies should strongly consider taking pharmacokinetics more thoroughly into account. Copyright Published by Elsevier Inc.

28. Chen, M. W., Santos, P., Kulikowicz, E., Koehler, R. C., Lee, J. K., & Martin, L. J. (2021). **Targeting the mitochondrial permeability transition pore for neuroprotection in a piglet model of neonatal hypoxic-ischemic encephalopathy.** *Journal of Neuroscience Research*, *99*(6), 1550–1564. https://doi.org/https://dx.doi.org/10.1002/jnr.24821
Neonatal hypoxic-ischemic encephalopathy (HIE) causes significant morbidity despite treatment with therapeutic hypothermia. Mitochondrial dysfunction may drive the mechanisms underlying neuronal cell death, thereby making mitochondria prime targets for neuroprotection. The mitochondrial permeability transition pore (mPTP) is one such target within mitochondria. In adult animal models, mPTP inhibition is neuroprotective. However, evidence for mPTP inhibition in neonatal models of neurologic disease is less certain. We tested the therapeutic efficacy of the mPTP small molecule inhibitor GNX-4728 and examined the developmental presence of brain mPTP proteins for drug targeting in a neonatal piglet model of hypoxic-ischemic brain injury. Male neonatal piglets were randomized to hypoxia-ischemia (HI) or sham procedure with GNX-4728 (15 mg/kg, IV) or vehicle (saline/cyclodextrin/DMSO, IV). GNX-4728 was administered as a single dose within 5 min after resuscitation from bradycardic arrest. Normal, ischemic, and injured neurons were counted in putamen and somatosensory cortex using hematoxylin and eosin staining. In separate neonatal and juvenile pigs, western blots of putamen mitochondrial-enriched fractions were used to evaluate mitochondrial integrity and the presence of mPTP proteins. We found that a single dose of GNX-4728 did not protect putamen and cortical neurons from cell death after HI. However, loss of mitochondrial matrix integrity occurred within 6h after HI, and while mPTP components are present in the neonatal brain their levels were significantly different compared to that of a

mature juvenile brain. Thus, the neonatal brain mPTP may not be a good target for current neurotherapeutic drugs that are developed based on adult mitochondria. Copyright © 2021 Wiley Periodicals LLC.

29. Davis-Reyes, B. D., Smith, A. E., Xu, J., Cunningham, K. A., Zhou, J., & Anastasio, N. C. (2021). **Subanesthetic ketamine with an AMPAkine attenuates motor impulsivity in rats.** *Behavioural Pharmacology*, *32*(4), 335–344. https://doi.org/https://dx.doi.org/10.1097/FBP.0000000000000623
The concept of "impulse control" has its roots in early psychiatry and today has progressed into a well-described, although poorly understood, multidimensional endophenotype underlying many neuropsychiatric disorders (e.g., attention deficit hyperactivity disorder, schizophrenia, substance use disorders). There is mounting evidence suggesting that the cognitive and/or behavioral dimensions underlying impulsivity are driven by dysfunctional glutamate (Glu) neurotransmission via targeted ionotropic Glu receptor (GluR) [e.g., N-methyl-D-aspartate receptor (NMDAR), alpha-amino-3-hydroxy-5-methyl-4-isoxazolepropionic acid receptor (AMPAR)] mechanisms and associated synaptic alterations within key brain nodes. Ketamine, a noncompetitive NMDAR antagonist and FDA-approved for treatment-resistant depression, induces a "glutamate burst" that drives resculpting of the synaptic milieu, which lasts for several days to a week. Thus, we hypothesized that single and repeated treatment with a subanesthetic ketamine dose would normalize motor impulsivity. Next, we hypothesized that AMPAR positive allosteric modulation, alone or in combination with ketamine, would attenuate impulsivity and provide insight into the mechanisms underlying GluR dysfunction relevant to motor impulsivity. To measure motor impulsivity, outbred male Sprague-Dawley rats were trained on the one-choice serial reaction time task. Rats pretreated with single or repeated (3 days) administration of ketamine (10 mg/kg; i.p.; 24-h pretreatment) or with the AMPAkine HJC0122 (1 or 10 mg/kg; i.p.; 30-min pretreatment) exhibited lower levels of motor impulsivity vs. control. Combination of single or repeated ketamine plus HJC0122 also attenuated motor impulsivity vs. control. We conclude that ligands designed to promote GluR signaling represent an effective pharmacological approach to normalize impulsivity and subsequently, neuropsychiatric disorders marked by aberrant impulse control. Copyright © 2021 Wolters Kluwer Health, Inc. All rights reserved.

30. Liu, W., Ye, Q., Xi, W., Li, Y., Zhou, X., Wang, Y., … Hai, K. (2021). **The ERK/CREB/PTN/syndecan-3 pathway involves in heparin-mediated neuro-protection and neuro-regeneration against cerebral ischemia-reperfusion injury following cardiac arrest.** *International Immunopharmacology*, *98*, 107689. https://doi.org/https://dx.doi.org/10.1016/j.intimp.2021.107689
BACKGROUND: Heparin, a commonly used anticoagulant, has been found to improve cerebral ischemia-reperfusion injury (CIR-CA) following cardiopulmonary resuscitation (CPR). Here, we aimed to explore the role of pleiotrophin (PTN)/syndecan-3 pathway in heparin therapy for CIR-CA., MATERIALS AND METHODS: The CA-CPR model was constructed in Sprague-Dawley (SD) rats, which were treated with low molecular weight heparin, and the neurological changes and brain histopathological changes were evaluated. For in-vitro experiments, the ischemic injury model of primary neurons was established by oxygen and glucose deprivation (OGD), and the neuron regeneration was detected via the Cell counting Kit-8 (CCK8) method, flow cytometry and microscopy. CREB antagonist (KG-501), ERK antagonist (PD98059) and si-PTN were used respectively to inhibit the expression of CREB, ERK and PTN in cells, so as to explore the role of heparin in regulating neuronal regeneration., RESULTS: Compared with the sham rats, the neurological deficits and cerebral edema of CA-CPR rats were significantly improved after heparin treatment. Heparin also attenuated OGD-mediated neuronal apoptosis and promoted neurite outgrowth in vitro. Moreover, heparin attenuated CA-CPR-mediated neuronal apoptosis and microglial neuroinflammation. In terms of the mechanism, heparin upregulated the expression of ERK, CREB, NF200, BDNF, NGF, PTN and syndecan-3 in the rat brains. Inhibition of ERK, CREB and interference with PTN expression notably weakened the heparin-mediated neuroprotective effects and restrained the expression of ERK/CREB and PTN/syndecan-3 pathway., CONCLUSION: Heparin attenuates the secondary brain injury induced by CA-CPR through regulating the ERK/CREB-mediated PTN/syndecan-3 pathway. Copyright © 2021. Published by Elsevier B.V.

31. Li, G., LeiQian, Gu, P., & Fan, D. (2021). **Dexmedetomidine post-conditioning attenuates cerebral ischemia following asphyxia cardiac arrest through down-regulation of apoptosis and neuroinflammation in rats.** *BMC Anesthesiology*, *21*(1), 180. https://doi.org/https://dx.doi.org/10.1186/s12871-021-01394-7
BACKGROUND: Neuroprotection strategies after cardiac arrest (CA)/cardiopulmonary resuscitation (CPR) remain key areas of basic and clinical research. This study was designed to investigate the neuroprotective effects of dexmedetomidine following resuscitation and potential mechanisms., METHODS: Anesthetized rats underwent 6-min asphyxia-based cardiac arrest and resuscitation, after which the experimental group received a single intravenous dose of dexmedetomidine (25 mug/kg). Neurological outcomes and ataxia were assessed after the return of spontaneous circulation. The serum levels and brain expression of inflammation markers was examined, and apoptotic cells were quantified by TUNEL staining., RESULTS: Neuroprotection was enhanced by dexmedetomidine post-conditioning after the return of spontaneous circulation. This enhancement was characterized by the promotion of neurological function scores and coordination. In addition, dexmedetomidine post-conditioning attenuated the serum levels of the pro-inflammatory cytokine tumor necrosis factor (TNF)-alpha at 2 h, as well as interleukin IL-1beta at 2, 24, and 48 h. TUNEL staining showed that the number of apoptotic cells in the dexmedetomidine post-conditioning group was significantly reduced compared with the control group. Further western blot analysis indicated that dexmedetomidine markedly reduced the levels of caspase-3 and nuclear factor-kappa B (NF-kappaB) in the brain., CONCLUSIONS:

Dexmedetomidine post-conditioning had a neuroprotective effect against cerebral injury following asphyxia-induced cardiac arrest. The mechanism was associated with the downregulation of apoptosis and neuroinflammation.

32. Madsen, F. A., Andreasen, T. H., Lindschou, J., Gluud, C., & Moller, K. (2021). **Ketamine for critically ill patients with severe acute brain injury: Protocol for a systematic review with meta-analysis and Trial Sequential Analysis of randomised clinical trials.** *PloS One*, *16*(11), e0259899. https://doi.org/https://dx.doi.org/10.1371/journal.pone.0259899
INTRODUCTION: Intensive care for patients with severe acute brain injury aims both to treat the immediate consequences of the injury and to prevent and treat secondary brain injury to ensure a good functional outcome. Sedation may be used to facilitate mechanical ventilation, for treating agitation, and for controlling intracranial pressure. Ketamine is an N-methyl-D-aspartate receptor antagonist with sedative, analgesic, and potentially neuroprotective properties. We describe a protocol for a systematic review of randomised clinical trials assessing the beneficial and harmful effects of ketamine for patients with severe acute brain injury., METHODS AND ANALYSIS: We will systematically search international databases for randomised clinical trials, including CENTRAL, MEDLINE, Embase, and trial registries. Two authors will independently review and select trials for inclusion, and extract data. We will compare ketamine by any regimen versus placebo, no intervention, or other sedatives or analgesics for patients with severe acute brain injury. The primary outcomes will be functional outcome at maximal follow up, quality of life, and serious adverse events. We will also assess secondary and exploratory outcomes. The extracted data will be analysed using Review Manager and Trials Sequential Analysis. Evidence certainty will be graded using GRADE., ETHICS AND DISSEMINATION: The results of the systematic review will be disseminated through peer-reviewed publication. With the review, we hope to inform future randomised clinical trials and improve clinical practice., PROSPERO NO: CRD42021210447.

33. Chen, Y., Xu, W., Yuan, Y., Chen, H., Zheng, S., He, Y., & Luo, T. (2021). **N-Methyl-d-aspartic Acid (NMDA) Receptor Is Involved in the Inhibitory Effect of Ketamine on Human Sperm Functions.** *International Journal of Molecular Sciences*, *22*(22). https://doi.org/https://dx.doi.org/10.3390/ijms222212370
Ketamine, which used to be widely applied in human and animal medicine as a dissociative anesthetic, has become a popular recreational drug because of its hallucinogenic effect. Our previous study preliminarily proved that ketamine could inhibit human sperm function by affecting intracellular calcium concentration ([Ca2+]i). However, the specific signaling pathway of [Ca2+]i induced by ketamine in human sperm is still not clear yet. Here, the N-methyl-d-aspartic acid (NMDA) receptor was detected in the tail region of human sperm. Its physiological ligand, NMDA (50 muM), could reverse ketamine's inhibitory effect on human sperm function, and its antagonist, MK801 (100 muM), could restrain the effect of NMDA. The inhibitory effect caused by 4 mM ketamine or 100 muM MK801 on [Ca2+]i, which is a central factor in the regulation of human sperm function, could also be recovered by 50 muM NMDA. The results suggest that the NMDA receptor is probably involved in the inhibitory effect of ketamine on human sperm functions.

34. Zhang, Z., Liu, W., Shen, M., Ma, X., Li, R., Jin, X., … Gao, L. (2021). **Protective Effect of GM1 Attenuates Hippocampus and Cortex Apoptosis After Ketamine Exposure in Neonatal Rat via PI3K/AKT/GSK3beta Pathway.** *Molecular Neurobiology*, *58*(7), 3471–3483. https://doi.org/https://dx.doi.org/10.1007/s12035-021-02346-5
Ketamine is a widely used analgesic and anesthetic in obstetrics and pediatrics. Ketamine is known to promote neuronal death and cognitive dysfunction in the brains of humans and animals during development. Monosialotetrahexosyl ganglioside (GM1), a promoter of brain development, exerts neuroprotective effects in many neurological disease models. Here, we investigated the neuroprotective effect of GM1 and its potential underlying mechanism against ketamine-induced apoptosis of rats. Seven-day-old Sprague Dawley (SD) rats were randomly divided into the following four groups: (1) group C (control group: normal saline was injected intraperitoneally); (2) group K (ketamine); (3) group GM1 (GM1 was given before normal saline injection); and (4) GM1+K group (received GM1 30 min before continuous exposure to ketamine). Each group contained 15 rats, received six doses of ketamine (20 mg/kg), and was injected with saline every 90 min. The Morris water maze (MWM) test, the number of cortical and hippocampal cells, apoptosis, and AKT/GSK3beta pathway were analyzed. To determine whether GM1 exerted its effect via the PI3K/AKT/GSK3beta pathway, PC12 cells were incubated with LY294002, a PI3K inhibitor. We found that GM1 protected against ketamine-induced apoptosis in the hippocampus and cortex by reducing the expression of Bcl-2 and Caspase-3, and by increasing the expression of Bax. GM1 treatment increased the expression of p-AKT and p-GSK3beta. However, the anti-apoptotic effect of GM1 was eliminated after inhibiting the phosphorylation of AKT. We showed that GM1 lessens ketamine-induced apoptosis in the hippocampus and cortex of young rats by regulating the PI3K/AKT/GSK3beta pathway. Taken together, GM1 may be a potential preventive treatment for the neurotoxicity caused by continuous exposure to ketamine.

35. Piva, A., Caffino, L., Mottarlini, F., Pintori, N., Castillo Diaz, F., Fumagalli, F., & Chiamulera, C. (2021). **Metaplastic Effects of Ketamine and MK-801 on Glutamate Receptors Expression in Rat Medial Prefrontal Cortex and Hippocampus.** *Molecular Neurobiology*, *58*(7), 3443–3456. https://doi.org/https://dx.doi.org/10.1007/s12035-021-02352-7
Ketamine and MK-801 by blocking NMDA receptors may induce reinforcing effects as well as schizophrenia-like symptoms. Recent results showed that ketamine can also effectively reverse depressive signs in patients' refractory to standard therapies. This evidence clearly points to the need of characterization of effects of these NMDARs antagonists

on relevant brain areas for mood disorders. The aim of the present study was to investigate the molecular changes occurring at glutamatergic synapses 24 h after ketamine or MK-801 treatment in the rat medial prefrontal cortex (mPFC) and hippocampus (Hipp). In particular, we analyzed the levels of the glutamate transporter-1 (GLT-1), NMDA receptors, AMPA receptors subunits, and related scaffolding proteins. In the homogenate, we found a general decrease of protein levels, whereas their changes in the post-synaptic density were more complex. In fact, ketamine in the mPFC decreased the level of GLT-1 and increased the level of GluN2B, GluA1, GluA2, and scaffolding proteins, likely indicating a pattern of enhanced excitability. On the other hand, MK-801 only induced sparse changes with apparently no correlation to functional modification. Differently from mPFC, in Hipp, both substances reduced or caused no changes of glutamate receptors and scaffolding proteins expression. Ketamine decreased NMDA receptors while increased AMPA receptors subunit ratios, an effect indicative of permissive metaplastic modulation; conversely, MK-801 only decreased the latter, possibly representing a blockade of further synaptic plasticity. Taken together, these findings indicate a fine tuning of glutamatergic synapses by ketamine compared to MK-801 both in the mPFC and Hipp.

36. Andrade, Y. C. P., Ropke, J., Viana, T. G., Fanelli, C., Minaldi, E., Batista, L. A., … Moreira, F. A. (2021). **Effects of JL13, a pyridobenzoxazepine compound, in dopaminergic and glutamatergic models of antipsychotic activity.** *Behavioural Pharmacology*, *32*(1), 2–8. https://doi.org/https://dx.doi.org/10.1097/FBP.0000000000000595
The pyridobenzoxazepine compound, 5-(4-methylpiperazin-1-yl)-8-chloro-pyrido[2,3-b][1,5]benzoxazepine (JL13), has been developed as a potential antipsychotic drug. We tested the hypothesis that JL13 is efficacious in both dopaminergic and glutamatergic animal models of schizophrenia. We investigated JL13 for its efficacy to prevent cocaine- and ketamine-induced hyperlocomotion and MK-801-induced deficits in prepulse inhibition (PPI) of the startle reflex. Male Swiss mice received injections of JL13 (0.1-10 mg/kg) and were tested in the open field for basal locomotion. In separate experiments, the animals received injections of JL13 (0.1-3 mg/kg) followed by cocaine (10 mg/kg), ketamine (60 mg/kg), or MK-801 (0.5 mg/kg) and were tested in the open field for hyperlocomotion. In addition, it was also tested if JL13 prevented MK-801-induced disruption of PPI. Only the highest dose of JL13 impaired spontaneous locomotion, suggesting its favorable profile regarding motor side effects. At doses that did not impair basal motor activity, JL13 prevented cocaine-, ketamine-, and MK-801-induced hyperlocomotion. Moreover, JL13 prevented MK-801-induced disruption of PPI. Extending previous findings, this study shows that JL13 exerts antipsychotic-like activity in both dopaminergic and glutamatergic models. This compound has a favorable pharmacological profile, similar to second-generation antipsychotics. Copyright © 2020 Wolters Kluwer Health, Inc. All rights reserved.

37. Wiklund, L., Sharma, A., Patnaik, R., Muresanu, D. F., Sahib, S., Tian, Z. R., … Sharma, H. S. (2021). **Upregulation of hemeoxygenase enzymes HO-1 and HO-2 following ischemia-reperfusion injury in connection with experimental cardiac arrest and cardiopulmonary resuscitation: Neuroprotective effects of methylene blue.** *Progress in Brain Research*, *265*, 317–375. https://doi.org/https://dx.doi.org/10.1016/bs.pbr.2021.06.009
Oxidative stress plays an important role in neuronal injuries after cardiac arrest. Increased production of carbon monoxide (CO) by the enzyme hemeoxygenase (HO) in the brain is induced by the oxidative stress. HO is present in the CNS in two isoforms, namely the inducible HO-1 and the constitutive HO-2. Elevated levels of serum HO-1 occurs in cardiac arrest patients and upregulation of HO-1 in cardiac arrest is seen in the neurons. However, the role of HO-2 in cardiac arrest is not well known. In this review involvement of HO-1 and HO-2 enzymes in the porcine brain following cardiac arrest and resuscitation is discussed based on our own observations. In addition, neuroprotective role of methylene blue- an antioxidant dye on alterations in HO under in cardiac arrest is also presented. The biochemical findings of HO-1 and HO-2 enzymes using ELISA were further confirmed by immunocytochemical approach to localize selective regional alterations in cardiac arrest. Our observations are the first to show that cardiac arrest followed by successful cardiopulmonary resuscitation results in significant alteration in cerebral concentrations of HO-1 and HO-2 levels indicating a prominent role of CO in brain pathology and methylene blue during CPR followed by induced hypothermia leading to superior neuroprotection after return of spontaneous circulation (ROSC), not reported earlier. Copyright © 2021 Elsevier B.V. All rights reserved.

38. Bogaerts, E., Ferdinande, B., Palmers, P. J., Malbrain, M. L. N. G., Van Regenmortel, N., Wilmer, A., … Ameloot, K. (2021). **The effect of fluid bolus administration on cerebral tissue oxygenation in post-cardiac arrest patients.** *Resuscitation*, *168*, 1–5. https://doi.org/https://dx.doi.org/10.1016/j.resuscitation.2021.08.044
PURPOSE: Fluid boluses (FB) are often used in post-cardiac arrest (CA) patients with haemodynamic instability. Although FB may improve cardiac output (CO) and mean arterial pressure (MAP), FB may also increase central venous pressure (CVP), reduce arterial PaO2, dilute haemoglobin and cause interstitial oedema. The aim of the present study was to investigate the net effect of FB administration on cerebral tissue oxygenation saturation (SctO2) in post-CA patients., METHODS: Pre-planned sub-study of the Neuroprotect post-CA trial (NCT02541591). Patients with anticipated fluid responsiveness based on stroke volume variation (SVV) or passive leg raising test were administered a FB of 500ml plasma-lyte A (Baxter Healthcare) and underwent pre- and post-FB assessments of stroke volume, CO, MAP, CVP, haemoglobin, PaO2 and SctO2., RESULTS: 52 patients (mean age 64+/-12years, 75% male) received a total of 115 FB. Although administration of a FB resulted in a significant increase of stroke volume (63+/-22 vs 67+/-23mL, p=0.001), CO (4,2+/-1,6 vs 4,4+/-1,7 L/min, p=0.001) and MAP (74,8+/-13,2 vs 79,2+/-12,9 mmHg, p=0.004), it did not improve SctO2 (68.54+/-6.99 vs 68.70+/-6.80%, p=0.49). Fluid bolus administration also resulted in a significant increase of CVP (10,0+/-4,5 vs 10,7+/-4,9 mmHg, p=0.02), but did not affect PaO2 (99+/-31 vs 94+/-31mmHg, p=0.15) or

haemoglobin concentrations (12,9+/-2,1 vs 12,8+/-2,2 g/dL, p=0.10). In a multivariate model, FB-induced changes in CO (beta 0,77; p=0.004) and in CVP (beta -0,23; p=0.02) but not in MAP (beta 0,02; p=0.18) predicted post-FB DELTASctO2., CONCLUSIONS: Despite improvements in CO and MAP, FB administration did not improve SctO2 in post-cardiac arrest patients. Copyright © 2021 Elsevier B.V. All rights reserved.

39. Annoni, F., Peluso, L., Gouvea Bogossian, E., Creteur, J., Zanier, E. R., & Taccone, F. S. (2021). **Brain Protection after Anoxic Brain Injury: Is Lactate Supplementation Helpful?.** *Cells*, *10*(7). https://doi.org/https://dx.doi.org/10.3390/cells10071714
While sudden loss of perfusion is responsible for ischemia, failure to supply the required amount of oxygen to the tissues is defined as hypoxia. Among several pathological conditions that can impair brain perfusion and oxygenation, cardiocirculatory arrest is characterized by a complete loss of perfusion to the brain, determining a whole brain ischemic-anoxic injury. Differently from other threatening situations of reduced cerebral perfusion, i.e., caused by increased intracranial pressure or circulatory shock, resuscitated patients after a cardiac arrest experience a sudden restoration of cerebral blood flow and are exposed to a massive reperfusion injury, which could significantly alter cellular metabolism. Current evidence suggests that cell populations in the central nervous system might use alternative metabolic pathways to glucose and that neurons may rely on a lactate-centered metabolism. Indeed, lactate does not require adenosine triphosphate (ATP) to be oxidated and it could therefore serve as an alternative substrate in condition of depleted energy reserves, i.e., reperfusion injury, even in presence of adequate tissue oxygen delivery. Lactate enriched solutions were studied in recent years in healthy subjects, acute heart failure, and severe traumatic brain injured patients, showing possible benefits that extend beyond the role as alternative energetic substrates. In this manuscript, we addressed some key aspects of the cellular metabolic derangements occurring after cerebral ischemia-reperfusion injury and examined the possible rationale for the administration of lactate enriched solutions in resuscitated patients after cardiac arrest.

40. Kupchik, N. (2021). **Targeted Temperature Management After Cardiac Arrest.** *Critical Care Nursing Clinics of North America*, *33*(3), 303–317. https://doi.org/https://dx.doi.org/10.1016/j.cnc.2021.05.006
and that reference citations are not used in the synopsis. A devastating complication of cardiac arrest is hypoxic-ischemic injury, which leads to neurologic dysfunction and subsequently high mortality. Post-cardiac arrest care is complex and requires a multimodal approach to manage hemodynamic instability as well as provide neuroprotection. Targeted temperature management is recommended by the American Heart Association as well as the International Liaison Committee on Resuscitation as a class 1 intervention for postarrest neuroprotection in patients who remain unresponsive after cardiac arrest. Copyright © 2021 Elsevier Inc. All rights reserved.

41. Shen, Y., Li, R., Yu, S., Zhao, Q., Wang, Z., Sheng, H., & Yang, W. (2021). **Activation of the ATF6 (Activating Transcription Factor 6) Signaling Pathway in Neurons Improves Outcome After Cardiac Arrest in Mice.** *Journal of the American Heart Association*, *10*(12), e020216. https://doi.org/https://dx.doi.org/10.1161/JAHA.120.020216
Background Ischemia/reperfusion injury impairs proteostasis, and triggers adaptive cellular responses, such as the unfolded protein response (UPR), which functions to restore endoplasmic reticulum homeostasis. After cardiac arrest (CA) and resuscitation, the UPR is activated in various organs including the brain. However, the role of the UPR in CA has remained largely unknown. Here we aimed to investigate effects of activation of the ATF6 (activating transcription factor 6) UPR branch in CA. Methods and Results Conditional and inducible sATF6-KI (short-form ATF6 knock-in) mice and a selective ATF6 pathway activator 147 were used. CA was induced in mice by KCl injection, followed by cardiopulmonary resuscitation. We first found that neurologic function was significantly improved, and neuronal damage was mitigated after the ATF6 pathway was activated in neurons of sATF6-KI mice subjected to CA/cardiopulmonary resuscitation. Further RNA sequencing analysis indicated that such beneficial effects were likely attributable to increased expression of pro-proteostatic genes regulated by ATF6. Especially, key components of the endoplasmic reticulum-associated degradation process, which clears potentially toxic unfolded/misfolded proteins in the endoplasmic reticulum, were upregulated in the sATF6-KI brain. Accordingly, the CA-induced increase in K48-linked polyubiquitin in the brain was higher in sATF6-KI mice relative to control mice. Finally, CA outcome, including the survival rate, was significantly improved in mice treated with compound 147. Conclusions This is the first experimental study to determine the role of the ATF6 UPR branch in CA outcome. Our data indicate that the ATF6 UPR branch is a prosurvival pathway and may be considered as a therapeutic target for CA.

42. Ruggeri, L., Nespoli, F., Ristagno, G., Fumagalli, F., Boccardo, A., Olivari, D., … Magliocca, A. (2021). **Esmolol during cardiopulmonary resuscitation reduces neurological injury in a porcine model of cardiac arrest.** *Scientific Reports*, *11*(1), 10635. https://doi.org/https://dx.doi.org/10.1038/s41598-021-90202-w
Primary vasopressor efficacy of epinephrine during cardiopulmonary resuscitation (CPR) is due to its alpha-adrenergic effects. However, epinephrine plays beta1-adrenergic actions, which increasing myocardial oxygen consumption may lead to refractory ventricular fibrillation (VF) and poor outcome. Effects of a single dose of esmolol in addition to epinephrine during CPR were investigated in a porcine model of VF with an underlying acute myocardial infarction. VF was ischemically induced in 16 pigs and left untreated for 12 min. During CPR, animals were randomized to receive epinephrine (30 microg/kg) with either esmolol (0.5 mg/kg) or saline (control). Pigs were then observed up to 96 h.

Coronary perfusion pressure increased during CPR in the esmolol group compared to control (47 +/- 21 vs. 24 +/- 10 mmHg at min 5, p < 0.05). In both groups, 7 animals were successfully resuscitated and 4 survived up to 96 h. No significant differences were observed between groups in the total number of defibrillations delivered prior to final resuscitation. Brain histology demonstrated reductions in cortical neuronal degeneration/necrosis (score 0.3 +/- 0.5 vs. 1.3 +/- 0.5, p < 0.05) and hippocampal microglial activation (6 +/- 3 vs. 22 +/- 4%, p < 0.01) in the esmolol group compared to control. Lower circulating levels of neuron specific enolase were measured in esmolol animals compared to controls (2[1-3] vs. 21[16-52] ng/mL, p < 0.01). In this preclinical model, beta1-blockade during CPR did not facilitate VF termination but provided neuroprotection.

43. Sedky, A. A., & Magdy, Y. (2021). **Reduction in TNF alpha and oxidative stress by liraglutide: Impact on ketamine-induced cognitive dysfunction and hyperlocomotion in rats.** *Life Sciences*, *278*, 119523. https://doi.org/https://dx.doi.org/10.1016/j.lfs.2021.119523
BACKGROUND: Diabetes and psychotic disorders are occasionally comorbid. Possible pathophysiologies linking these disorders include inflammation and oxidative stress. Glucagon like peptide-1 (GLP-1) agonists modulate glucose metabolism and may exert neuroprotective effects via central GLP-1 receptors., AIM OF THE WORK: To explore the effects of GLP-1 agonist, liraglutide, on ketamine-induced hyper-locomotion and cognitive dysfunction and the associated inflammation and oxidative stress in normoglycemic and diabetic rats., METHODS: Rats were divided into: Chow fed (non-diabetic) and high fat diet fed/STZ (diabetic) groups: I. non-diabetic/control, non-diabetic/liraglutide, non-diabetic/ketamine, non-diabetic/ketamine/liraglutide groups. II. diabetic/control, diabetic/liraglutide, diabetic/ketamine and diabetic/ketamine/liraglutide groups. Hyperlocomotion and cognitive dysfunction were assessed using open field and water maze tests. Biochemical parameters were measured in serum and hippocampus., RESULTS: Ketamine induced hyperlocomotion and cognitive dysfunction, with hippocampal histopathological changes. Increase in tumour necrosis factor (TNF)-alpha and oxidative stress and reduction in brain-derived neurotrophic factor (BDNF) were noted. These changes were augmented in diabetic compared to non-diabetic rats. Liraglutide significantly improved hyperlocomotion, and cognitive dysfunction and hippocampal histopathological changes in non-diabetic and diabetic rats. Improvement in glucose homeostasis, reduction in TNF alpha and malondialdehyde, and increase in glutathione and BDNF were observed in serum and hippocampus., CONCLUSION: Beneficial effects of liraglutide on ketamine-induced hyperlocomotion and cognitive dysfunction are associated with reduction in TNF alpha and oxidative stress. Since effects of liraglutide occurred in diabetic and non-diabetic rats, glycemic and non-glycemic effects (via central GLP-1 receptors) might be involved. Targeting oxidative stress and inflammation by GLP-1 agonists, may be a promising approach in psychotic patients with diabetes. Copyright © 2021 Elsevier Inc. All rights reserved.

44. Morgan, R. W., Sutton, R. M., Himebauch, A. S., Roberts, A. L., Landis, W. P., Lin, Y., … Kilbaugh, T. J. (2021). **A randomized and blinded trial of inhaled nitric oxide in a piglet model of pediatric cardiopulmonary resuscitation.** *Resuscitation*, *162*, 274–283. https://doi.org/https://dx.doi.org/10.1016/j.resuscitation.2021.03.004
AIM: Inhaled nitric oxide (iNO) during cardiopulmonary resuscitation (CPR) improved systemic hemodynamics and outcomes in a preclinical model of adult in-hospital cardiac arrest (IHCA) and may also have a neuroprotective role following cardiac arrest. The primary objectives of this study were to determine if iNO during CPR would improve cerebral hemodynamics and mitochondrial function in a pediatric model of lipopolysaccharide-induced shock-associated IHCA., METHODS: After lipopolysaccharide infusion and ventricular fibrillation induction, 20 1-month-old piglets received hemodynamic-directed CPR and were randomized to blinded treatment with or without iNO (80ppm) during and after CPR. Defibrillation attempts began at 10min with a 20-min maximum CPR duration. Cerebral tissue from animals surviving 1-h post-arrest underwent high-resolution respirometry to evaluate the mitochondrial electron transport system and immunohistochemical analyses to assess neuropathology., RESULTS: During CPR, the iNO group had higher mean aortic pressure (41.6+/-2.0 vs. 36.0+/-1.4mmHg; p=0.005); diastolic BP (32.4+/-2.4 vs. 27.1+/-1.7mmHg; p=0.03); cerebral perfusion pressure (25.0+/-2.6 vs. 19.1+/-1.8mmHg; p=0.02); and cerebral blood flow relative to baseline (rCBF: 243.2+/-54.1 vs. 115.5+/-37.2%; p=0.02). Among the 8/10 survivors in each group, the iNO group had higher mitochondrial Complex I oxidative phosphorylation in the cerebral cortex (3.60 [3.56, 3.99] vs. 3.23 [2.44, 3.46] pmol O2/smg; p=0.01) and hippocampus (4.79 [4.35, 5.18] vs. 3.17 [2.75, 4.58] pmol O2/smg; p=0.02). There were no other differences in mitochondrial respiration or brain injury between groups., CONCLUSIONS: Treatment with iNO during CPR resulted in superior systemic hemodynamics, rCBF, and cerebral mitochondrial Complex I respiration in this pediatric cardiac arrest model. Copyright © 2021 Elsevier B.V. All rights reserved.

45. Wang, L., Deng, B., Yan, P., Wu, H., Li, C., Zhu, H., … Hou, L. (2021). **Neuroprotective effect of ketamine against TNF-alpha-induced necroptosis in hippocampal neurons.** *Journal of Cellular and Molecular Medicine*, *25*(7), 3449–3459. https://doi.org/https://dx.doi.org/10.1111/jcmm.16426
Tumour necrosis factor-alpha (TNF-alpha), a crucial cytokine, has various homeostatic and pathogenic bioactivities. The aim of this study was to assess the neuroprotective effect of ketamine against TNF-alpha-induced motor dysfunction and neuronal necroptosis in male C57BL/6J mice in vivo and HT-22 cell lines in vitro. The behavioural testing results of the present study indicate that ketamine ameliorated TNF-alpha-induced neurological dysfunction. Moreover, immunohistochemical staining results showed that TNF-alpha-induced brain dysfunction was caused by necroptosis and microglial activation, which could be attenuated by ketamine pre-treatment inhibiting reactive oxygen species production and mixed lineage kinase domain-like phosphorylation in hippocampal neurons. Therefore, we concluded that ketamine

may have neuroprotective effects as a potent inhibitor of necroptosis, which provides a new theoretical and experimental basis for the application of ketamine in TNF-alpha-induced necroptosis-associated diseases. Copyright © 2021 The Authors. Journal of Cellular and Molecular Medicine published by Foundation for Cellular and Molecular Medicine and John Wiley & Sons Ltd.

46. Ghanavatian, S., James, D. L., & Sadolf, J. S. (2021). **The role of short-term, low dose intravenous ketamine infusion in Calciphylaxis.** *CEN Case Reports*, *10*(3), 422–425. https://doi.org/https://dx.doi.org/10.1007/s13730-020-00557-8
Calciphylaxis is a small vessel vasculopathy causing subcutaneous ischemic necrosis. This condition is a recognized complication of end stage renal disease and is associated with severe pain. The mechanism of the pain generated by calciphylaxis is thought to be partly related to tissue ischemia, with a significant neuropathic component associated with neuronal hypoxic injury. The pain can be further exacerbated by the inflammatory process ensuing as a result of calciphylactic lesion infections which are commonly associated with this condition. Obtaining adequate pain relief is a challenging aspect of symptom control in calciphylaxis, and historically, patients suffering from calciphylaxis required high dose opioid medications to achieve satisfactory analgesia. This case report presents a multimodal pain management approach utilizing low dose ketamine infusion in an opioid-tolerant patient suffering from severe calciphylaxis-related pain. Ketamine is an anesthetic agent well established for its efficacy in the management of neuropathic pain in opioid-tolerant patients, and has been shown to prevent opioid-induced hyperalgesia and decrease opioid requirements. Prior published data studying pain control in calciphylaxis have mainly focused on subcutaneous ketamine administration which as noted in the literature, can be associated with infusion site complications. To the best of our knowledge, this report is first of its kind to describe successful use of ketamine infusion in treatment of acute calciphylaxis-related pain.Dose modification of ketamine is not required for patients with impaired renal function, and low dose intravenous ketamine infusion was associated with no reported adverse effects in our patient. Copyright © 2021. Japanese Society of Nephrology.

47. Wu, C. Y.-C., Couto E Silva, A., Citadin, C. T., Clemons, G. A., Acosta, C. H., Knox, B. A., … Lin, H. W. (2021). **Palmitic acid methyl ester inhibits cardiac arrest-induced neuroinflammation and mitochondrial dysfunction.** *Prostaglandins, Leukotrienes, and Essential Fatty Acids*, *165*, 102227. https://doi.org/https://dx.doi.org/10.1016/j.plefa.2020.102227
We previously discovered that palmitic acid methyl ester (PAME) is a potent vasodilator released from the sympathetic ganglion with vasoactive properties. Post-treatment with PAME can enhance cortical cerebral blood flow and functional learning and memory, while inhibiting neuronal cell death in the CA1 region of the hippocampus under pathological conditions (i.e. cerebral ischemia). Since mechanisms underlying PAME-mediated neuroprotection remain unclear, we investigated the possible neuroprotective mechanisms of PAME after 6 min of asphyxial cardiac arrest (ACA, an animal model of global cerebral ischemia). Our results from capillary-based immunoassay (for the detection of proteins) and cytokine array suggest that PAME (0.02 mg/kg) can decrease neuroinflammatory markers, such as ionized calcium binding adaptor molecule 1 (Iba1, a specific marker for microglia/macrophage activation) and inflammatory cytokines after cardiopulmonary resuscitation. Additionally, the mitochondrial oxygen consumption rate (OCR) and respiratory function in the hippocampal slices were restored following ACA (via Seahorse XF24 Extracellular Flux Analyzer) suggesting that PAME can ameliorate mitochondrial dysfunction. Finally, hippocampal protein arginine methyltransferase 1 (PRMT1) and PRMT8 are enhanced in the presence of PAME to suggest a possible pathway of methylated fatty acids to modulate arginine-based enzymatic methylation. Altogether, our findings suggest that PAME can provide neuroprotection in the presence of ACA to alleviate neuroinflammation and ameliorate mitochondrial dysfunction. Copyright © 2020. Published by Elsevier Ltd.

48. Wang, W.-Y., Xie, L., Zou, X.-S., Li, N., Yang, Y.-G., Wu, Z.-J., … Chen, M.-H. (2021). **Inhibition of extracellular signal-regulated kinase/calpain-2 pathway reduces neuroinflammation and necroptosis after cerebral ischemia-reperfusion injury in a rat model of cardiac arrest.** *International Immunopharmacology*, *93*, 107377. https://doi.org/https://dx.doi.org/10.1016/j.intimp.2021.107377
BACKGROUND: Cerebral ischemia-reperfusion injury (CIRI) is the leading cause of poor neurological prognosis after cardiopulmonary resuscitation (CPR). We previously reported that the extracellular signal-regulated kinase (ERK) activation mediates CIRI. Here, we explored the potential ERK/calpain-2 pathway role in CIRI using a rat model of cardiac arrest (CA)., METHODS: Adult male Sprague-Dawley rats suffered from CA/CPR-induced CIRI, received saline, DMSO, PD98059 (ERK1/2 inhibitor, 0.3 mg/kg), or MDL28170 (calpain inhibitor, 3.0 mg/kg) after spontaneous circulation recovery. The survival rate and the neurological deficit score (NDS) were utilized to assess the brain function. Hematoxylin stain, Nissl staining, and transmission electron microscopy were used to evaluate the neuron injury. The expression levels of p-ERK, ERK, calpain-2, neuroinflammation-related markers (GFAP, Iba1, IL-1beta, TNF-alpha), and necroptosis proteins (TNFR1, RIPK1, RIPK3, p-MLKL, and MLKL) in the brain tissues were determined by western blotting and immunohistochemistry. Fluorescent multiplex immunohistochemistry was used to analyze the p-ERK, calpain-2, and RIPK3 co-expression in neurons, and RIPK3 expression levels in microglia or astrocytes., RESULTS: At 24 h after CA/CPR, the rats in the saline-treated and DMSO groups presented with injury tissue morphology, low NDS, ERK/calpain-2 pathway activation, and inflammatory cytokine and necroptosis protein over-expression in the brain tissue. After PD98059 and MDL28170 treatment, the brain function was improved, while inflammatory response and

necroptosis were suppressed by ERK/calpain-2 pathway inhibition., CONCLUSION: Inflammation activation and necroptosis involved in CA/CPR-induced CIRI were regulated by the ERK/calpain-2 signaling pathway. Inhibition of that pathway can reduce neuroinflammation and necroptosis after CIRI in the CA model rats. Copyright © 2021. Published by Elsevier B.V.

49. Li, F., Zhang, J., Chen, A., Liao, R., Duan, Y., Xu, Y., & Tao, L. (2021). **Combined transplantation of neural stem cells and bone marrow mesenchymal stem cells promotes neuronal cell survival to alleviate brain damage after cardiac arrest via microRNA-133b incorporated in extracellular vesicles.** *Aging*, *13*(1), 262–278. https://doi.org/https://dx.doi.org/10.18632/aging.103920
Neural stem cell (NSC) transplantation has prevailed as a promising protective strategy for cardiac arrest (CA)-induced brain damage. Surprisingly, the poor survival of neuronal cells in severe hypoxic condition restricts the utilization of this cell-based therapy. Extracellular vesicles (EVs) transfer microRNAs (miRNAs) between cells are validated as the mode for the release of several therapeutic molecules. The current study reports that the bone marrow mesenchymal stem cells (BMSCs) interact with NSCs via EVs thereby affecting the survival of neuronal cells. Hypoxic injury models of neuronal cells were established using cobalt chloride, followed by co-culture with BMSCs and NSCs alone or in combination. BMSCs combined with NSCs elicited as a superior protocol to stimulate neuronal cell survival. BMSCs-derived EVs could protect neuronal cells against hypoxic injury. Silencing of miR-133b incorporated in BMSCs-derived EVs could decrease the cell viability and the number of NeuN-positive cells and increase the apoptosis in the CA rat model. BMSCs-derived EVs could transfer miR-133b to neuronal cells to activate the AKT-GSK-3beta-WNT-3 signaling pathway by targeting JAK1. Our study demonstrates that NSCs promotes the release of miR-133b from BMSCs-derived EVs to promote neuronal cell survival, representing a potential therapeutic strategy for the treatment of CA-induced brain damage.

50. Patel, W., Rimmer, L., Smith, M., Moss, L., Smith, M. A., Snodgrass, H. R., … Dickens, D. (2021). **Probenecid Increases the Concentration of 7-Chlorokynurenic Acid Derived from the Prodrug 4-Chlorokynurenine within the Prefrontal Cortex.** *Molecular Pharmaceutics*, *18*(1), 113–123. https://doi.org/https://dx.doi.org/10.1021/acs.molpharmaceut.0c00727
Recent advances in the understanding of depression have led to increasing interest in ketamine and the role that N-methyl-d-aspartate (NMDA) receptor inhibition plays in depression. l-4-Chlorokynurenine (4-Cl-KYN, AV-101), a prodrug, has shown promise as an antidepressant in preclinical studies, but this promise has not been realized in recent clinical trials. We sought to determine if transporters in the CNS could be playing a role in this clinical response. We used radiolabeled uptake assays and microdialysis studies to determine how 4-Cl-KYN and its active metabolite, 7-chlorokynurenic acid (7-Cl-KYNA), cross the blood-brain barrier (BBB) to access the brain and its extracellular fluid compartment. Our data indicates that 4-Cl-KYN crosses the blood-brain barrier via the amino acid transporter LAT1 (SLC7A5) after which the 7-Cl-KYNA metabolite leaves the brain extracellular fluid via probenecid-sensitive organic anion transporters OAT1/3 (SLC22A6 and SLC22A8) and MRP4 (ABCC4). Microdialysis studies further validated our in vitro data, indicating that probenecid may be used to boost the bioavailability of 7-Cl-KYNA. Indeed, we found that coadministration of 4-Cl-KYN with probenecid caused a dose-dependent increase by as much as an 885-fold increase in 7-Cl-KYNA concentration in the prefrontal cortex. In summary, our data show that 4-Cl-KYN crosses the BBB using LAT1, while its active metabolite, 7-Cl-KYNA, is rapidly transported out of the brain via OAT1/3 and MRP4. We also identify a hitherto unreported mechanism by which the brain extracellular concentration of 7-Cl-KYNA may be increased to produce significant boosting of the drug concentration at its site of action that could potentially lead to an increased therapeutic effect.

51. Fujikawa, R., Yamada, J., & Jinno, S. (2021). **Subclass imbalance of parvalbumin-expressing GABAergic neurons in the hippocampus of a mouse ketamine model for schizophrenia, with reference to perineuronal nets.** *Schizophrenia Research*, *229*, 80–93. https://doi.org/https://dx.doi.org/10.1016/j.schres.2020.11.016
Impairments of parvalbumin-expressing GABAergic neurons (PV+ neurons) and specialized extracellular structures called perineuronal nets (PNNs) have been found in schizophrenic patients. In this study, we examined potential alterations in four subclasses of PV+ neurons colocalized with PNNs in the hippocampus of a mouse ketamine model for schizophrenia. Because biosynthesis of human natural killer-1 (HNK-1) is shown to be associated with the risk of schizophrenia, here we used mouse monoclonal Cat-315 antibody, which recognizes HNK-1 glycans on PNNs. Once-daily intraperitoneal injections of ketamine for seven consecutive days induced hyper-locomotor activity in the open field tests. The prepulse inhibition (PPI) test showed that PPI scores declined in ketamine-treated mice compared to vehicle-treated mice. The densities of PV+ neurons and Cat-315+ PNNs declined in the CA1 region of ketamine-treated mice. Interestingly, the density of Cat-315+/PV+ neurons was lower in ketamine-treated mice than in vehicle-treated mice, whereas the density of Cat-315-/PV+ neurons was not affected by ketamine. Among the four subclasses of PV+ neurons, the densities of Cat-315+/PV+ basket cells and Cat-315-/PV+ axo-axonic cells were lower in ketamine-treated mice than in vehicle-treated mice, while the densities of Cat-315-/PV+ basket cells and Cat-315+/PV+ axo-axonic cells were not affected by ketamine. Taken together, PNNs may not play a simple neuroprotective role against ketamine. Because different subclasses of PV+ neurons are considered to play distinct roles in the hippocampal neuronal network, the ketamine-induced subclass imbalance of PV+ neurons may result in abnormal network activity, which underlies the pathophysiology of schizophrenia. Copyright © 2020 Elsevier B.V. All rights reserved.

52. Keilhoff, G., Titze, M., Rathert, H., Nguyen Thi, T. M., & Ebmeyer, U. (2021). **The Spinal Cord Damage in a Rat Asphyxial Cardiac Arrest/Resuscitation Model.** *Neurocritical Care*, *34*(3), 844–855. https://doi.org/https://dx.doi.org/10.1007/s12028-020-01094-z
BACKGROUND: After cardiac arrest/resuscitation (CA/R), animals often had massive functional restrictions including spastic paralysis of hind legs, disturbed balance and reflex abnormalities. Patients who have survived CA also develop movement restrictions/disorders. A successful therapy requires detailed knowledge of the intrinsic damage pattern and the respective mechanisms. Beside neurodegenerations in the cerebellum and cortex, neuronal loss in the spinal cord could be a further origin of such movement artifacts., METHODS: Thus, we aimed to evaluate the CA/R-induced degeneration pattern of the lumbar medulla spinalis by immunocytochemical expression of SMI 311 (marker of neuronal perikarya and dendrites), IBA1 (microglia marker), GFAP (marker of astroglia), calbindin D28k (marker of the cellular neuroprotective calcium-buffering system), MnSOD (neuroprotective antioxidant), the transcription factor PPARgamma and the mitochondrial marker protein PDH after survival times of 7 and 21 days. The CA/R specimens were compared with those from sham-operated and completely naive rats. RESULTS & CONCLUSION: The main ACA/R-mediated results were: (1) degeneration of lumbar spinal cord motor neurons, characterized by neuronal pyknotization and peri-neuronal tissue artifacts; (2) attendant activation of microglia in the short-term group; (3) attendant activation of astroglia in the long-term group; (4) degenerative pattern in the intermediate gray matter; (5) activation of the endogenous anti-oxidative defense systems calbindin D28k and MnSOD; (6) activation of the transcription factor PPARgamma, especially in glial cells of the gray matter penumbra; and (7) activation of mitochondria. Moreover, marginal signs of anesthesia-induced cell stress were already evident in sham animals when compared with completely naive spinal cords. A correlation between the NDS and the motor neuronal loss could not be verified. Thus, the NDS appears to be unsuitable as prognostic tool.

53. Zhang, B., Gu, Q., Chen, X., You, Y., Chen, M., Qian, Y., … Yu, W. (2021). **Temperature Variability Does Not Attenuate the Beneficial Effects of Therapeutic Hypothermia on Cellular Apoptosis and Endoplasmic Reticulum Stress in the Cerebral Cortex of a Swine Cardiac Arrest Model.** *Neurocritical Care*, *34*(3), 769–780. https://doi.org/https://dx.doi.org/10.1007/s12028-020-01083-2
BACKGROUND: Endoplasmic reticulum stress (ERS) plays a vital role in mediating apoptosis in the brain following cardiac arrest (CA). Studies have shown that therapeutic hypothermia (TH) provides neuroprotection through anti-apoptosis; however, the effects of temperature variability in TH on the brain remain unclear. In this study, we investigated the different effects of temperature variability through extracorporeal membrane oxygenation on apoptosis and ERS in the brain following CA., METHODS: Eighteen male domestic pigs underwent 6-min duration of no-flow induced by ventricular fibrillation. Extracorporeal cardiopulmonary resuscitation was then performed, and the return of spontaneous circulation (ROSC) was achieved. The animals were randomly assigned to the following groups: normothermia, non-temperature variability, and temperature variability. TH (core temperature, 33-35 degreeC) was maintained for 24 h post-ROSC, and the animals were rewarmed for 8 h. Quantitative real-time polymerase chain reaction (qRT-PCR) and immunohistochemistry for Bax and Bcl-2 transcripts and proteins, respectively, were used to investigate apoptosis in the cerebral cortex. Expression levels of the ERS molecules, GRP78 and CHOP, were also detected by qRT-PCR, and cellular morphology was evaluated using transmission electron microscopy., RESULTS: qRT-PCR and immunohistochemistry results revealed that TH significantly increased the expression levels of Bcl-2 and GRP78 and decreased that of Bax and CHOP than under normothermia conditions. Compared to the non-temperature variability group, temperature variability did not decrease the expression levels of Bcl-2 and GRP78 and not increase the levels of Bax and CHOP. Endoplasmic reticulum ultrastructural changes were significantly improved under TH. No statistical difference was observed between the temperature variability and non-temperature variability groups., CONCLUSION: TH can reduce neuronal apoptosis by ERS, while temperature variability does not attenuate this beneficial effect.

54. Li, Q., & Zhang, X. (2021). **Genistein attenuates cognitive deficits and neuroapoptosis in hippocampus induced by ketamine exposure in neonatal rats.** *Synapse (New York, N.Y.)*, *75*(1), e22181. https://doi.org/https://dx.doi.org/10.1002/syn.22181
Ketamine is a frequently used anesthetic in pediatric patients that can cause cognitive impairment. Genistein, a bioactive component of soy products, has been shown to suppress neuronal death through regulating the expression of apoptosis related genes. In this study, we hypothesized that genistein could alleviate ketamine-induced cognitive impairment by ameliorating hippocampal neuronal loss and tested this hypothesis in rats. Neonatal rats were treated with ketamine and genistein. Hippocampal tissue was harvested for histological and biochemical analysis to determine neuronal apoptosis and proteins involved in the apoptotic pathways. Behavioral assays including contextual fear conditioning test and Morris water maze test were performed to assess cognitive functions, including learning and memory. We found that in fear conditioning test, genistein restored freezing time in ketamine treated rats in a dose dependent manner. Similarly, genistein attenuated impaired learning and memory in Morris water maze test in rats treated with ketamine. Additionally, ketamine-induced neuronal apoptosis in rat hippocampus was attenuated by genistein treatment. Finally, we found that genistein partially restored proteins associated with apoptosis, including Bax, Bcl-2, cleaved caspase 3, and phosphorylated GSK-3s and Akt. Genistein suppresses hippocampal neuronal loss and cognitive disruption induced by ketamine in rats. Copyright © 2020 Wiley Periodicals LLC.

55. Venkataramaiah, C., Lakshmi Priya, B., & Rajendra, W. (2021). **Perturbations in the catecholamine**

metabolism and protective effect of "3-(3, 4-dimethoxy phenyl)-1-4(methoxy phenyl) prop-2-en-1-one" during ketamine-induced schizophrenia: an in vivo and in silico studies. *Journal of Biomolecular Structure & Dynamics*, *39*(10), 3523–3532. https://doi.org/https://dx.doi.org/10.1080/07391102.2020.1765875
Different kinds of secondary metabolites present in the medicinal plants play an important role to alleviate different human ailments including neurodegenerative disorders such as Parkinson's, Alzheimer's, epilepsy and schizophrenia etc. Recently we have isolated and characterized a novel bioactive compound viz. 3-(3,4-dimethoxy phenyl)-1-4(methoxy phenyl)prop-2-en-1-one from the methanolic extract of Celastrus paniculatus (CP) which has been widely used for the treatment of neurodegenerative diseases. The present investigation is mainly aimed to evaluate the neuroprotective potential of the above bioactive compound against ketamine-induced schizophrenia with particular reference to catecholaminergic metabolism using in vivo and in silico methods. Ketamine-induced schizophrenia caused significant elevation in biogenic amines (epinephrine, nor epinephrine, dopamine and 5-HT) and monoamine oxidase activity levels which were restored to normal during the treatment with the bioactive compound akin to the reference compound, clozapine. In addition, the compound has shown highest binding score against all the biogenic amine receptors viz. D1, D2, D3, D4 and serotonin receptor, 5-HT2A with lowest inhibition constant values than the reference compound, clozapine. The present findings suggest that modulation of CNS monoamine neurotransmitter system might partly contribute to the impairments associated with schizophrenia and the plant compound alleviates the monoaminergic abnormalities associated with the neurological dysfunction.Communicated by Ramaswamy H. Sarma.

56. Occhieppo, V. B., Basmadjian, O. M., Marchese, N. A., Silvero C, M. J., Rodriguez, A., Armonelli, S., … Bregonzio, C. (2021). **AT1 -R is involved in the development of long-lasting, region-dependent and oxidative stress-independent astrocyte morphological alterations induced by Ketamine.** *The European Journal of Neuroscience*, *54*(5), 5705–5716. https://doi.org/https://dx.doi.org/10.1111/ejn.14756
Astrocytes play an essential role in the genesis, maturation and regulation of the neurovascular unit. Multiple evidence support that astrocyte reactivity has a close relationship to neurovascular unit dysfunction, oxidative stress and inflammation, providing a suitable scenario for the development of mental disorders. Ketamine has been proposed as a single-use antidepressant treatment in major depression, and its antidepressant effects have been associated with anti-inflammatory properties. However, Ketamine long-lasting effects over the neurovascular unit components remain unclear. Angiotensin II AT1 receptor (AT1 -R) blockers have anti-inflammatory, antioxidant and neuroprotective effects. The present work aims to distinguish the acute and long-term Ketamine effects over astrocytes response extended to other neurovascular unit components, and the involvement of AT1 -R, in prefrontal cortex and ventral tegmental area. Male Wistar rats were administered with AT1 -R antagonist Candesartan/Vehicle (days 1-10) and Ketamine/Saline (days 6-10). After 14 days drug-free, at basal conditions or after Ketamine Challenge, the brains were processed for oxidative stress analysis, cresyl violet staining and immunohistochemistry for glial, neuronal activation and vascular markers. Repeated Ketamine administration induced long-lasting region-dependent astrocyte reactivity and morphological alterations, and neuroadaptative changes observed as exacerbated oxidative stress and neuronal activation, prevented by the AT1 -R blockade. Ketamine Challenge decreased microglial and astrocyte reactivity and augmented cellular apoptosis, independently of previous treatment. Overall, AT1 -R is involved in the development of neuroadaptative changes induced by repeated Ketamine administration but does not interfere with the acute effects supporting the potential use of AT1 -R blockers as a Ketamine complementary therapy in mental disorders. Copyright © 2020 Federation of European Neuroscience Societies and John Wiley & Sons Ltd.

57. C.C., G., B.R., G., J.C.S., G., J.C., M., M.M., T., Blanque R R, … Rodriguez Blanque, R. (2021). **The effect of therapeutic hypothermia after cardiac arrest on the neurological outcome and survival-a systematic review of rcts published between 2016 and 2020**. *International Journal of Environmental Research and Public Health*, *18*(22), 11817. https://doi.org/https://dx.doi.org/10.3390/ijerph182211817
Therapeutic hypothermia is a treatment used for patients who have suffered cardiorespiratory arrest and remain conscious after the recovery of spontaneous circulation. However, its effectiveness is controversial. The objective of this systematic review is to summarize the scientific evidence available about the effect of therapeutic hypothermia on neurological status and survival in this type of patients., METHODOLOGY: A primary search in CINAHL, CUIDEN, Pubmed, Web of Science, and Scopus databases was carried out. Randomized clinical trials (RCT) published from 2016 to 2020 were selected., RESULTS: 17 studies were selected for inclusion and most relevant data were extracted. Methodological quality was assessed by the RoB tool., CONCLUSIONS: Although therapeutic hypothermia is a safe technique with few adverse and manageable effects, it has not shown to improve survival rate and neurological status of adult nor pediatric patients. It is possible that its positive effect on neuroprotection could be achieved only by preventing hyperthermia although further investigation is needed.

58. Dai, C., Wang, J. J., Li, J., Wang, J. J., Zhang, L., Yin, C., … Dai C Li J, Wang J, Zhang L, Yin C, Li Y, W. J. (2021). **Repetitive anodal transcranial direct current stimulation improves neurological recovery by preserving the neuroplasticity in an asphyxial rat model of cardiac arrest.** *Brain Stimulation*, *14*(2), 407. https://doi.org/https://dx.doi.org/10.1016/j.brs.2021.02.008
BACKGROUND: Non-shockable rhythms present an increasing proportion of out-of-hospital cardiac arrest (CA) patients, but are associated with poor prognosis and received limited therapeutic effect of targeted temperature management (TTM). Previous study showed repetitive anodal transcranial direct current stimulation (tDCS) improved neurological

outcomes in animals with ventricular fibrillation. Here, we examine the effectiveness of tDCS on neurological recovery and the potential mechanisms in a rat model of asphyxial CA., METHOD: Cardiopulmonary resuscitation was initiated after 5 min of untreated asphyxial CA. Animals were randomized to three experimental groups immediately after successful resuscitation (n = 12/group, 6 males): no-treatment control (NTC) group, TTM group, and tDCS group. Post resuscitation hemodynamics, quantitative electroencephalogram (EEG), neurological deficit score, and 96-h survival were evaluated. Brain tissues of additional animals undergoing same experimental procedure was harvested for enzyme-linked immunoassay-based quantification assays of neuroplasticity-related biomarkers and compared with the sham-operated rats (n = 6/group)., RESULTS: We observed that after resuscitation tDCS-treated animals exhibited significantly higher mean arterial pressure and left ventricular ejection fraction than NTC group and showed greatly improved EEG characteristics including weighted-permutation entropy and gamma band power, and neurologic deficit scores and 96-h survival rates compared to NTC and TTM groups. Furthermore, neuroplastic biomarkers including microtubule-associated protein 2, growth-associated protein 43, postsynaptic density protein 95 and synaptophysin, were significantly higher in tDCS group when compared with NTC and TTM groups., CONCLUSION: In this rat model of asphyxial CA, repetitive anodal tDCS commenced after resuscitation improved neurological recovery, and it may exert a neuroprotective effect by preserving the neuroplasticity. Copyright © 2021 The Author(s). Published by Elsevier Inc. All rights reserved.

59. Andersen LW Kjaergaard J, Kristensen CM, Darling S, Zwisler ST, Fisker S, Schmidt JC, Kirkegaard H, Grejs AM, Rossau JRG, Larsen JM, Rasmussen BS, Riddersholm S, Iversen K, Schultz M, Nielsen JL, Lofgren B, Lauridsen KG, Solling C, Paelestik K, Kjaergaard AG, Due-Rasmussen D, Folke F, Charlot MG, Jepsen RMHG, Wiberg S, Donnino M, Kurth T, Hoybye M, Sindberg B, Holmberg MJ, Granfeldt A, I. D., Andersen, L. W., Isbye, D., Kjaergaard, J., Kristensen, C. M., Darling, S., … Granfeldt, A. (2021). **Effect of Vasopressin and Methylprednisolone vs Placebo on Return of Spontaneous Circulation in Patients With In-Hospital Cardiac Arrest: a Randomized Clinical Trial**. *JAMA*, *326*(16), 1586–1594. https://doi.org/https://dx.doi.org/10.1001/jama.2021.16628
Importance: Previous trials have suggested that vasopressin and methylprednisolone administered during in-hospital cardiac arrest might improve outcomes., Objective: To determine whether the combination of vasopressin and methylprednisolone administered during in-hospital cardiac arrest improves return of spontaneous circulation., Design, Setting, and Participants: Multicenter, randomized, double-blind, placebo-controlled trial conducted at 10 hospitals in Denmark. A total of 512 adult patients with in-hospital cardiac arrest were included between October 15, 2018, and January 21, 2021. The last 90-day follow-up was on April 21, 2021., Intervention: Patients were randomized to receive a combination of vasopressin and methylprednisolone (n = 245) or placebo (n = 267). The first dose of vasopressin (20 IU) and methylprednisolone (40 mg), or corresponding placebo, was administered after the first dose of epinephrine. Additional doses of vasopressin or corresponding placebo were administered after each additional dose of epinephrine for a maximum of 4 doses., Main Outcomes and Measures: The primary outcome was return of spontaneous circulation. Secondary outcomes included survival and favorable neurologic outcome at 30 days (Cerebral Performance Category score of 1 or 2)., Results: Among 512 patients who were randomized, 501 met all inclusion and no exclusion criteria and were included in the analysis (mean [SD] age, 71 [13] years; 322 men [64%]). One hundred of 237 patients (42%) in the vasopressin and methylprednisolone group and 86 of 264 patients (33%) in the placebo group achieved return of spontaneous circulation (risk ratio, 1.30 [95% CI, 1.03-1.63]; risk difference, 9.6% [95% CI, 1.1%-18.0%]; P = .03). At 30 days, 23 patients (9.7%) in the intervention group and 31 patients (12%) in the placebo group were alive (risk ratio, 0.83 [95% CI, 0.50-1.37]; risk difference: -2.0% [95% CI, -7.5% to 3.5%]; P = .48). A favorable neurologic outcome was observed in 18 patients (7.6%) in the intervention group and 20 patients (7.6%) in the placebo group at 30 days (risk ratio, 1.00 [95% CI, 0.55-1.83]; risk difference, 0.0% [95% CI, -4.7% to 4.9%]; P > .99). In patients with return of spontaneous circulation, hyperglycemia occurred in 77 (77%) in the intervention group and 63 (73%) in the placebo group. Hypernatremia occurred in 28 (28%) and 27 (31%), in the intervention and placebo groups, respectively., Conclusions and Relevance: Among patients with in-hospital…

60. Wang, W., Xie, L., Zou, X., Hu, W., Tian, X., Zhao, G., … Chen M. (2021). **Pomelo peel oil suppresses TNF-alpha-induced necroptosis and cerebral ischaemia-reperfusion injury in a rat model of cardiac arrest.** *Pharmaceutical Biology*, *59*(1), 401–409. https://doi.org/https://dx.doi.org/10.1080/13880209.2021.1903046
Context: Pomelo peel oil (PPO) [Citrus maxima (Burm.) Merr. (Rutaceae)] is reported to possess antioxidant and antimelanogenic activities. Objective(s): To investigate the effect of PPO [Citrus maxima (Burm.) Merr. cv. Shatian Yu] on tumour necrosis factor-alpha (TNF-alpha)-induced necroptosis in cerebral ischaemia-reperfusion injury (CIRI) after cardiac arrest (CA). Material(s) and Method(s): Male Sprague Dawley rats were randomly assigned to six groups: sham group, PP0-L (10 mg/kg), PPO-M (20 mg/kg), PPO-H (40 mg/kg) and two control groups (CA, 0.9% saline; Gly, 10% glycerol). All drugs were administered intravenously to the CA/CPR rats within 10 min after return of spontaneous circulation (ROSC). After 24 h, rats were assessed for neuronal injury via the neurological deficit score (NDS), cerebral cortex staining and transmission electron microscopy (TEM) and expression levels of TNF-alpha and necroptosis-related proteins by immunoreactivity staining and western blotting. Result(s): Compared to those in the sham group (survival rate, 100% and NDS, 80), the survival rate and NDS were significantly reduced in the model groups (CA, 56.25%, 70; Gly, 62.5%, 71; PPO-L, 75%, 72; PPO-M, 87.5%, 75; PPO-H, 81.25%, 74). In the PPO-M group, Nissl bodies were significantly increased (43.67 +/- 1.906 vs. 17 +/- 1.732), the incidence of pathomorphological injury was lower and the necroptosis markers (TNF-alpha, RIPK1, RIPK3, p-MLKL/MLKL) expression was downregulated compared to those in the CA group ($p < 0.05$). Discussion and conclusions: The neuroprotective effects of PPO in the CA rats suggested that PPO

possibility as a health product enhances the resistance ability against brain injury for humans.Copyright © 2021 The Author(s). Published by Informa UK Limited, trading as Taylor & Francis Group.

61. Y., H., X., G., X., Z., Y., Z., Z.T., T., Zhu S B, … Zhu, S. (2021). **Remote Ischemic Postconditioning Inhibited Mitophagy to Achieve Neuroprotective Effects in the Rat Model of Cardiac Arrest**. *Neurochemical Research*, *46*(3), 573–583. https://doi.org/http://dx.doi.org/10.1007/s11064-020-03193-x
Remote ischemic postconditioning (RI-postC) is an effective measure to improve nerve function after cardiac arrest. However, the brain protective mechanism of RI-postC has not been fully elucidated, and whether it is related to mitophagy is unclear. In this study, we used the rat model of cardiac arrest to study the effect of RI-postC on mitophagy and explore its possible signaling pathways. Rats were randomly divided into Sham group, CA/CPR group, Mdivi-1 group and RI-postC group. The animal model of cardiac arrest was established by asphyxia. RI-postC was performed by clamping and loosening the left femoral artery. Mdivi-1 was treated with a single intravenous injection. Levels of TOMM20, TIM23, Mfn1, PINK1 and parkin were detected by western blots. Mitochondrial membrane potential was measured by flow cytometry. Real-time PCR was used to detect relative mitochondrial DNA levels. The apoptosis of hippocampal neurons was detected by flow and TUNEL. In addition, Histopathological tests were performed. The results showed that RI-postC was similar to the mitophagy inhibitor Mdivi-1, which could inhibit the decrease of mitophagy-related protein level, improve mitochondrial membrane potential and up-regulate the ratio of mt-Atp6/Rpl13 after cardiopulmonary resuscitation (CPR). Furthermore, RI-postC could also reduce the rate of hippocampal nerve apoptosis and the damage of hippocampal neurons after CPR. Moreover, RI-postC and Mdivi-1 could reduce the protein levels of PINK1 and parkin in mitochondria after CPR, while increasing PINK1 levels in the cytoplasm. These findings suggested that RI-postC could inhibit the overactivation mitophagy through the PINK1/parkin signaling pathway, thus providing neuroprotective effects.Copyright © 2021, The Author(s), under exclusive licence to Springer Science+Business Media, LLC part of Springer Nature.

62. Goncalves, C. L., Abelaira, H. M., Rosa, T., de Moura, A. B., Veron, D. C., Borba, L. A., … Reus G Z. (2021). **Ketamine treatment protects against oxidative damage and the immunological response induced by electroconvulsive therapy.** *Pharmacological Reports : PR*, *73*(2), 525–535. https://doi.org/http://dx.doi.org/10.1007/s43440-020-00200-4
BACKGROUND: Electroconvulsive therapy (ECT) is often recommended for major depressive disorder (MDD) for those who do not respond to the first and second antidepressant trials. A combination of two therapies could improve antidepressant efficacy. Thus, this study aimed to investigate the synergistic effects of ECT combined to antidepressants with a different mechanism of action., METHODS: Rats were treated once a day, for five days with ketamine (5 mg/kg), fluoxetine (1 mg/kg), and bupropion (4 mg/kg) alone or in combination with ECT (1 mA; 100 V). After, oxidative damage and antioxidant capacity were assessed in the prefrontal cortex (PFC) and hippocampus, and pro-inflammatory cytokines levels were evaluated in the serum., RESULTS: ECT alone increased lipid peroxidation in the PFC and hippocampus. In the PFC of rats treated with ECT in combination with fluoxetine and bupropion, and in the hippocampus of rats treated with ECT combined with ketamine and bupropion there was a reduction in the lipid peroxidation. The nitrite/nitrate was increased by ECT alone but reverted by combination with ketamine in the hippocampus. Superoxide dismutase (SOD) was increased by ECT and maintained by fluoxetine and bupropion in the PFC. ECT alone increased interleukin-1beta (IL-1beta) and the administration of ketamine was able to revert this increase showing a neuroprotective effect of this drug when in combination with ECT., CONCLUSION: The treatment with ECT leads to an increase in oxidative damage and alters the immunological system. The combination with ketamine was able to protect against oxidative damage and the immunological response induced by ECT.

63. J.S., O., J., P., K., K., H.H., J., Y.M., O., S., C., … Choi, K. H. (2021). **HSP70-mediated neuroprotection by combined treatment of valproic acid with hypothermia in a rat asphyxial cardiac arrest model**. *PLoS ONE*, *16*(6), e0253328. https://doi.org/http://dx.doi.org/10.1371/journal.pone.0253328
It has been reported that valproic acid (VPA) combined with therapeutic hypothermia can improve survival and neurologic outcomes in a rat asphyxial cardiac arrest model. However, neuroprotective mechanisms of such combined treatment of valproic acid with hypothermia remains unclear. We hypothesized that epigenetic regulation of HSP70 by histone acetylation could increase HSP70-mediated neuroprotection suppressed under hypothermia. Male Sprague-Dawley rats that achieved return of spontaneous circulation (ROSC) from asphyxial cardiac arrest were randomized to four groups: normothermia (37degreeC +/- 1degreeC), hypothermia (33degreeC +/- 1degreeC), normothermia + VPA (300 mg/kg IV initiated 5 minutes post-ROSC and infused over 20 min), and hypothermia + VPA. Three hours after ROSC, acetyl-histone H3 was highly expressed in VPA-administered groups (normothermia + VPA, hypothermia + VPA). Four hours after ROSC, HSP70 mRNA expression levels were significantly higher in normothermic groups (normothermia, normothermia + VPA) than in hypothermic groups (hypothermia, hypothermia + VPA). The hypothermia + VPA group showed significantly higher HSP70 mRNA expression than the hypothermia group. Similarly, at five hours after ROSC, HSP70 protein levels were significantly higher in normothermic groups than in hypothermic groups. HSP70 levels were significantly higher in the hypothermia + VPA group than in the hypothermia group. Only the hypothermia + VPA group showed significantly attenuated cleaved caspase-9 levels than the normothermia group. Hypothermia can attenuate the expression of HSP70 at transcriptional level. However, VPA administration can induce hyperacetylation of histone H3, leading to epigenetic transcriptional activation of HSP70 even in a hypothermic status. Combining VPA treatment with

hypothermia may compensate for reduced activation of HSP70-mediated anti-apoptotic pathway.Copyright © 2021 Oh et al. This is an open access article distributed under the terms of the Creative Commons Attribution License, which permits unrestricted use, distribution, and reproduction in any medium, provided the original author and source are credited.

64. Dietrichs, E. S., Myles, R., Smith, G., E.S., D., R., M., & Smith G. (2021). **Is hypothermia more neuroprotective than avoiding fever after cardiac arrest?.** *Cardiovascular Research*, *117*(12), E159–E161. https://doi.org/https://dx.doi.org/10.1093/cvr/cvab297

65. Venkataramaiah, C., Payani, S., Priya, B. L., Pradeepkiran, J. A., C., V., S., P., … Pradeepkiran J A. (2021). **Therapeutic potentiality of a new flavonoid against ketamine induced glutamatergic dysregulation in schizophrenia: In vivo and in silico approach.** *Biomedicine & Pharmacotherapy = Biomedecine & Pharmacotherapie*, *138*, 111453. https://doi.org/https://dx.doi.org/10.1016/j.biopha.2021.111453
Glutamate and dopamine hypotheses are leading theories of the pathophysiology of schizophrenia. Multiple lines of evidence suggest that dopaminergic and glutamatergic dysfunction is an underlying mechanism in schizophrenia. Since currently available antipsychotic drugs have significant untoward side effects, identification of new neuroprotective compounds from the medicinal plants may prove beneficial in neurodegenerative disorders. In our previous investigation we have isolated, characterized and reported a novel bioactive compound viz. 3-(3, 4-dimethoxy phenyl)-1-(4-methoxy phenyl) prop-2-en-1-one from the Celastrus paniculatus (CP) is used for the current clinical intervention of schizophrenia disease. The present study is mainly aimed to evaluate the neuroprotective potential of the above bioactive compound against ketamine-induced schizophrenia with particular reference to glutamate metabolism using in vivo and in silico methods. The decrease in glutamine content and the activity levels of glutamate dehydrogenase, glutamine synthetase, and glutaminase in different regions of the rat brain suggests lowered oxidative deamination and lowered mobilization of glutamate towards glutamine formation during ketamine-induced schizophrenia. Pre-treatment with the plant compound reversed the alterations in glutamate metabolism and restored the normal glutamatergic neurotransmission akin to the reference drug, clozapine. In addition, the compound has shown strong interaction and exhibited the highest binding energies against selected NMDA receptors with the lowest inhibition constant than the reference drug. Recoveries of these parameters during anti-schizophrenic treatment suggest that administration of plant compound might offer neuroprotection by interrupting the pathological cascade of glutamatergic neurotransmission that occurs during schizophrenia.Copyright © 2021

66. Yang, S., Yu, C., Yang, Z., Cui, H., Wu, Y., Liang, Z., … Tang Z. AO - Tang, Z. O. http://orcid. org/000.-0002-0330-4099. (2021). **DL-3-n-butylphthalide-induced neuroprotection in rat models of asphyxia-induced cardiac arrest followed by cardiopulmonary resuscitation.** *Journal of Cellular Physiology*, *236*(11), 7464–7472. https://doi.org/http://dx.doi.org/10.1002/jcp.30442
Most patients that resuscitate successfully from cardiac arrest (CA) suffer from poor neurological prognosis. DL-3-n-butylphthalide (NBP) is known to have neuroprotective effects via multiple mechanisms. This study aimed to investigate whether NBP can decrease neurological impairment after CA. We studied the protective role of NBP in the hippocampus of a rat model of cardiac arrest induced by asphyxia. Thirty-nine rats were divided randomly into sham, control, and NBP groups. Rats in control and NBP groups underwent cardiopulmonary resuscitation (CPR) 6 min after asphyxia. NBP or vehicle (saline) was administered intravenously 10 min after the return of spontaneous circulation (ROSC). Ultrastructure of hippocampal neurons was observed under transmission electron microscope. NBP treatment improved neurological function up to 72 h after CA. The ultrastructural lesion in mitochondria recovered in the NBP-treated CA model. In conclusion, our study demonstrated multiple therapeutic benefits of NBP after CA. Copyright © 2021 Wiley Periodicals LLC.

67. Dourado, L. F. N., Oliveira, L. G., da Silva, C. N., Toledo, C. R., Fialho, S. L., Jorge, R., & Silva-Cunha, A. J. (2021). **Intravitreal ketamine promotes neuroprotection in rat eyes after experimental ischemia.** *Biomedicine & Pharmacotherapy = Biomedecine & Pharmacotherapie*, *133*, 110948. https://doi.org/https://dx.doi.org/10.1016/j.biopha.2020.110948
Retinal ischemia, one of the most common cause of visual loss, is associated with blood flow inadequacy and subsequent tissue injury. In this setting, some treatments that can counteract glutamate increase, arouse interest in ischemic pathogenesis. Ketamine, a potent N-methyl-d-aspartate (NMDA) receptor antagonist, provides a neuroprotective pathway via decreasing the excitotoxicity triggered by excess glutamatergic. Thus, the goal of this study was to evaluate the safety of intravitreal use of ketamine and their potential protective effects on retinal cells in retinal ischemia/reperfusion model. Initially, ketamine toxicity was evaluated by cytotoxicity assay and Hen's egg chorioallantoic membrane (HET-CAM) method. Afterward, some ketamine concentrations were tested in rat's eyes to verify the safety of the intravitreal use. To investigate the neuroprotective effect on retinal, a single intravitreal injection of ketamine in concentrations of 0.059 mmol.L-1 and 0.118 mmol.L-1 was performed one day before the retinal injury by ischemia/reperfusion model. After 7 and 15 days, the retina activity was evaluated by electroretinogram (ERG) records and, lastly, by morphological analyzes. Cytotoxicity assay reveals that the maximum ketamine concentration that could reach retinal pigmented epithelium cells is 0.353 mmol.L-1. HET-CAM assay showed that concentrations above 0.237 mmol.L-1 are irritants to the eye. Thus, Ketamine in concentrations of 0.0237 mmol.L-1, 0.118 mmol.L-1, and 0.059

mmol.L-1 were selected for in vivo toxicity test. ERG records reveal a tendency of b-wave amplitude to decrease as the luminous intensity increased, in the group receiving ketamine at 0.237 mmol.L-1. Therefore, ketamine in concentrations at 0.059 mmol.L-1 and 0.118 mmol.L-1 were chosen for the following tests. In the ischemia retinal degeneration model, pretreatment with ketamine was capable to promote a recovery of retinal electrophysiological function minimizing the ischemic effects. In histological analysis, the groups that received intravitreal ketamine showed a number of retinal cells significantly higher than the vehicle group. In TUNEL assay a reduction on TUNEL-positive cells was observed in all the layers for both concentrations which allow to affirm that ketamine contributes to reducing cell death in the retina. Transmission electron microscopy (TEM) reaffirms this finding. Ketamine intravitreal pretreatment showed reduced ultrastructural changes. Our findings demonst…

68. Park, D. H., Kim, T. W., Kim, M. S., Han, W., Lee, D. E., Kim, G. S., & Jeong, C. Y. (2021). **Cardiac arrest caused by accidental severe hypothermia and myocardial infarction during general anesthesia.** *The Journal of International Medical Research*, *49*(1), 300060520987945. https://doi.org/https://dx.doi.org/10.1177/0300060520987945
Therapeutic hypothermia is often used for traumatic brain injury because of its neuroprotective effect and decreased secondary brain injury. However, this procedure lacks clinical evidence supporting its efficacy, and adverse outcomes have been reported during general anesthesia. A 61-year-old man with a history of percutaneous coronary intervention (PCI) was admitted with traumatic brain injury. Immediately after admission, he underwent mild therapeutic hypothermia with a target temperature of 33.0degreeC for neuroprotection. During general anesthesia for emergency surgery because he developed a mass effect, hypothermic cardiac arrest occurred following an additional decrease in the core body temperature. Moreover, myocardial infarction caused by restenosis of the previous PCI lesion also contributed to the cardiac arrest. Although the patient recovered spontaneous circulation after an hour-long cardiopulmonary resuscitation with rewarming, he eventually died of subsequent repetitive cardiac arrests. When anesthetizing patients undergoing therapeutic hypothermia, caution is required to prevent adverse outcomes that can be caused by unintentional severe hypothermia and exacerbation of underlying heart disease.

69. Taghizadehghalehjoughi, A., Naldan, M. E., A., T., & Naldan M E. (2021). **Is Ketamine Suitable for Use in Glutamate Toxicity Conditions?: An In Vitro Study**. *Journal of Investigative Surgery*, *34*(2), 121–128. https://doi.org/http://dx.doi.org/10.1080/08941939.2019.1582739
Ketamine is an anesthetic agent with sedative and analgesic properties frequently used in surgery. However, particular anesthetic substances need to be applied for different diseases and surgical procedures. Can ketamine be used in all operations and in all patients with an additional disease? The purpose of this study was to determine the neurotoxic or neuroprotective effects of different dosages of ketamine in a glutamate-derived toxicity model in olfactory, cortex and cerebellum cell cultures. Glutamate 10-5mM was added to all culture groups with the exception of the negative control group. Cells were exposed to four different dosages of ketamine for 24 h. At the end of the experiment, analyses were conducted using MTT, total antioxidant capacity (TAC), total oxidant status (TOS) and flow cytometry (annexin V apoptosis marker) tests. The highest viability rate was obtained at the lowest ketamine dosage, at approximately 80% in cerebellum cells, but less than 75% in cortex and olfactory culture cells. Based on our study findings, although ketamine is an NMDA antagonist, it causes an increase in toxicity levels and a decrease in cell viability. Ketamine use should therefore be avoided in neurological events in which glutamate levels increase significantly.Copyright © 2019 Taylor & Francis Group, LLC.

70. Magliocca, A., & Fries, M. (2021). **Inhaled gases as novel neuroprotective therapies in the postcardiac arrest period.** *Current Opinion in Critical Care*, *27*(3), 255–260. https://doi.org/https://dx.doi.org/10.1097/MCC.0000000000000820
PURPOSE OF REVIEW: The purpose of this review is to summarize recent advances about inhaled gases as novel neuroprotective agents in the postcardiac arrest period., RECENT FINDINGS: Inhaled gases, as nitric oxide (NO) and molecular hydrogen (H2), and noble gases as xenon (Xe) and argon (Ar) have shown neuroprotective properties after resuscitation. In experimental settings, the protective effect of these gases has been demonstrated in both in-vitro studies and animal models of cardiac arrest. They attenuate neuronal degeneration and improve neurological function after resuscitation acting on different pathophysiological pathways. Safety of both Xe and H2 after cardiac arrest has been reported in phase 1 clinical trials. A randomized phase 2 clinical trial showed the neuroprotective effects of Xe, combined with targeted temperature management. Xe inhalation for 24 h after resuscitation preserves white matter integrity as measured by fractional anisotropy of diffusion tensor MRI., SUMMARY: Inhaled gases, as Xe, Ar, NO, and H2 have consistently shown neuroprotective effects in experimental studies. Ventilation with these gases appears to be well tolerated in pigs and in preliminary human trials. Results from phase 2 and 3 clinical trials are needed to assess their efficacy in the treatment of postcardiac arrest brain injury. Copyright © 2021 Wolters Kluwer Health, Inc. All rights reserved.

71. Fraga, D. B., Camargo, A., Olescowicz, G., Azevedo Padilha, D., Mina, F., Budni, J., … Rodrigues, A. L. S. (2021). **A single administration of ascorbic acid rapidly reverses depressive-like behavior and hippocampal synaptic dysfunction induced by corticosterone in mice.** *Chemico-Biological Interactions*, *342*, 109476. https://doi.org/https://dx.doi.org/10.1016/j.cbi.2021.109476

Literature Search Results

Ketamine is the prototype for glutamate-based fast-acting antidepressants. The establishment of ketamine-like drugs is still a challenge and ascorbic acid has emerged as a candidate. This study investigated the ascorbic acid's ability to induce a fast antidepressant-like response and to improve hippocampal synaptic markers in mice subjected to chronic corticosterone (CORT) administration. CORT was administered for 21 days, followed by a single administration of ascorbic acid (1 mg /Kg, p.o.), ketamine (1 mg /Kg, i.p.) or fluoxetine (10 mg /Kg, p.o.) in mice. Depressive-like behavior, hippocampal synaptic proteins immunocontent, dendrite spines density in the dentate gyrus (DG) were analyzed 24 h following treatments. The administration of ascorbic acid or ketamine, but not fluoxetine, counteracted CORT-induced depressive-like behavior in the tail suspension test (TST). CORT administration reduced PSD-95, GluA1, and synapsin (synaptic markers) immunocontent, and these alterations were reversed by ascorbic acid or ketamine, but only ketamine reversed the CORT-induced reduction on GluA1 immunocontent. In the ventral and dorsal DG, CORT decreased filopodia-, thin- and stubby-shaped spines, while ascorbic acid and ketamine abolished this alteration only in filopodia spines. Ascorbic acid and ketamine increased mushroom-shaped spines density in ventral and dorsal DG. Therefore, the results show that a single administration of ascorbic acid, in a way similar to ketamine, rapidly elicits an antidepressant-like response and reverses hippocampal synaptic deficits caused by CORT, an effect associated with increased levels of synaptic proteins and dendritic remodeling. Copyright © 2021 Elsevier B.V. All rights reserved.
