# Supplementary material for: The use of ketamine as a neuroprotective agent following cardiac arrest: A scoping review of current literature
Source: CNS Neurosci Ther. 2022 Oct 2;29(1):104–10. doi: 10.1111/cns.13983 (PMC9804040; doi:10.1111/cns.13983)
Supplement: Supplementary file 2 — Appendix S2 [file CNS-29-104-s001.pdf]

Literature Search Results

Date Completed: 2021-05-28
Requestor: Marlena Ornowska

Request: Update search from 2020-05-28 (Request ID 9252)

1) information about cardiac arrest as a model used for neuro-protection studies. Most of the time in these types of studies, a stroke model is used. We are interested in finding out whether anyone has investigated usage of cardiac arrest as a model for neurological injury.

2) Neuronal injury following cardiac arrest and neuro-protection.

3) General literature search about ketamine in clinical practice in neuroprotection. Perhaps this search can include the following terms: ketamine, neuroprotection, experimental (any work)?

Completed By: Brooke Ballantyne Scott

Search Strategy:

Databases Searched:
Medline
EMBASE
CINAHL
Cochrane Database of Systematic Reviews
Cochrane Central Register of Controlled Trials

Terms searched:

MeSH:
Ketamine
Neuroprotective Agents
Neuroprotection
Heart Arrest

CINAHL:
Ketamine
Neuroprotective Agents
Heart Arrest

Embase SH:
ketamine
neuroprotection
heart arrest

Keywords:
neuron*
n3 injur*
neuroprotect*

Results:

1. Mart, M. F., Williams Roberson, S., Salas, B., Pandharipande, P. P., & Ely, E. W. (2021). **Prevention and Management of Delirium in the Intensive Care Unit**. *Seminars in Respiratory and Critical Care Medicine*, *42*(1), 112–126. https://doi.org/http://dx.doi.org/10.1055/s-0040-1710572
Delirium is a debilitating form of brain dysfunction frequently encountered in the intensive care unit (ICU). It is associated with increased morbidity and mortality, longer lengths of stay, higher hospital costs, and cognitive impairment that persists long after hospital discharge. Predisposing factors include smoking, hypertension, cardiac disease, sepsis, and premorbid dementia. Precipitating factors include respiratory failure and shock, metabolic disturbances, prolonged mechanical ventilation, pain, immobility, and sedatives and adverse environmental conditions impairing vision, hearing, and sleep. Historically, antipsychotic medications were the mainstay of delirium treatment in

the critically ill. Based on more recent literature, the current Society of Critical Care Medicine (SCCM) guidelines suggest against routine use of antipsychotics for delirium in critically ill adults. Other pharmacologic interventions (e.g., dexmedetomidine) are under investigation and their impact is not yet clear. Nonpharmacologic interventions thus remain the cornerstone of delirium management. This approach is summarized in the ABCDEF bundle (A ssess, prevent, and manage pain; B oth SAT and SBT; C hoice of analgesia and sedation; D elirium: assess, prevent, and manage; E arly mobility and exercise; F amily engagement and empowerment). The implementation of this bundle reduces the odds of developing delirium and the chances of needing mechanical ventilation, yet there are challenges to its implementation. There is an urgent need for ongoing studies to more effectively mitigate risk factors and to better understand the pathobiology underlying ICU delirium so as to identify additional potential treatments. Further refinements of therapeutic options, from drugs to rehabilitation, are current areas ripe for study to improve the short- and long-term outcomes of critically ill patients with delirium.Copyright © 2021 BMJ Publishing Group. All rights reserved.

2. Benedetto, U., Dimagli, A., Sinha, S., Fudulu, D., Angelini, G. D., Cooper, G., … Tsang Umberto; ORCID: http://orcid.org/0000-0002-7074-7949 AO - Dimagli, Arnaldo; ORCID: http://orcid.org/0000-0003-0055-8656 AO - Sinha, Shubhra; ORCID: http://orcid.org/0000-0001-8554-6704 AO - Angelini, Gianni D.; ORCID: http://orcid.org/0000-0002-1753-3730 AO - Krasopoulos, George; ORCID: http://orcid.org/0000-0001-9334-9604 AO - Kendall, Simon; ORCID: http://orcid.org/0000-0002-1222-6483 AO - Akowuah, Enoch; ORCID: http://orcid.org/0000-0002-2429-3579 AO - Trivedi, Uday; ORCID: http://orcid.org/0000-0002-7078-2760, G. A. O.-B. (2021). **Neuroprotective strategies in acute aortic dissection: an analysis of the UK National Adult Cardiac Surgical Audit**. *European Journal of Cardio-Thoracic Surgery : Official Journal of the European Association for Cardio-Thoracic Surgery*. https://doi.org/http://dx.doi.org/10.1093/ejcts/ezab192
OBJECTIVES: The risk of brain injury following surgery for type A aortic dissection (TAAD) remains substantial and no consensus has still been reached on which neuroprotective technique should be preferred. We aimed to investigate the association between neuroprotective strategies and clinical outcomes following TAAD repair. METHOD(S): Using the UK National Adult Cardiac Surgical Audit, we identified 1929 patients undergoing surgery for TAAD (2011-2018). Deep hypothermic circulatory arrest (DHCA) only, unilateral (uACP), bilateral antegrade cerebral perfusion (bACP) and retrograde cerebral perfusion were used in 830, 117, 760 and 222 patients, respectively. The primary end point was a composite of death and/or cerebrovascular accident (CVA). Generalized linear mixed model was used to adjust the effect of neuroprotective strategies for other confounders. RESULT(S): The use of bACP was associated with longer circulatory arrest (CA) compared to other strategies. There was a trend towards lower incidence of death and/or CVA using uACP only for shorter CA. In particular, primary end point rate was 27.7% overall and 26.5%, 12.5%, 28.0% and 22.9% for CA <30min and 28.6%, 30.4%, 33.3% and 33.0% for CA >=30min with DHCA only, uACP, bACP and retrograde cerebral perfusion, respectively. The use of DHCA only was associated with five-fold [odds ratio (OR) 5.35, 95% confidence interval (CI) 1.36-21.02] and two-fold (OR 1.77, 95% CI 1.01-3.09) increased risk of death and/or CVA compared to uACP and bACP, respectively, but the effect of uACP was significantly associated with CA duration (hazard ratio 0.97, 95% CI 0.94-0.99; P=0.04). CONCLUSION(S): In TAAD repair, the use of uACP and bACP was associated with a lower adjusted risk of death and/or CVA when compared to DHCA. uACP can offer some advantage but only for a shorter CA duration.Copyright © The Author(s) 2021. Published by Oxford University Press on behalf of the European Association for Cardio-Thoracic Surgery. All rights reserved.

3. Anoop, U. R., & Verma, K. (2021). **New technique and device for controlled and continuous drug delivery into the brain: A proof-of-concept study**. *BMJ Innovations*, *7*(2), 470–477. https://doi.org/http://dx.doi.org/10.1136/bmjinnov-2019-000393

4. Wouters, A., Scheldeman, L., Plessers, S., Peeters, R., Cappelle, S., Demaerel, P., … Lemmens Sarah; ORCID: http://orcid.org/0000-0002-6781-4776, R. A. O.-C. (2021). **Added Value of Quantitative Apparent Diffusion Coefficient Values for Neuroprognostication After Cardiac Arrest**. *Neurology*. https://doi.org/http://dx.doi.org/10.1212/WNL.0000000000011991
OBJECTIVE: To test the prognostic value of brain MRI in addition to clinical and electrophysiological variables in post-cardiac arrest (CA) patients, we explored data from the randomized Neuroprotect post-CA trial (NCT02541591). METHOD(S): In this trial brain MRI's were prospectively obtained. We calculated receiver operating characteristic curves for the average Apparent Diffusion Coefficient (ADC) value and percentage of brain voxels with an ADC value < 650 x 10-6 mm2/s and < 450 x 10-6 mm2/s. We constructed multivariable logistic regression models with clinical characteristics, electroencephalogram (EEG), somatosensory evoked potentials (SSEP) and ADC value as independent variables, to predict good neurological recovery. RESULT(S): In 79/102 patients MRI data were available and in 58/79 patients all other data were available. At 180 days post-CA, 25/58 (43%) patients had good neurological recovery. In univariable analysis of all tested MRI parameters, average ADC value in the postcentral cortex had the highest accuracy to predict good neurological recovery with an AUC of 0.78. In the most optimal multivariate model which also included corneal reflexes and EEG, this parameter remained an independent predictor of good neurological recovery (AUC = 0.96, false positive = 27%). This model provided a more accurate prediction compared to the most optimal combination of EEG, corneal reflexes and SSEP (p=0.03). CONCLUSION(S): Adding information on brain MRI in a multivariate model may improve the prediction of good neurological recovery in post-CA patients. CLASSIFICATION OF EVIDENCE: "This study provides Class III evidence that MRI ADC features predict neurological recovery in post-cardiac arrest

patients."Copyright © 2021 American Academy of Neurology.

5. Ma, Q., Feng, L., Wang, T., Li, Y., Li, Z., Zhao, B., … Fan, D. (2021). **2020 expert consensus statement on neuro-protection after cardiac arrest in China**. *Annals of Translational Medicine*, *9*(2), 175. https://doi.org/http://dx.doi.org/10.21037/atm-20-7853

6. Momeni, M., Khalifa, C., Lemaire, G., Watremez, C., Tircoveanu, R., Van Dyck, M., … Jacquet, L.-M. (2021). **Propofol plus low-dose dexmedetomidine infusion and postoperative delirium in older patients undergoing cardiac surgery**. *British Journal of Anaesthesia*, *126*(3), 665–673. https://doi.org/http://dx.doi.org/10.1016/j.bja.2020.10.041
Background: Postoperative delirium (POD) is a frequent complication in older patients. Dexmedetomidine might be effective in decreasing the incidence of POD. We hypothesised that adding low-dose rate dexmedetomidine infusion to a propofol sedation regimen would have fewer side-effects and would counteract the possible delirium producing properties of propofol, resulting in a lower risk of POD than propofol with placebo. Method(s): In this double-blind placebo-controlled trial, patients >=60 yr old undergoing on-pump cardiac surgery were randomised 1:1 to the following postoperative sedative regimens: a propofol infusion and dexmedetomidine (0.4 mug kg-1 h-1) or a propofol infusion and saline 0.9% (placebo group). The study drug was started at chest closure and continued for 10 h. The primary endpoint was in-hospital POD, assessed using the Confusion Assessment Method and chart review method. Result(s): POD over the course of hospital stay occurred in 31/177 (18%) and 33/172 (19%) patients in the dexmedetomidine and placebo arm, respectively (P=0.687; odds ratio=0.89; 95% confidence interval, 0.52-1.54). The incidence of POD in the intensive care alone, or on the ward alone, was also not significantly different between the groups. Subjects in the dexmedetomidine group spent less median time in a delirious state (P=0.026). Median administered postoperative norepinephrine was significantly higher in the dexmedetomidine group (P<0.001). One patient in the dexmedetomidine group and 10 patients in the placebo group died in the hospital. Conclusion(s): Adding low-dose rate dexmedetomidine to a sedative regimen based on propofol did not result in a different risk of in-hospital delirium in older patients undergoing cardiac surgery. With a suggestion of both harm and benefit in secondary outcomes, supplementing postoperative propofol with dexmedetomidine cannot be recommended based on this study. Clinical trial registration: NCT03388541.Copyright © 2020 British Journal of Anaesthesia

7. Taghizadehghalehjoughi, A., & Naldan, M. E. (2021). **Is Ketamine Suitable for Use in Glutamate Toxicity Conditions?: An In Vitro Study**. *Journal of Investigative Surgery*, *34*(2), 121–128. https://doi.org/http://dx.doi.org/10.1080/08941939.2019.1582739
Ketamine is an anesthetic agent with sedative and analgesic properties frequently used in surgery. However, particular anesthetic substances need to be applied for different diseases and surgical procedures. Can ketamine be used in all operations and in all patients with an additional disease? The purpose of this study was to determine the neurotoxic or neuroprotective effects of different dosages of ketamine in a glutamate-derived toxicity model in olfactory, cortex and cerebellum cell cultures. Glutamate 10-5mM was added to all culture groups with the exception of the negative control group. Cells were exposed to four different dosages of ketamine for 24 h. At the end of the experiment, analyses were conducted using MTT, total antioxidant capacity (TAC), total oxidant status (TOS) and flow cytometry (annexin V apoptosis marker) tests. The highest viability rate was obtained at the lowest ketamine dosage, at approximately 80% in cerebellum cells, but less than 75% in cortex and olfactory culture cells. Based on our study findings, although ketamine is an NMDA antagonist, it causes an increase in toxicity levels and a decrease in cell viability. Ketamine use should therefore be avoided in neurological events in which glutamate levels increase significantly.Copyright © 2019 Taylor & Francis Group, LLC.

8. Lee, J. K., Santos, P. T., O'Brien, C. E., Kulikowicz, E., Adams, S., Hardart, H., … Martin, L. J. (2021). **Combining hypothermia and oleuropein subacutely protects subcortical white matter in a swine model of neonatal hypoxic-ischemic encephalopathy**. *Journal of Neuropathology and Experimental Neurology*, *80*(2), 182–198. https://doi.org/http://dx.doi.org/10.1093/jnen/nlaa132
Neonatal hypoxia-ischemia (HI) causes white matter injury that is not fully prevented by therapeutic hypothermia. Adjuvant treatments are needed. We compared myelination in different piglet white matter regions. We then tested whether oleuropein (OLE) improves neuroprotection in 2- to 4-day-old piglets randomized to undergo HI or sham procedure and OLE or vehicle administration beginning at 15 minutes. All groups received overnight hypothermia and rewarming. Injury in the subcortical white matter, corpus callosum, internal capsule, putamen, and motor cortex gray matter was assessed 1 day later. At baseline, piglets had greater subcortical myelination than in corpus callosum. Hypothermic HI piglets had scant injury in putamen and cerebral cortex. However, hypothermia alone did not prevent the loss of subcortical myelinating oligodendrocytes or the reduction in subcortical myelin density after HI. Combining OLE with hypothermia improved post-HI subcortical white matter protection by preserving myelinating oligodendrocytes, myelin density, and oligodendrocyte markers. Corpus callosum and internal capsule showed little HI injury after hypothermia, and OLE accordingly had minimal effect. OLE did not affect putamen or motor cortex neuron counts. Thus, OLE combined with hypothermia protected subcortical white matter after HI. As an adjuvant to hypothermia, OLE may subacutely improve regional white matter protection after HI.Copyright © 2020 American Association of Neuropathologists, Inc. All rights reserved.

Literature Search Results

9. Godoy, D. A., Badenes, R., Pelosi, P., & Robba, C. (2021). **Ketamine in acute phase of severe traumatic brain injury "an old drug for new uses?"** *Critical Care*, *25*(1), 19. https://doi.org/http://dx.doi.org/10.1186/s13054-020-03452-x
Maintaining an adequate level of sedation and analgesia plays a key role in the management of traumatic brain injury (TBI). To date, it is unclear which drug or combination of drugs is most effective in achieving these goals. Ketamine is an agent with attractive pharmacological and pharmacokinetics characteristics. Current evidence shows that ketamine does not increase and may instead decrease intracranial pressure, and its safety profile makes it a reliable tool in the prehospital environment. In this point of view, we discuss different aspects of the use of ketamine in the acute phase of TBI, with its potential benefits and pitfalls.Copyright © 2021, The Author(s).

10. Chen, S., Gao, L., Lachance, B. B., & Jia Xiaofeng; ORCID: http://orcid.org/0000-0003-1445-8525, X. A. O.-J. (2021). **Targeted temperature management and early neuro-prognostication after cardiac arrest**. *Journal of Cerebral Blood Flow and Metabolism*. https://doi.org/http://dx.doi.org/10.1177/0271678X20970059
Targeted temperature management (TTM) is a recommended neuroprotective intervention for coma after out-of-hospital cardiac arrest (OHCA). However, controversies exist concerning the proper implementation and overall efficacy of post-CA TTM, particularly related to optimal timing and depth of TTM and cooling methods. A review of the literature finds that optimizing and individualizing TTM remains an open question requiring further clinical investigation. This paper will summarize the preclinical and clinical trial data to-date, current recommendations, and future directions of this therapy, including new cooling methods under investigation. For now, early induction, maintenance for at least 24 hours, and slow rewarming utilizing endovascular methods may be preferred. Moreover, timely and accurate neuro-prognostication is valuable for guiding ethical and cost-effective management of post-CA coma. Current evidence for early neuro-prognostication after TTM suggests that a combination of initial prediction models, biomarkers, neuroimaging, and electrophysiological methods is the optimal strategy in predicting neurological functional outcomes.Copyright © The Author(s) 2021.

11. Mihaljevic, S., Pavlovic, M., Reiner, K., & Cacic, M. (2021). **Therapeutic mechanisms of ketamine**. *Psychiatria Danubina*, *32*(3–4), 325–333. https://doi.org/http://dx.doi.org/10.24869/PSYD.2020.325
Major depressive disorder is the greatest burden of developed countries in the context of morbidity caused by mental disorders. Until recent, ketamine has been mostly used for anesthesia, analgesia, sedation and treatment of chronic pain syndromes. However, unique pharmacodynamic properties of ketamine have increased interests in it's use for treatment of depression. It is assumed that ketamine reverses synaptic chronic stress pathology within one day of administration by postsynaptic glutamate activation, providing synaptic connectivity restoration that last for days or weeks. Potential glutamatergic agents, in context of treatment of major depressive disorder are not entirely novel phenomenon. Considering the aforementioned, current neurobiological view of depression as a solely monoaminergic phenomenon should be reassessed in order to prompt discovery of putative antidepressant drugs of novel generation. Acute side effects, such as increased salivation, increase in heart rate, systemic arterial pressure and intracranial pressure necessitate careful monitoring during intravenous administration of ketamine, even in subanesthetic doses. However, major burden of ketamine administration lies in it's ability to produce psychotomimetic side effects and emergence delirium. Esketamine nasal spray has now been widely approved and is considered safe in terms of acute side effects, tolerability and consistent therapeutic benefit.Copyright © 2020 Medicinska Naklada Zagreb. All rights reserved.

12. Park, H.-A., Broman, K., & Jonas, E. (2021). **Oxidative stress battles neuronal Bcl-xL in a fight to the death**. *Neural Regeneration Research*, *16*(1), 12–15. https://doi.org/http://dx.doi.org/10.4103/1673-5374.286946
Bcl-xL is a pro-survival protein of the Bcl2 family found in the mitochondrial membrane. Bcl-xL supports growth, development, and maturation of neurons, and it also prevents neuronal death during neurotoxic stimulation. This article reviews the mechanisms and upstream signaling that regulate the activity and abundance of Bcl-xL. Our team and others have reported that oxidative stress is a key regulator of intracellular Bcl-xL balance in neurons. Oxidative stress regulates synthesis, degradation, and activity of Bcl-xL and therefore neuronal function. During apoptosis, pro-apoptotic Bcl2 proteins such as Bax and Bak translocate to and oligomerize in the mitochondrial membrane. Formation of oligomers causes release of cytochrome c and activation of caspases that lead to neuronal death. Bcl-xL binds directly to pro-apoptotic Bcl2 proteins to block apoptotic signaling. Although anti-apoptotic roles of Bcl-xL have been well documented, an increasing number of studies in recent decades show that protein binding partners of Bcl-xL are not limited to Bcl2 proteins. Bcl-xL forms a complex with F1Fo ATP synthase, DJ-1, DRP1, IP3R, and the ryanodine receptor. These proteins support physiological processes in neurons such as growth and development and prevent neuronal damage by regulating mitochondrial ATP production, synapse formation, synaptic vesicle recycling, neurotransmission, and calcium signaling. However, under conditions of oxidative stress, Bcl-xL undergoes proteolytic cleavage thus lowering the abundance of functional Bcl-xL in neurons. Additionally, oxidative stress alters formation of Bcl-xL-mediated multiprotein complexes by regulating post-translational phosphorylation. Finally, oxidative stress regulates transcription factors that target the Bcl-x gene and alter accessibility of microRNA to mRNA influencing mRNA levels of Bcl-xL. In this review, we discussed how Bcl-xL supports the normal physiology of neurons, and how oxidative stress contributes to pathology by manipulating the dynamics of Bcl-xL production, degradation, and activity.Copyright © 2021 Wolters Kluwer Medknow Publications. All rights reserved.

13. Magliocca, A., & Fries, M. (2021). **Inhaled gases as novel neuroprotective therapies in the postcardiac arrest period.** *Current Opinion in Critical Care*, *27*(3), 255–260. https://doi.org/https://dx.doi.org/10.1097/MCC.0000000000000820
PURPOSE OF REVIEW: The purpose of this review is to summarize recent advances about inhaled gases as novel neuroprotective agents in the postcardiac arrest period., RECENT FINDINGS: Inhaled gases, as nitric oxide (NO) and molecular hydrogen (H2), and noble gases as xenon (Xe) and argon (Ar) have shown neuroprotective properties after resuscitation. In experimental settings, the protective effect of these gases has been demonstrated in both in-vitro studies and animal models of cardiac arrest. They attenuate neuronal degeneration and improve neurological function after resuscitation acting on different pathophysiological pathways. Safety of both Xe and H2 after cardiac arrest has been reported in phase 1 clinical trials. A randomized phase 2 clinical trial showed the neuroprotective effects of Xe, combined with targeted temperature management. Xe inhalation for 24 h after resuscitation preserves white matter integrity as measured by fractional anisotropy of diffusion tensor MRI., SUMMARY: Inhaled gases, as Xe, Ar, NO, and H2 have consistently shown neuroprotective effects in experimental studies. Ventilation with these gases appears to be well tolerated in pigs and in preliminary human trials. Results from phase 2 and 3 clinical trials are needed to assess their efficacy in the treatment of postcardiac arrest brain injury. Copyright © 2021 Wolters Kluwer Health, Inc. All rights reserved.

14. Fraga, D. B., Camargo, A., Olescowicz, G., Azevedo Padilha, D., Mina, F., Budni, J., … Rodrigues, A. L. S. (2021). **A single administration of ascorbic acid rapidly reverses depressive-like behavior and hippocampal synaptic dysfunction induced by corticosterone in mice.** *Chemico-Biological Interactions*, *342*, 109476. https://doi.org/https://dx.doi.org/10.1016/j.cbi.2021.109476
Ketamine is the prototype for glutamate-based fast-acting antidepressants. The establishment of ketamine-like drugs is still a challenge and ascorbic acid has emerged as a candidate. This study investigated the ascorbic acid's ability to induce a fast antidepressant-like response and to improve hippocampal synaptic markers in mice subjected to chronic corticosterone (CORT) administration. CORT was administered for 21 days, followed by a single administration of ascorbic acid (1 mg /Kg, p.o.), ketamine (1 mg /Kg, i.p.) or fluoxetine (10 mg /Kg, p.o.) in mice. Depressive-like behavior, hippocampal synaptic proteins immunocontent, dendrite spines density in the dentate gyrus (DG) were analyzed 24 h following treatments. The administration of ascorbic acid or ketamine, but not fluoxetine, counteracted CORT-induced depressive-like behavior in the tail suspension test (TST). CORT administration reduced PSD-95, GluA1, and synapsin (synaptic markers) immunocontent, and these alterations were reversed by ascorbic acid or ketamine, but only ketamine reversed the CORT-induced reduction on GluA1 immunocontent. In the ventral and dorsal DG, CORT decreased filopodia-, thin- and stubby-shaped spines, while ascorbic acid and ketamine abolished this alteration only in filopodia spines. Ascorbic acid and ketamine increased mushroom-shaped spines density in ventral and dorsal DG. Therefore, the results show that a single administration of ascorbic acid, in a way similar to ketamine, rapidly elicits an antidepressant-like response and reverses hippocampal synaptic deficits caused by CORT, an effect associated with increased levels of synaptic proteins and dendritic remodeling. Copyright © 2021 Elsevier B.V. All rights reserved.

15. Oshodi, T. O., Ben-Azu, B., Ishola, I. O., Ajayi, A. M., Emokpae, O., & Umukoro, S. (2021). **Molecular mechanisms involved in the prevention and reversal of ketamine-induced schizophrenia-like behavior by rutin: the role of glutamic acid decarboxylase isoform-67, cholinergic, Nox-2-oxidative stress pathways in mice.** *Molecular Biology Reports*, *48*(3), 2335–2350. https://doi.org/https://dx.doi.org/10.1007/s11033-021-06264-6
Mounting evidences have shown that nicotinamide adenine dinucleotide phosphate oxidase-2 (Nox-2) pathway modifies glutamic-acid decarboxylase-67 (GAD67) (GABAergic enzyme) and cholinergic systems via oxidative-nitrergic mechanisms in schizophrenia pathology. Rutin, a neuroactive antioxidant compound, with proven neuroprotective property has been shown to reduce schizophrenic-like behavior in mice. This study sought to investigate the mechanisms of action of the psychopharmacological activity of rutin in the preventive and reversal effects of ketamine-induced schizophrenic-like behavior, oxidative-nitrergic stress, cholinergic and GABAergic derangements in mice. In the preventive treatment, male mice were given rutin (0.1, 0.2 and 0.4 mg/kg) or risperidone (0.5 mg/kg) orally for 14 days prior to ketamine (20 mg/kg, i.p.) treatment from the 8 to 14th day. However, in the reversal treatment, ketamine was given for 14 days prior to rutin and risperidone. Behavioral (open-field, social-interaction and Y-maze tests), biochemical (oxidative/nitrergic stress markers, acetylcholinesterase activity), immunohistochemical (GAD67, Nox-2) and neuronal cell deaths in the striatum, prefrontal cortex, and hippocampus were evaluated. Ketamine-induced behavioral impairments were prevented and reversed by rutin. Exposure of mice to ketamine increased malondialdehyde, nitrite contents, acetylcholinesterase activity, neuronal cell death and Nox-2 expressions in the striatum, prefrontal cortex and hippocampus. Conversely, these derangements were prevented and reversed by rutin. The decreased glutathione levels due to ketamine were marked increased by rutin. Rutin only prevented ketamine-induced decrease in GAD67 expression in the striatal-hippocampal region. Altogether, the study showed that the prevention and reversal treatments of mice with rutin attenuated ketamine-induced schizophrenic-like behaviors via reduction of Nox-2 expression, oxidative/nitrergic stresses, acetylcholinesterase activity, and increased GAD67 enzyme.

16. Park, D. H., Kim, T. W., Kim, M. S., Han, W., Lee, D. E., Kim, G. S., & Jeong, C. Y. (2021). **Cardiac arrest caused by accidental severe hypothermia and myocardial infarction during general anesthesia.** *The Journal of*

International Medical Research, *49*(1), 300060520987945. https://doi.org/https://dx.doi.org/10.1177/0300060520987945
Therapeutic hypothermia is often used for traumatic brain injury because of its neuroprotective effect and decreased secondary brain injury. However, this procedure lacks clinical evidence supporting its efficacy, and adverse outcomes have been reported during general anesthesia. A 61-year-old man with a history of percutaneous coronary intervention (PCI) was admitted with traumatic brain injury. Immediately after admission, he underwent mild therapeutic hypothermia with a target temperature of 33.0degreeC for neuroprotection. During general anesthesia for emergency surgery because he developed a mass effect, hypothermic cardiac arrest occurred following an additional decrease in the core body temperature. Moreover, myocardial infarction caused by restenosis of the previous PCI lesion also contributed to the cardiac arrest. Although the patient recovered spontaneous circulation after an hour-long cardiopulmonary resuscitation with rewarming, he eventually died of subsequent repetitive cardiac arrests. When anesthetizing patients undergoing therapeutic hypothermia, caution is required to prevent adverse outcomes that can be caused by unintentional severe hypothermia and exacerbation of underlying heart disease.

17. Gore, A., Neufeld-Cohen, A., Egoz, I., Baranes, S., Gez, R., Efrati, R., … Lazar, S. (2021). **Neuroprotection by delayed triple therapy following sarin nerve agent insult in the rat.** *Toxicology and Applied Pharmacology*, *419*, 115519. https://doi.org/https://dx.doi.org/10.1016/j.taap.2021.115519
The development of refractory status epilepticus (SE) induced by sarin intoxication presents a therapeutic challenge. In our current research we evaluate the efficacy of a delayed combined triple treatment in ending the abnormal epileptiform seizure activity (ESA) and the ensuing of long-term neuronal insult. SE was induced in male Sprague-Dawley rats by exposure to 1.2LD50 sarin insufficiently treated by atropine and TMB4 (TA) 1 min later. Triple treatment of ketamine, midazolam and valproic acid was administered 30 min or 1 h post exposure and was compared to a delayed single treatment with midazolam alone. Toxicity and electrocorticogram activity were monitored during the first week and behavioral evaluation performed 3 weeks post exposure followed by brain biochemical and immunohistopathological analyses. The addition of both single and triple treatments reduced mortality and enhanced weight recovery compared to the TA-only treated group. The triple treatment also significantly minimized the duration of the ESA, reduced the sarin-induced increase in the neuroinflammatory marker PGE2, the brain damage marker TSPO, decreased the gliosis, astrocytosis and neuronal damage compared to the TA+ midazolam or only TA treated groups. Finally, the triple treatment eliminated the sarin exposed increased open field activity, as well as impairing recognition memory as seen in the other experimental groups. The delayed triple treatment may serve as an efficient therapy, which prevents brain insult propagation following sarin-induced refractory SE, even if treatment is postponed for up to 1 h. Copyright © 2021 Elsevier Inc. All rights reserved.

18. Dourado, L. F. N., Oliveira, L. G., da Silva, C. N., Toledo, C. R., Fialho, S. L., Jorge, R., & Silva-Cunha, A. J. (2021). **Intravitreal ketamine promotes neuroprotection in rat eyes after experimental ischemia.** *Biomedicine & Pharmacotherapy = Biomedecine & Pharmacotherapie*, *133*, 110948. https://doi.org/https://dx.doi.org/10.1016/j.biopha.2020.110948
Retinal ischemia, one of the most common cause of visual loss, is associated with blood flow inadequacy and subsequent tissue injury. In this setting, some treatments that can counteract glutamate increase, arouse interest in ischemic pathogenesis. Ketamine, a potent N-methyl-d-aspartate (NMDA) receptor antagonist, provides a neuroprotective pathway via decreasing the excitotoxicity triggered by excess glutamatergic. Thus, the goal of this study was to evaluate the safety of intravitreal use of ketamine and their potential protective effects on retinal cells in retinal ischemia/reperfusion model. Initially, ketamine toxicity was evaluated by cytotoxicity assay and Hen's egg chorioallantoic membrane (HET-CAM) method. Afterward, some ketamine concentrations were tested in rat's eyes to verify the safety of the intravitreal use. To investigate the neuroprotective effect on retinal, a single intravitreal injection of ketamine in concentrations of 0.059 mmol.L-1 and 0.118 mmol.L-1 was performed one day before the retinal injury by ischemia/reperfusion model. After 7 and 15 days, the retina activity was evaluated by electroretinogram (ERG) records and, lastly, by morphological analyzes. Cytotoxicity assay reveals that the maximum ketamine concentration that could reach retinal pigmented epithelium cells is 0.353 mmol.L-1. HET-CAM assay showed that concentrations above 0.237 mmol.L-1 are irritants to the eye. Thus, Ketamine in concentrations of 0.0237 mmol.L-1, 0.118 mmol.L-1, and 0.059 mmol.L-1 were selected for in vivo toxicity test. ERG records reveal a tendency of b-wave amplitude to decrease as the luminous intensity increased, in the group receiving ketamine at 0.237 mmol.L-1. Therefore, ketamine in concentrations at 0.059 mmol.L-1 and 0.118 mmol.L-1 were chosen for the following tests. In the ischemia retinal degeneration model, pretreatment with ketamine was capable to promote a recovery of retinal electrophysiological function minimizing the ischemic effects. In histological analysis, the groups that received intravitreal ketamine showed a number of retinal cells significantly higher than the vehicle group. In TUNEL assay a reduction on TUNEL-positive cells was observed in all the layers for both concentrations which allow to affirm that ketamine contributes to reducing cell death in the retina. Transmission electron microscopy (TEM) reaffirms this finding. Ketamine intravitreal pretreatment showed reduced ultrastructural changes. Our findings demonst…

19. Alqahtani, F., Assiri, M. A., Mohany, M., Imran, I., Javaid, S., Rasool, M. F., … Alamri, F. F. (2020). **Coadministration of Ketamine and Perampanel Improves Behavioral Function and Reduces Inflammation in Acute Traumatic Brain Injury Mouse Model.** *BioMed Research International*, 1–12.

https://doi.org/10.1155/2020/3193725
Traumatic brain injury (TBI) is among the most debilitating neurological disorders with inadequate therapeutic options. It affects all age groups globally leading to post-TBI behavioral challenges and life-long disabilities requiring interventions for these health issues. In the current study, C57BL/6J mice were induced with TBI through the weight-drop method, and outcomes of acutely administered ketamine alone and in combination with perampanel were observed. The impact of test drugs was evaluated for post-TBI behavioral changes by employing the open field test (OFT), Y-maze test, and novel object recognition test (NOR). After that, isolated plasma and brain homogenates were analyzed for inflammatory modulators, i.e., NF-κB and iNOS, through ELISA. Moreover, metabolomic studies were carried out to further authenticate the TBI rescuing potential of drugs. The animals treated with ketamine-perampanel combination demonstrated improved exploratory behavior in OFT ($P < 0.05$), while ketamine alone as well as in combination yielded anxiolytic effect ($P < 0.05 - 0.001$) in posttraumatic mice. Similarly, the % spontaneous alternation and % discrimination index were increased after the administration of ketamine alone ($P < 0.05$) and ketamine-perampanel combination ($P < 0.01 - 0.001$) in the Y-maze test and NOR test, respectively. ELISA demonstrated the reduced central and peripheral expression of NF-κB ($P < 0.05$) and iNOS ($P < 0.01 - 0.0001$) after ketamine-perampanel polypharmacy. The TBI-imparted alteration in plasma metabolites was restored by drug combination as evidenced by metabolomic studies. The outcomes were fruitful with ketamine, but the combination therapy proved more significant in improving all studied parameters. The benefits of this new investigated polypharmacy might be due to their antiglutamatergic, antioxidant, and neuroprotective capacity.

20. Wouters A Plessers S, Peeters R, Cappelle S, Demaerel P, Van Paesschen W, Ferdinande B, Dens J, Dupont M, Janssens S, Ameloot K, Lemmens R, S. L. (2020). **Role of neuroimaging in the prediction of outcome following outof-hospital cardiac arrest: insights from the neuroprotect trial**. *International Journal of Stroke*, *15*(1 SUPPL), 69. Retrieved from http://ovidsp.ovid.com/ovidweb.cgi?T=JS&PAGE=reference&D=cctr&NEWS=N&AN=CN-02244996
Background And Aims: Hypoxic-ischemic brain injury after cardiac arrest is a common cause of permanent neurological disability. We aimed to investigate the prognostic value of brain MRI after cardiac arrest in patients included in the randomized controlled NEUROPROTECT trial. Methods: The NEUROPROTECT trial investigated an early goal directed hemodynamic optimization strategy in patients after out-of-hospital cardiac arrest which failed the primary endpoint.We combined all patients and calculated apparent diffusion coefficient (ADC) values in different brain regions. We constructed multivariate logistic regression models with electroencephalogram (EEG), somatosensory evoked potentials (SSEP), clinical neurological examination and ADC values as independent variables, to predict good neurological recovery (independent activities of daily life) at 180 days post-cardiac arrest. Backward logistic regression was used to select significant variables. Results: We included 79/102 patients with available MRI data of whom 58 patients had documented brain stem reflexes, EEG and SSEP. In univariate analysis of all MRI parameters, the average ADC value in the postcentral cortex had the highest accuracy to predict good neurological recovery with an AUC of 0.78. In the multivariate model which also included corneal reflexes and a favorable EEG pattern this parameter remained an independent predictor (AUC for the model=0.96; false positive rate=27%). This model provided a slightly more accurate prediction compared to a combination of the commonly used variables of EEG, SSEP and clinical examination (p=0.04) (Figure1). Conclusions: The average ADC value in the postcentral cortex improves the prediction of good neurological recovery in patients post-cardiac arrest with a remaining false positive rate of 27%. (Figure Presented).

21. Jakobsen JC Lange T, Cronberg T, Lilja G, Levin H, Belohlavek J, Callaway C, Cariou A, Erlinge D, Hovdenes J, Joannidis M, Nordberg P, Oddo M, Pelosi P, Kirkegaard H, Eastwood G, Rylander C, Saxena M, Storm C, Taccone FS, Wise MP, Morgan MPG, Young P, Nichol A, Friberg H, Ullen S, Nielsen N, D. J. (2020). **Targeted hypothermia versus targeted normothermia after out-of-hospital cardiac arrest: a statistical analysis plan**. *Trials*, *21*(1). Retrieved from http://ovidsp.ovid.com/ovidweb.cgi?T=JS&PAGE=reference&D=cctr&NEWS=N&AN=CN-02191649
Background: To date, targeted temperature management (TTM) is the only neuroprotective intervention after resuscitation from cardiac arrest that is recommended by guidelines. The evidence on the effects of TTM is unclear. Methods/design: The Targeted Hypothermia Versus Targeted Normothermia After Out-of-hospital Cardiac Arrest (TTM2) trial is an international, multicentre, parallel group, investigator-initiated, randomised, superiority trial in which TTM with a target temperature of 33 [degrees]C after cardiac arrest will be compared with a strategy to maintain normothermia and active treatment of fever (>= 37.8 [degrees]C). Prognosticators, outcome assessors, the steering group, the trial coordinating team, and trial statisticians will be blinded to treatment allocation. The primary outcome will be all-cause mortality at 180 days after randomisation. We estimate a 55% mortality in the targeted normothermia group. To detect an absolute risk reduction of 7.5% with an alpha of 0.05 and 90% power, 1900 participants will be enrolled. The secondary neurological outcome will be poor functional outcome (modified Rankin scale 4-6) at 180 days after cardiac arrest. In this paper, a detailed statistical analysis plan is presented, including a comprehensive description of the statistical analyses, handling of missing data, and assessments of underlying statistical assumptions. Final analyses will be conducted independently by two qualified statisticians following the present plan. Discussion: This SAP, which was prepared before completion of enrolment, should increase the validity of the TTM trial by mitigation of analysis-bias.

22. **First-in-human study of the safety, tolerability, pharmacokinetics and-preliminary dynamics of neuroprotectant 2-iminobiotin in healthy subjects**. (2020). *First-in-Human Study of the Safety, Tolerability, Pharmacokinetics and-Preliminary Dynamics of Neuroprotectant 2-Iminobiotin in Healthy Subjects*, *15*(2), 152. Retrieved

from http://ovidsp.ovid.com/ovidweb.cgi?T=JS&PAGE=reference&D=cctr&NEWS=N&AN=CN-02183332
Background: 2-iminobiotin (2-IB) is an investigational neuroprotective agent in development for the reduction of brain cell injury after cerebral hypoxia-ischemia. Objective: The present first-in-human study evaluated the safety, tolerability, pharmacokinetics (PK) and-dynamics (PD) of 2-IB in healthy male subjects, intravenously infused with or without Captisol(R) as a solubilizing agent. Methods: This randomized, double-blind, placebo-controlled, dose-escalation study was executed in 2 groups of 9 healthy male subjects. A single dose of 2-IB 0.6 mg/kg or placebo was infused over periods between 15 min and 4 h, and repeated doses escalating from 0.6 mg/kg to 12 mg/kg, or placebo were infused every 4 h for 6 administrations in total. Results: Single and multiple doses of 2-IB up to 6 doses of 6 mg/kg with and without Captisol(R) were safe and well-tolerated in healthy male subjects. 2-IB proved to be a high-clearance drug with a volume of distribution slightly exceeding total body water volume, and with linear PK that appeared not to be affected by the presence of Captisol(R). Conclusion: Sulfobutyletherbeta-cyclodextrin (SBECD) in Captisol(R) had a low-clearance profile with a small volume of distribution, with time-independent PK. Preliminary PD characterization of repeated iv dosing of 2-IB in an acute peripheral hypoxic ischemia model in healthy subjects did not reveal any notable effects of 2-IB, noting that this model was not selected to guide efficacy in the currently pursued indication of cerebral hypoxia-ischemia.

23. Grand J Skrifvars MB, Tiainen M, Grejs AM, Jeppesen AN, Duez CHV, Rasmussen BS, Laitio T, Nee J, Taccone F, Soreide E, Kirkegaard H, H. C. (2020). **Haemodynamics and vasopressor support during prolonged targeted temperature management for 48 hours after out-of-hospital cardiac arrest: a post hoc substudy of a randomised clinical trial**. *European Heart Journal: Acute Cardiovascular Care*. Retrieved from http://ovidsp.ovid.com/ovidweb.cgi?T=JS&PAGE=reference&D=cctr&NEWS=N&AN=CN-02140529
Background: Comatose patients admitted after out-of-hospital cardiac arrest frequently experience haemodynamic instability and anoxic brain injury. Targeted temperature management is used for neuroprotection; however, targeted temperature management also affects patients' haemodynamic status. This study assessed the haemodynamic status of out-of-hospital cardiac arrest survivors during prolonged (48 hours) targeted temperature management at 33[degrees]C. Methods: Analysis of haemodynamic and vasopressor data from 311 patients included in a randomised, clinical trial conducted in 10 European hospitals (the TTH48 trial). Patients were randomly allocated to targeted temperature management at 33[degrees]C for 24 (TTM24) or 48 (TTM48) hours. Vasopressor and haemodynamic data were reported hourly for 72 hours after admission. Vasopressor load was calculated as norepinephrine ([micro]g/kg/min) plus dopamine([micro]g/kg/min/100) plus epinephrine ([micro]g/kg/min). Results: After 24 hours, mean arterial pressure (mean+/-SD) was 74+/-9 versus 75+/-9 mmHg (P=0.19), heart rate was 57+/-16 and 55+/-14 beats/min (P=0.18), vasopressor load was 0.06 (0.03-0.15) versus 0.08 (0.03-0.15) [micro]g/kg/min (P=0.22) for the TTM24 and TTM48 groups, respectively. From 24 to 48 hours, there was no difference in mean arterial pressure (Pgroup=0.32) or lactate (Pgroup=0.20), while heart rate was significantly lower (average difference 5 (95% confidence interval 2-8) beats/min, Pgroup<0.0001) and vasopressor load was significantly higher in the TTM48 group (Pgroup=0.005). In a univariate Cox regression model, high vasopressor load was associated with mortality in univariate analysis (hazard ratio 1.59 (1.05-2.42) P=0.03), but not in multivariate analysis (hazard ratio 0.77 (0.46-1.29) P=0.33). Conclusions: In this study, prolonged targeted temperature management at 33[degrees]C for 48 hours was associated with higher vasopressor requirement but no sign of any detrimental haemodynamic effects.

24. **Low versus high blood pressure targets after out-of-Hospital cardiac arrest**. (2020). *Low versus High Blood Pressure Targets after Out-of-Hospital Cardiac Arrest*, *140*. Retrieved from http://ovidsp.ovid.com/ovidweb.cgi?T=JS&PAGE=reference&D=cctr&NEWS=N&AN=CN-02136786
Introduction: The optimal level of blood pressure after out-of-hospital cardiac arrest (OHCA) is unknown. Hypotension may aggravate cerebral hypoperfusion exacerbating the post-anoxic brain injury. On the other hand, excessive vasopressor support may increase myocardial oxygen consumption and induce arrhythmias. We aimed to evaluate the effects of different blood pressure targets on the extent of brain injury and neurological outcome in patients resuscitated from OHCA. Methods: We performed a pooled post hoc analysis of OHCA patients randomised in the Neuroprotect (NCT02541591) and COMACARE (NCT02698917) trials to either mean arterial pressure (MAP) 65 mmHg or 80/85-100 mmHg targets for the first 36 h after ICU admission. We compared the serum neuron-specific enolase (NSE) concentrations between the groups at 24, 48 and 72 h after cardiac arrest and the neurological outcome according to the Cereberal Performance Category (CPC) scale at 6 months. We defined CPC 1-2 as good outcome and CPC 3-5 as poor outcome. In addition, we conducted a two-way analysis of variance to assess the effects of the MAP target and previous chronic hypertension on NSE concentrations. Results: All 224 patients included in the original studies were included in the analysis. Of these, 111 patients were randomised to the MAP 80/85-100 mmHg group and 113 patients to the MAP 65 mmHg group. Patients assigned to the higher MAP target had significantly higher blood pressure levels (p<0.001). We did not find any statistically significant difference in NSE concentrations (Figure 1) or good neurological outcome (50% in the lower MAP group vs. 56% in the higher MAP group, p=0.417) between the intervention groups. We did not observe statistically significant interaction between the MAP target and chronic hypertension for NSE (p=0.437). Conclusion: Targeting MAP 65 mmHg vs. MAP 80/85-100 mmHg after OHCA did not affect the extent of brain injury as determined by NSE concentration or neurological outcome at 6 months. .

25. Marquez, A. M., Morgan, R. W., Landis, W. P., McManus, M. J., Starr, J., Roberts, A. L., … Karlsson, M. (2020).

Literature Search Results

Oxygen exposure during cardiopulmonary resuscitation is associated with cerebral oxidative injury in a randomized, blinded, controlled, preclinical trial. *Journal of the American Heart Association*, *9*(9), e015032. https://doi.org/http://dx.doi.org/10.1161/JAHA.119.015032
BACKGROUND: Hyperoxia during cardiopulmonary resuscitation (CPR) may lead to oxidative injury from mitochondrial-derived reactive oxygen species, despite guidelines recommending 1.0 inspired oxygen during CPR. We hypothesized exposure to 1.0 inspired oxygen during CPR would result in cerebral hyperoxia, higher mitochondrial-derived reactive oxygen species, increased oxidative injury, and similar survival compared with those exposed to 21% oxygen. METHODS AND RESULTS: Four-week-old piglets (n=25) underwent asphyxial cardiac arrest followed by randomization and blinding to CPR with 0.21 (n=10) or 1.0 inspired oxygen (n=10) through 10 minutes post return of spontaneous circulation. Sham was n=5. Survivors received 4 hours of protocolized postarrest care, whereupon brain was obtained for mitochondrial analysis and neuropathology. Groups were compared using Kruskal-Wallis test, Wilcoxon rank-sum test, and generalized estimating equations regression models. Both 1.0 and 0.21 groups were similar in systemic hemodynamics and cerebral blood flow, as well as survival (8/10). The 1.0 animals had relative cerebral hyperoxia during CPR and immediately following return of spontaneous circulation (brain tissue oxygen tension, 85% [interquartile range, 72%-120%] baseline in 0.21 animals versus 697% [interquartile range, 515%-721%] baseline in 1.0 animals; P=0.001 at 10 minutes postarrest). Cerebral mitochondrial reactive oxygen species production was higher in animals treated with 1.0 compared with 0.21 (P<0.03). Exposure to 1.0 oxygen led to increased cerebral oxidative injury to proteins and lipids, as evidenced by significantly higher protein carbonyls and 4-hydroxynoneals compared with 0.21 (P<0.05) and sham (P<0.001). CONCLUSION(S): Exposure to 1.0 inspired oxygen during CPR caused cerebral hyperoxia during resuscitation, and resultant increased mitochondrial-derived reactive oxygen species and oxidative injury following cardiac arrest.Copyright © 2020, American Heart Association Inc.. All rights reserved.

26. Zhang, S., Moiz, B., Lachance, B. B., & Jia Xiaofeng; ORCID: http://orcid.org/0000-0003-1445-8525, X. A. O.-J. (2020). **Optimizing stem cell therapy after ischemic brain injury**. *Journal of Stroke*, *22*(3), 286–305. https://doi.org/http://dx.doi.org/10.5853/jos.2019.03048
Stem cells have been used for regenerative and therapeutic purposes in a variety of diseases. In ischemic brain injury, preclinical studies have been promising, but have failed to translate results to clinical trials. We aimed to explore the application of stem cells after ischemic brain injury by focusing on topics such as delivery routes, regeneration efficacy, adverse effects, and in vivo potential optimization. PUBMED and Web of Science were searched for the latest studies examining stem cell therapy applications in ischemic brain injury, particularly after stroke or cardiac arrest, with a focus on studies addressing delivery optimization, stem cell type comparison, or translational aspects. Other studies providing further understanding or potential contributions to ischemic brain injury treatment were also included. Multiple stem cell types have been investigated in ischemic brain injury treatment, with a strong literature base in the treatment of stroke. Studies have suggested that stem cell administration after ischemic brain injury exerts paracrine effects via growth factor release, blood-brain barrier integrity protection, and allows for exosome release for ischemic injury mitigation. To date, limited studies have investigated these therapeutic mechanisms in the setting of cardiac arrest or therapeutic hypothermia. Several delivery modalities are available, each with limitations regarding invasiveness and safety outcomes. Intranasal delivery presents a potentially improved mechanism, and hypoxic conditioning offers a potential stem cell therapy optimization strategy for ischemic brain injury. The use of stem cells to treat ischemic brain injury in clinical trials is in its early phase; however, increasing preclinical evidence suggests that stem cells can contribute to the down-regulation of inflammatory phenotypes and regeneration following injury. The safety and the tolerability profile of stem cells have been confirmed, and their potent therapeutic effects make them powerful therapeutic agents for ischemic brain injury patients.Copyright © 2020 Korean Stroke Society.

27. Pickell, Z., Williams, A. M., Alam, H. B., & Hsu, C. H. (2020). **Histone deacetylase inhibitors: A novel strategy for neuroprotection and cardioprotection following ischemia/ reperfusion injury**. *Journal of the American Heart Association*, *9*(11), e016349. https://doi.org/http://dx.doi.org/10.1161/JAHA.120.016349
Ischemia/reperfusion injury is a complex molecular cascade that causes deleterious cellular damage and organ dysfunction. Stroke, sudden cardiac arrest, and acute myocardial infarction are the most common causes of ischemia/rep-erfusion injury without effective pharmacologic therapies. Existing preclinical evidence suggests that histone deacetylase inhibitors may be an efficacious, affordable, and clinically feasible therapy that can improve neurologic and cardiac outcomes following ischemia/reperfusion injury. In this review, we discuss the pathophysiology and epigenetic modulations of ischemia/ reperfusion injury and focus on the neuroprotective and cardioprotective effects of histone deacetylase inhibitors. We also summarize the protective effects of histone deacetylase inhibitors for other vital organs and highlight the key research priorities for their successful translation to the bedside.Copyright © 2020 The Authors.

28. Seidel-Effenberg, I., Scheper, V., Lenarz, T., Paasche, G., & Stover Verena; ORCID: http://orcid.org/0000-0001-8618-8793, T. A. O.-S. (2020). **Consecutive treatment with brain-derived neurotrophic factor and electrical stimulation has a protective effect on primary auditory neurons**. *Brain Sciences*, *10*(8), 1–13. https://doi.org/http://dx.doi.org/10.3390/brainsci10080559
Degeneration of neurons, such as the inner ear spiral ganglion neurons (SGN), may be decelerated or even stopped by neurotrophic factor treatment, such as brain-derived neurotrophic factor (BDNF), as well as electrical stimulation (ES). In

a clinical setting, drug treatment of the SGN could start directly during implantation of a cochlear implant, whereas electrical stimulation begins days to weeks later. The present study was conducted to determine the effects of consecutive BDNF and ES treatments on SGN density and electrical responsiveness. An electrode drug delivery device was implanted in guinea pigs 3 weeks after deafening and five experimental groups were established: two groups received intracochlear infusion of artificial perilymph (AP) or BDNF; two groups were treated with AP respectively BDNF in addition to ES (AP + ES, BDNF + ES); and one group received NF from the day of implantation until day 34 followed by ES (BDNF-ES). Electrically evoked auditory brainstem responses were recorded. After one month of treatment, the tissue was harvested and the SGN density was assessed. The results show that consecutive treatment with BDNF and ES was as successful as the simultaneous combined treatment in terms of enhanced SGN density compared to the untreated contralateral side but not in regard to the numbers of protected cells.Copyright © 2020 by the authors. Licensee MDPI, Basel, Switzerland.

29. Pichl, T., Keller, T., Hunseler, C., Roth, B., Janoschek, R., Appel, S., & Hucklenbruch-Rother, E. (2020). **Effects of ketamine on neurogenesis, extracellular matrix homeostasis and proliferation in hypoxia-exposed HT22 murine hippocampal neurons**. *Biomedical Reports*, *13*(4), 1–8. https://doi.org/http://dx.doi.org/10.3892/br.2020.1330
Ketamine is a widely used drug in pediatric anesthesia, and both neurotoxic and neuroprotective effects have been associated with its use. There are only a few studies to date which have examined the effects of ketamine on neurons under hypoxic conditions, which may lead to severe brain damage and poor neurocognitive outcomes in neonates. In the present study, the effects of ketamine on cellular pathways associated with neuro-genesis, extracellular matrix homeostasis and proliferation were examined in vitro in hypoxia-exposed neurons. Differentiated HT22 murine hippocampal neurons were treated with 1, 10 and 20 microM ketamine and cultured under hypoxic or normoxic conditions for 24 h followed by quantitative PCR analysis of relevant candidate genes. Ketamine treatment did not exert any notable effects on the mRNA expression levels of markers of neurogenesis (neuronal growth factor and syndecan 1), extracellular matrix homeostasis (matrix-metalloproteinase 2 and 9, tenascin C and tenascin R) or proliferation markers (Ki67 and proliferating cell nuclear antigen) compared with the respective untreated controls. However, there was a tendency towards downregula-tion of multiple cellular markers under hypoxic conditions and simultaneous ketamine treatment. No dose-dependent association was found in the ketamine treated groups for genetic markers of neurogenesis, extracellular matrix homeostasis or proliferation. Based on the results, ketamine may have increased the vulner-ability of hippocampal neurons in vitro to hypoxia, independent of the dose. The results of the present study contribute to the ongoing discussion on the safety concerns around ketamine use in pediatric clinical practice from a laboratory perspective.Copyright © 2020, Spandidos Publications. All rights reserved.

30. Muneer Ather; ORCID: http://orcid.org/0000-0001-9632-8470, A. A. O.-M. (2020). **Kynurenine Pathway of Tryptophan Metabolism in Neuropsychiatric Disorders: Pathophysiologic and Therapeutic Considerations**. *Clinical Psychopharmacology and Neuroscience*, *18*(4), 507–526. https://doi.org/http://dx.doi.org/10.9758/CPN.2020.18.4.507
Under physiological conditions 95% of the ingested essential amino acid tryptophan is metabolized by the kynurenine pathway (KP) to yield the ubiquitous co-enzyme nicotinamide adenine dinucleotide, fulfilling cellular energy requirements. Importantly, the intermediaries of KP exert crucial effects throughout the body, including the central nervous system. Besides, KP metabolites are implicated in diverse disease processes such as inflammation/immune disorders, endocrine/metabolic conditions, cancers and neuropsychiatric diseases. A burgeoning body of research indicates that the KP plays a pathogenic role in major psychiatric diseases like mood disorders and schizophrenia. Triggered by inflammatory processes, the balance between neurotoxic and neuroprotective branches of the KP is disturbed. In preclinical models these discrepancies result in behaviors reminiscent of depression and psychosis. In clinical samples, recent studies are discovering key kynurenine pathway abnormalities which incriminate it in the pathogenesis of the main psychiatric disorders. Harnessing this knowledge has the potential to find disease biomarkers helpful in identifying and prognosticating neuropsychiatric disorders. Concurrently, earnest research efforts directed towards manipulating the KP hold the promise of discovering novel pharmacological agents that have therapeutic value. In this manuscript, an in-depth appraisal of the extant literature is done to understand the working of KP as this applies to neuropsychiatric disorders. It is concluded that this pathway plays an overarching role in the development of major psychiatric disorders, the KP metabolites have the potential to serve as disease markers and new medications based on KP modulation can bring lasting cures for patients suffering from these intractable conditions.Copyright © 2020, Korean College of Neuropsychopharmacology

31. Fowler, M. J., Cotter, J. D., Knight, B. E., Sirianni, R. W., Sevick-Muraca, E. M., & Sandberg, D. I. (2020). **Intrathecal drug delivery in the era of nanomedicine**. *Advanced Drug Delivery Reviews*, *165–166*, 77–95. https://doi.org/http://dx.doi.org/10.1016/j.addr.2020.02.006
Administration of substances directly into the cerebrospinal fluid (CSF) that surrounds the brain and spinal cord is one approach that can circumvent the blood-brain barrier to enable drug delivery to the central nervous system (CNS). However, molecules that have been administered by intrathecal injection, which includes intraventricular, intracisternal, or lumbar locations, encounter new barriers within the subarachnoid space. These barriers include relatively high rates of turnover as CSF clears and potentially inadequate delivery to tissue or cellular targets. Nanomedicine could offer a

solution. In contrast to the fate of freely administered drugs, nanomedicine systems can navigate the subarachnoid space to sustain delivery of therapeutic molecules, genes, and imaging agents within the CNS. Some evidence suggests that certain nanomedicine agents can reach the parenchyma following intrathecal administration. Here, we will address the preclinical and clinical use of intrathecal nanomedicine, including nanoparticles, microparticles, dendrimers, micelles, liposomes, polyplexes, and other colloidalal materials that function to alter the distribution of molecules in tissue. Our review forms a foundational understanding of drug delivery to the CSF that can be built upon to better engineer nanomedicine for intrathecal treatment of disease.Copyright © 2020 The Authors

32. Rosenbaum, M. I., Clemmensen, L. S., Stromgaard, K., Bredt, D. S., & Bettler Mette Ishoy; ORCID: http://orcid.org/0000-0003-0837-0965 AO - Clemmensen, Louise S.; ORCID: http://orcid.org/0000-0001-5313-2398 AO - Stromgaard, Kristian; ORCID: http://orcid.org/0000-0003-2206-4737 AO - Bettler, Bernhard; ORCID: http://orcid.org/0000-0003-0842-8207, B. A. O.-R. (2020). **Targeting receptor complexes: a new dimension in drug discovery**. *Nature Reviews Drug Discovery*, *19*(12), 884–901. https://doi.org/http://dx.doi.org/10.1038/s41573-020-0086-4
Targeting receptor proteins, such as ligand-gated ion channels and G protein-coupled receptors, has directly enabled the discovery of most drugs developed to modulate receptor signalling. However, as the search for novel and improved drugs continues, an innovative approach - targeting receptor complexes - is emerging. Receptor complexes are composed of core receptor proteins and receptor-associated proteins, which have profound effects on the overall receptor structure, function and localization. Hence, targeting key protein-protein interactions within receptor complexes provides an opportunity to develop more selective drugs with fewer side effects. In this Review, we discuss our current understanding of ligand-gated ion channel and G protein-coupled receptor complexes and discuss strategies for their pharmacological modulation. Although such strategies are still in preclinical development for most receptor complexes, they exemplify how receptor complexes can be drugged, and lay the groundwork for this nascent area of research.Copyright © 2020, Springer Nature Limited.

33. Wollborn, J., Schallner, N., Buerkle, H., Schick, M. A., Goebel, U., Steiger, C., … Kari, F. A. (2020). **Carbon Monoxide Exerts Functional Neuroprotection After Cardiac Arrest Using Extracorporeal Resuscitation in Pigs**. *Critical Care Medicine*, *48*(4), e299–e307. https://doi.org/http://dx.doi.org/10.1097/CCM.0000000000004242
OBJECTIVES: Neurologic damage following cardiac arrest remains a major burden for modern resuscitation medicine. Cardiopulmonary resuscitation with extracorporeal circulatory support holds the potential to reduce morbidity and mortality. Furthermore, the endogenous gasotransmitter carbon monoxide attracts attention in reducing cerebral injury. We hypothesize that extracorporeal resuscitation with additional carbon monoxide application reduces neurologic damage. DESIGN: Randomized, controlled animal study. SETTING: University research laboratory. SUBJECTS: Landrace-hybrid pigs. INTERVENTIONS: In a porcine model, carbon monoxide was added using a novel extracorporeal releasing system after resuscitation from cardiac arrest. MEASUREMENTS AND MAIN RESULTS: As markers of cerebral function, neuromonitoring modalities (somatosensory-evoked potentials, cerebral oximetry, and transcranial Doppler ultrasound) were used. Histopathologic damage and molecular markers (caspase-3 activity and heme oxygenase-1 expression) were analyzed. Cerebral oximetry showed fast rise in regional oxygen saturation after carbon monoxide treatment at 0.5 hours compared with extracorporeal resuscitation alone (regional cerebral oxygen saturation, 73% +/- 3% vs 52% +/- 8%; $p < 0.05$). Median nerve somatosensory-evoked potentials showed improved activity upon carbon monoxide treatment, whereas post-cardiac arrest cerebral perfusion differences were diminished. Histopathologic damage scores were reduced compared with customary resuscitation strategies (hippocampus: sham, 0.4+/-0.2; cardiopulmonary resuscitation, 1.7+/-0.4; extracorporeal cardiopulmonary resuscitation, 2.3+/-0.2; extracorporeal cardiopulmonary resuscitation with carbon monoxide application [CO-E-CPR], 0.9+/-0.3; $p < 0.05$). Furthermore, ionized calcium-binding adaptor molecule 1 staining revealed reduced damage patterns upon carbon monoxide treatment. Caspase-3 activity (cardiopulmonary resuscitation, 426+/-169 pg/mL; extracorporeal cardiopulmonary resuscitation, 240+/-61 pg/mL; CO-E-CPR, 89+/-26 pg/mL; $p < 0.05$) and heme oxygenase-1 (sham, 1+/-0.1; cardiopulmonary resuscitation, 2.5+/-0.4; extracorporeal cardiopulmonary resuscitation, 2.4+/-0.2; CO-E-CPR, 1.4+/-0.2; $p < 0.05$) expression were reduced after carbon monoxide exposure. CONCLUSION(S): Carbon monoxide application during extracorporeal resuscitation reduces injury patterns in neuromonitoring and decreases histopathologic cerebral damage by reducing apoptosis. This …

34. Wudayagiri, R., & Chintha, V. (2020). **Isolation and neuroprotective prospective of novel bioactive compound "3-(3,4-dimethoxyphenyl)-1-(4-methoxyphenyl) prop-2-en-1-one" against ketamine-induced cognitive deficits in schizophrenia: an experimental study**. *Natural Product Research*. https://doi.org/http://dx.doi.org/10.1080/14786419.2020.1869968
For the first time a new flavonoid compound is isolated from the seeds of Celastrus paniculatus (CP) using different chromatographic techniques and it's structure is predicted as "3-(3,4-dimethoxyphenyl)-1-(4-methoxyphenyl)prop-2-en-1-one" by employing various spectroscopic studies. The neuroprotective potential of this flavonoid was evaluated against ketamine-induced cognitive deficits with special reference to cholinergic system in vivo. The compound has exhibited significant neuroprotective property against ketamine-induced cholinergic alterations in different brain regions of rat which are restored to normal during the treatment with the compound on par with the reference compound, clozapine. Moreover, the isolated compound was found to be non-toxic to the animal during the treatment which indicates its safety in any human health related applications and can add value to the new drug development. In conclusion, this is

the first study of new flavonoid compound of CP and its protective efficacy against schizophrenia.Copyright © 2020 Informa UK Limited, trading as Taylor & Francis Group.

35. Qi, A., Cao, Y., & Wang Aizhong; ORCID: http://orcid.org/0000-0001-5700-705X, A. A. O.-W. (2020). **Ketamine and Propofol Protect Neuron Cells from Oxygen-Glucose Deprivation-Induced Injury through SAPK/JNK Signalling Pathway**. *BioMed Research International*, *2020*, 8868839. https://doi.org/http://dx.doi.org/10.1155/2020/8868839
Ketamine and propofol are commonly used anaesthetic reagents. Recent research revealed that ketamine and propofol have an important role in cell survival. However, it remains unknown whether they affect the outcome of hypoxic-ischemic brain injury. To address this issue, we in this study investigated the effects of ketamine and propofol on the survival and proliferation of neuronal PC12 cells after exposure to oxygen-glucose deprivation- (OGD-) induced injury. PC12 cells were maintained under a 3-dimensional (3D) culture system to mimic a real physiological microenvironment. The cell injury was induced by 5% CO2 and 95% N2 for a different time point. MTT assay was used for the cell proliferation assay. The cell apoptosis was evaluated by annexin V and propidium iodide (PI) labeling, immunofluorescence staining, transmission electron microscopy (TEM), flow cytometry, and Western blot, respectively. Our results showed that PC12 cell apoptosis was significantly increased for up to 70% after the cells were treated with OGD for 24 hours and reduced to baseline at the 72-hour time point. However, pretreatment with ketamine and propofol significantly protected the cells from OGD-induced cell apoptosis, as evidenced by more expression of antiapoptotic Bcl-2 and lower expression of proapoptotic cleaved caspase-3, phosphor-SAPK/JNK, and phosphor-c-Jun than those of untreated control cells. Thus, we conclude that ketamine and propofol protected PC12 cells from OGD-induced cell apoptosis, at least partially through the SAPK/JNK signalling pathway.Copyright © 2020 Aihua Qi et al.

36. O'Brien, C. E., Santos, P. T., Kulikowicz, E., Koehler, R. C., Lee, J. K., & Martin Caitlin E.; ORCID: http://orcid.org/0000-0002-5609-1955, L. J. A. O.-O. (2020). **Neurologic effects of short-term treatment with a soluble epoxide hydrolase inhibitor after cardiac arrest in pediatric swine**. *BMC Neuroscience*, *21*(1), 43. https://doi.org/http://dx.doi.org/10.1186/s12868-020-00596-y
Background: Cardiac arrest (CA) is the most common cause of acute neurologic insult in children. Many survivors have significant neurocognitive deficits at 1 year of recovery. Epoxyeicosatrienoic acids (EETs) are multifunctional endogenous lipid signaling molecules that are involved in brain pathobiology and may be therapeutically relevant. However, EETs are rapidly metabolized to less active dihydroxyeicosatrienoic acids by soluble epoxide hydrolase (sEH), limiting their bioavailability. We hypothesized that sEH inhibition would improve outcomes after CA in an infant swine model. Male piglets (3-4 kg, 2 weeks old) underwent hypoxic-asphyxic CA. After resuscitation, they were randomized to intravenous treatment with an sEH inhibitor (TPPU, 1 mg/kg; n = 8) or vehicle (10% poly(ethylene glycol); n = 9) administered at 30 min and 24 h after return of spontaneous circulation. Two sham-operated groups received either TPPU (n = 9) or vehicle (n = 8). Neurons were counted in hematoxylin- and eosin-stained sections from putamen and motor cortex in 4-day survivors. Result(s): Piglets in the CA + vehicle groups had fewer neurons than sham animals in both putamen and motor cortex. However, the number of neurons after CA did not differ between vehicle- and TPPU-treated groups in either anatomic area. Further, 20% of putamen neurons in the Sham + TPPU group had abnormal morphology, with cell body attrition and nuclear condensation. TPPU treatment also did not reduce neurologic deficits. Conclusion(s): Treatment with an sEH inhibitor at 30 min and 24 h after resuscitation from asphyxic CA does not protect neurons or improve acute neurologic outcomes in piglets.Copyright © 2020, The Author(s).

37. Kumar, V. H. S., Gugino, S., Nielsen, L., Chandrasekharan, P., Koenigsknecht, C., Helman, J., & Lakshminrusimha Vasantha H. S.; ORCID: http://orcid.org/0000-0003-0950-2608, S. A. O.-K. (2020). **Protection from systemic pyruvate at resuscitation in newborn lambs with asphyxial cardiac arrest**. *Physiological Reports*, *8*(12), e14472. https://doi.org/http://dx.doi.org/10.14814/phy2.14472
Background: Infants with hypoxic-ischemic injury often require cardiopulmonary resuscitation. Mitochondrial failure to generate adenosine triphosphate (ATP) during hypoxic-ischemic reperfusion injury contributes to cellular damage. Current postnatal strategies to improve outcome in hypoxic-ischemic injury need sophisticated equipment to perform servo-controlled cooling. Administration of intravenous pyruvate, an antioxidant with favorable effects on mitochondrial bioenergetics, is a simple intervention that can have a global impact. We hypothesize that the administration of pyruvate following the return of spontaneous circulation (ROSC) would improve cardiac function, systemic hemodynamics, and oxygen utilization in the brain in newborn lambs with cardiac arrest (CA). Method(s): Term lambs were instrumented, delivered by C-section and asphyxia induced by umbilical cord occlusion along with clamping of the endotracheal tube until asystole; Lambs resuscitated following 5 min of CA; upon ROSC, lambs were randomized to receive pyruvate or saline infusion over 90 min and ventilated for 150 min postinfusion. Pulmonary and systemic hemodynamics and arterial gases monitored. We measured plasma pyruvate, tissue lactate, and ATP levels (heart and brain) in both groups. Result(s): Time to ROSC was not different between the two groups. Systolic and diastolic blood pressures, stroke volume, arterial oxygen content, and cerebral oxygen delivery were similar between the two groups. The cerebral metabolic rate of oxygen was higher following pyruvate infusion; higher oxygen consumption in the brain was associated with lower plasma levels but higher brain ATP levels compared to the saline group. Conclusion(s): Pyruvate promotes energy generation accompanied by efficient oxygen utilization in the brain and may facilitate additional neuroprotection in the presence of hypoxic-ischemic injury.Copyright © 2020 The Authors. Physiological Reports published by Wiley

Periodicals, Inc. on behalf of The Physiological Society and the American Physiological Society.

38. Kapoor, M. C. (2020). **Neurological dysfunction after cardiac surgery and cardiac intensive care admission: A narrative review part 1: The problem; Nomenclature; delirium and postoperative neurocognitive disorder; and the role of cardiac surgery and anesthesia**. *Annals of Cardiac Anaesthesia*, *23*(4), 383–390. https://doi.org/http://dx.doi.org/10.4103/aca.ACA_138_19
The association with cardiac surgery with cognitive decline was first reported in the 1960s after the introduction of coronary artery surgery. The incidence in cognitive decline was thought to be more after cardiac surgery, especially with the use of the cardiopulmonary bypass. Anesthesia and surgery are both associated with cognitive decline but many other factors appear to contribute its genesis. On-pump surgery, microembolization during manipulation of the heart and great vessels, temperature changes, pH changes, and altered cerebral perfusion, during cardiac surgery, have all been blamed for this. Postoperative cognitive decline is associated with poor clinical outcomes and higher mortality. Several studies have been conducted in the last decade to determine the genesis of this malady. Current evidence is absolving cardiac surgery and anesthesia to be the primary causes per se of cognitive dysfunction.Copyright © 2020 Annals of Cardiac Anaesthesia Published by Wolters Kluwer - Medknow.

39. Friberg, H., Paidas, M. J., Lorenzo, J., & Deye, N. (2020). **Unique Uses of Cooling Strategies**. *Therapeutic Hypothermia and Temperature Management*, *10*(3), 131–134. https://doi.org/http://dx.doi.org/10.1089/ther.2020.29076.hjf

40. Zhang, D., Zhao, X., Li, L., Lu, Y., & Zhang, Q. (2020). **Aerobic exercise attenuates neurodegeneration and promotes functional recovery - Why it matters for neurorehabilitation & neural repair**. *Neurochemistry International*, *141*, 104862. https://doi.org/http://dx.doi.org/10.1016/j.neuint.2020.104862
Aerobic exercise facilitates optimal neurological function and exerts beneficial effects in neurologic injuries. Both animal and clinical studies have shown that aerobic exercise reduces brain lesion volume and improves multiple aspects of cognition and motor function after stroke. Studies using animal models have proposed a wide range of potential molecular mechanisms that underlie the neurological benefits of aerobic exercise. Furthermore, additional exercise parameters, including time of initiation, exercise dosage (exercise duration and intensity), and treatment modality are also critical for clinical application, as identifying the optimal combination of parameters will afford patients with maximal functional gains. To clarify these issues, the current review summarizes the known neurological benefits of aerobic exercise under both physiological and pathological conditions and then considers the molecular mechanisms underlying these benefits in the contexts of stroke-like focal cerebral ischemia and cardiac arrest-induced global cerebral ischemia. In addition, we explore the key roles of exercise parameters on the extent of aerobic exercise-induced neurological benefits to elucidate the optimal combination for aerobic exercise intervention. Finally, the current challenges for aerobic exercise implementation after stroke are discussed.Copyright © 2020 Elsevier Ltd

41. Alnajar, A., Aleong, E. F., Azhar, M. Z., Azarrafiy, R., & Lamelas Ahmed; ORCID: http://orcid.org/0000-0002-1205-2014 AO - Lamelas, Joseph; ORCID: http://orcid.org/0000-0003-1570-1988, J. A. O.-A. (2020). **Review of cerebral perfusion strategies for aortic surgery with application for minimally invasive approaches**. *Journal of Cardiac Surgery*, *35*(12), 3539–3544. https://doi.org/http://dx.doi.org/10.1111/jocs.15061
Aortic arch and hemiarch surgery necessitate the temporary interruption of blood perfusion to the brain. Despite its complexity, hemiarch and ascending aortic surgery can be performed via a minimally invasive approach. Due to the higher risk of neurological injury during a circulatory arrest, several techniques were developed to further protect the brain during this surgery. We searched the Embase, Medline, and Cochrane databases and identified articles reporting outcomes of antegrade and retrograde cerebral perfusion strategies. Herein, we outline surgical approaches, intra-operative technical considerations, and clinical outcomes of hemiarch and ascending aortic surgery. Hemiarch and ascending aortic surgery is associated with a higher risk of mortality and morbidity. Attention to the optimal approach and cerebral protection strategy has been shown to significantly affect outcomes and mitigate risk.Copyright © 2020 Wiley Periodicals LLC

42. Dezfulian, C., & Lavonas, E. J. (2020). **Precision Cardiac Arrest Resuscitation Based on Etiology**. *Critical Care Clinics*, *36*(4), 737–752. https://doi.org/http://dx.doi.org/10.1016/j.ccc.2020.07.005
Cardiac arrest results from a broad range of etiologies that can be broadly grouped as sudden and asphyxial. Animal studies point to differences in injury pathways invoked in the heart and brain that drive injury and outcome after these different forms of cardiac arrest. Present guidelines largely ignore etiology in their management recommendations. Existing clinical data reveal significant heterogeneity in the utility of presently employed resuscitation and postresuscitation strategies based on etiology. The development of future neuroprotective and cardioprotective therapies should also take etiology into consideration to optimize the chances for successful translation.Copyright © 2020 Elsevier Inc.

43. Korczak, M., Kurowski, P., Lesniak, A., Bujalska-Zadrozny, M., Gronbladh, A., & Filipowska, A. (2020). **GABAB receptor intracellular signaling: novel pathways for depressive disorder treatment?** *European Journal of Pharmacology*, *885*, 173531. https://doi.org/http://dx.doi.org/10.1016/j.ejphar.2020.173531

Affecting over 320 million people around the world, depression has become a formidable challenge for modern medicine. In addition, an increasing number of studies cast doubt on the monoamine theory of depressive disorder and, worryingly, antidepressant medications only significantly benefit patients with severe depression. Thus, it is not surprising that researchers have shown an increased interest in new theories attempting to explain the pathogenesis of this disease. One example is the excitatory/inhibitory transmission imbalance theory. These abnormalities involve glutamate and gamma-aminobutyric acid (GABA) signaling. Studies on GABAB receptors and their antagonists are particularly promising for the treatment of depressive disorders. In this paper, intracellular pathways controlled by GABAB receptors and their links to depression are described, including the impact of ketamine on GABAergic synaptic transmission.Copyright © 2020

44. Steinberg, A., & Elmer, J. (2020). **Postarrest Interventions that Save Lives**. *Emergency Medicine Clinics of North America*, *38*(4), 771–782. https://doi.org/http://dx.doi.org/10.1016/j.emc.2020.06.001
Patients resuscitated from cardiac arrest require complex management. An organized approach to early postarrest care can improve patient outcomes. Priorities include completing a focused diagnostic work-up to identify and reverse the inciting cause of arrest, stabilizing cardiorespiratory instability to prevent rearrest, minimizing secondary brain injury, evaluating the risk and benefits of transfer to a specialty care center, and avoiding early neurologic prognostication.Copyright © 2020 Elsevier Inc.

45. Lee Jong Woo; ORCID: http://orcid.org/0000-0001-5283-7476, J. W. A. O.-L. (2020). **Mechanistic Wager on Outcome in Coma After Cardiac Arrest: The EEG Signature in Burst Suppression Provides Some Clues**. *Epilepsy Currents*, *20*(4), 199–201. https://doi.org/http://dx.doi.org/10.1177/1535759720929289
Independent Functional Outcomes After Prolonged Coma Following Cardiac Arrest: A Mechanistic Hypothesis Forgacs PB, Devinsky O, Schiff ND. Ann Neurol. 2020;87:618-632. doi:10.1002/ana.25690. Objective(s): Survivors of prolonged (>2 weeks) post-cardiac arrest (CA) coma are expected to remain permanently disabled. We aimed to investigate 3 outlier patients who ultimately achieved independent functional outcomes after prolonged post-CA coma to identify electroencephalographic (EEG) markers of their recovery potential. For validation purposes, we also aimed to evaluate these markers in an independent cohort of post-CA patients. Method(s): We identified 3 patients with late recovery from coma (17-37 days) following CA who recovered to functionally independent behavioral levels. We performed spectral power analyses of available EEGs during prominent burst suppression patterns (BSP) present in all 3 patients. Using identical methods, we also assessed the relationship of intraburst spectral power and outcomes in a prospectively enrolled cohort of post-CA patients. We performed chart reviews of common clinical, imaging, and EEG prognostic variables and clinical outcomes for all patients. Result(s): All 3 patients with late recovery from coma lacked evidence of overwhelming cortical injury but demonstrated prominent BSP on EEG. Spectral analyses revealed a prominent theta (~4-7 Hz) feature dominating the bursts during BSP in these patients. In the prospective cohort, similar intraburst theta spectral features were evident in patients with favorable outcomes; patients with BSP and unfavorable outcomes showed either no features, transient burst features, or decreasing intraburst frequencies with time. Interpretation(s): Burst suppression patterns with theta (~4-7 Hz) peak intraburst spectral power after CA may index a recovery potential. We discuss our results in the context of optimizing metabolic substrate availability and stimulating the corticothalamic system during recovery from prolonged post-CA coma.Copyright © The Author(s) 2020.

46. Eftimiadi, G., Chiaretti, A., Buonsenso, D., Rendeli, C., Staccioli, S., & Conti, G. (2020). **Intranasal nerve growth factor administration improves neurological outcome after GBS meningitis**. *Child's Nervous System*, *36*(9), 2083–2088. https://doi.org/http://dx.doi.org/10.1007/s00381-020-04590-x
Nerve growth factor (NGF) is a neurotrophin that promotes neural recovery and plasticity after experimental brain injury, supporting neuronal growth, differentiation, and survival of brain cells. Only a few studies reported NGF administration in pediatric patients with impaired brain functions after traumatic injuries, ischemic or infectious diseases, such as meningitis. We described the beneficial therapeutic effects of human-recombinant nerve growth factor (hr-NGF) treatment in an infant with persistent unresponsive wakefulness syndrome (UWS), due to late-onset group B Streptococcus meningitis. The infant received five monthly cycles of intranasal hr-NGF (0.1 mg/kg, 3 times daily for 7 consecutive days) through a mucosal atomizer device (MAD). NGF administration improved functional [positron emission tomography/computed tomography (PET/CT), single-photon emission/computed tomography (SPECT/CT), and magnetic resonance imaging (MRI)] assessments, electrophysiological [Electroencephalogram (EEG)] studies, as well as main cognitive processes and clinical and neurological functions. After hr-NGF treatment, significant improvements in facial mimicry, attention, motor reactions, oral motility, and feeding capacity were observed. She also recovered some hypothalamic functions and her cough reflex was restored. No side effects were reported during and after the treatment. For the first time ever, hr-NGF has been successfully utilized in an infant with UWS and severe neurologic outcome due to a bacterial meningitis. Although further studies are needed for better understanding the neuroprotective role of this neurotrophin, intranasal hr-NGF administration appears to be a promising and save rescuing strategy treatment in infants with severe neurological impairment after brain damage.Copyright © 2020, Springer-Verlag GmbH Germany, part of Springer Nature.

47. Nakamura, F., Ohshima, T., & Goshima, Y. (2020). **Collapsin Response Mediator Proteins: Their Biological Functions and Pathophysiology in Neuronal Development and Regeneration**. *Frontiers in Cellular*

Neuroscience, *14*, 188. https://doi.org/http://dx.doi.org/10.3389/fncel.2020.00188
Collapsin response mediator proteins (CRMPs), which consist of five homologous cytosolic proteins, are one of the major phosphoproteins in the developing nervous system. The prominent feature of the CRMP family proteins is a new class of microtubule-associated proteins that play important roles in the whole process of developing the nervous system, such as axon guidance, synapse maturation, cell migration, and even in adult brain function. The CRMP C-terminal region is subjected to posttranslational modifications such as phosphorylation, which, in turn, regulates the interaction between the CRMPs and various kinds of proteins including receptors, ion channels, cytoskeletal proteins, and motor proteins. The gene-knockout of the CRMP family proteins produces different phenotypes, thereby showing distinct roles of all CRMP family proteins. Also, the phenotypic analysis of a non-phosphorylated form of CRMP2-knockin mouse model, and studies of pharmacological responses to CRMP-related drugs suggest that the phosphorylation/dephosphorylation process plays a pivotal role in pathophysiology in neuronal development, regeneration, and neurodegenerative disorders, thus showing CRMPs as promising target molecules for therapeutic intervention.© Copyright © 2020 Nakamura, Ohshima and Goshima.

48. Sun, Y., Wei, C., Wu, A., Cui, V., & Xiu, M. (2020). **Electroencephalography: Clinical Applications During the Perioperative Period**. *Frontiers in Medicine*, *7*, 251. https://doi.org/http://dx.doi.org/10.3389/fmed.2020.00251
Electroencephalography (EEG) monitoring has become technically feasible in daily clinical anesthesia practice. EEG is a sensitive method for detecting neurophysiological changes in the brain and represents an important frontier in the monitoring and treatment of patients in the perioperative period. In this review, we briefly introduce the essential principles of EEG. We review EEG application during anesthesia practice in the operating room, including the use of processed EEG in depth of anesthesia assessment, raw EEG monitoring in recognizing brain states under different anesthetic agents, the use of EEG in the prevention of perioperative neurocognitive disorders and detection of cerebral ischemia. We then discuss EEG utilization in the intensive care units, including the use of EEG in sedative level titration and prognostication of clinical outcomes. Existing literature provides insight into both the advances and challenges of the clinical applications of EEG. Future study is clearly needed to elucidate the precise EEG features that can reliably optimize perioperative care for individual patients.© Copyright © 2020 Sun, Wei, Cui, Xiu and Wu.

49. Ahsan, S. M., Ahsan, S. D., Khalid, O., & Agha Hina; ORCID: http://orcid.org/0000-0002-4451-798X, H. A. O.-A. (2020). **Usefulness of vortioxetine noted in depression secondary to hypoxic brain injury and residual cognitive deficits**. *Therapeutic Advances in Psychopharmacology*, *10*. https://doi.org/http://dx.doi.org/10.1177/2045125320943399
We report on our experience of treating depression secondary to hypoxic brain injury with the antidepressant vortioxetine to share in the growing body of evidence. Our patient was referred to a community mental health team with depression, amotivation and memory difficulties following a myocardial infarction and cardiac arrest 2 years prior. Regardless of motor recovery, major cognitive deficits remained; however, neurorehabilitation was impossible due to severe depression. We tried vortioxetine in the light of two failed antidepressants and saw a remarkable improvement in mood, motivation and engagement.Copyright © The Author(s), 2020.

50. Liu, J., Chandaka, G. K., Zhang, R., & Parfenova Helena; ORCID: http://orcid.org/0000-0002-8623-8305, H. A. O.-P. (2020). **Acute antioxidant and cytoprotective effects of sulforaphane in brain endothelial cells and astrocytes during inflammation and excitotoxicity**. *Pharmacology Research and Perspectives*, *8*(4), e00630. https://doi.org/http://dx.doi.org/10.1002/prp2.630
Sulforaphane (SFN), a bioactive phytochemical isothiocyanate, has a wide spectrum of cytoprotective effects that involve induction of antioxidant genes. Nongenomic antioxidant effects of SFN have not been investigated. Brain oxidative stress during inflammation and excitotoxicity leads to neurovascular injury. We tested the hypothesis that SNF exhibits acute antioxidant effects and prevents neurovascular injury during oxidative stress. In primary cultures of cerebral microvascular endothelial cells (CMVEC) and cortical astrocytes from the newborn pig brain, a pro-inflammatory cytokine TNF-alpha and an excitotoxic glutamate elevate reactive oxygen species (ROS) and cause cell death by apoptosis. Nox4 NADPH oxidase is the main Nox isoform in CMVEC and cortical astrocytes that is acutely activated by TNF-alpha and glutamate leading to ROS-mediated cell death by apoptosis. The Nox4 inhibitor GKT137831 blocked NADPH oxidase activity and overall ROS elevation, and prevented apoptosis of CMVEC and astrocytes exposed to TNF-alpha and glutamate, supporting the leading role of Nox4 in the neurovascular injury. Synthetic SFN (10-11-10-6 mol/L) inhibited NADPH oxidase activity and reduced overall ROS production in CMVEC and astrocytes within 1-hour exposure to TNF-alpha and glutamate. Furthermore, in the presence of SFN, the ability of TNF-alpha and glutamate to produce apoptosis in CMVEC and cortical astrocytes was completely prevented. Overall, SFN at low concentrations exhibits antioxidant and antiapoptotic effects in cerebral endothelial cells and cortical astrocytes via a via a nongenomic mechanism that involves inhibition of Nox4 NADPH oxidase activity. SFN may prevent cerebrovascular injury during brain oxidative stress caused by inflammation and glutamate excitotoxicity.Copyright © 2020 The Authors. Pharmacology Research & Perspectives published by British Pharmacological Society and American Society for Pharmacology and Experimental Therapeutics and John Wiley & Sons Ltd

51. Sola, P., Krishnamurthy, P., Chintamaneni, P. K., Pindiprolu, S. K. S. S., & Kumari Praveen; ORCID: http://orcid.org/0000-0003-2666-0878 AO - Chintamaneni, Pavan Kumar; ORCID: http://orcid.org/0000-0002-8655-

7680 AO - Pindiprolu, Sai Kiran S.S; ORCID: http://orcid.org/0000-0002-5400-4491, M. A. O.-K. (2020). **Novel drug delivery systems of beta2 adrenoreceptor agonists to suppress SNCA gene expression and mitochondrial oxidative stress in Parkinson's disease management**. *Expert Opinion on Drug Delivery*, *17*(8), 1119–1132. https://doi.org/http://dx.doi.org/10.1080/17425247.2020.1779218
Introduction: alpha-synuclein (SNCA), a major component of Lewy body is a pathological hallmark of Parkinson's disease (PD). Mutations in the SNCA gene cause misfolding and aggregation of SNCA protein, which results in neurodegeneration. Several studies have established the neuroprotective benefits of beta2-adrenoreceptor (beta2AR) agonists in PD However, beta2AR agonists are associated with peripheral side effects- tachycardia, palpitation, pulmonary edema, myocardial ischemia, and cardiac arrhythmia due to betaARactivation in peripheral tissues. PD therapy with beta2AR agonists, therefore, warrants a brain-specific delivery. Area covered: This review highlights the SNCA mediated neurodegenerative pathways in PD and various treatment strategies under investigation to lower SNCA gene expression, primarily focusing on beta2AR mediated pathway. The review also discusses the beneficial and side effects of beta2AR agonists in PD treatment by reviewing clinical trials, epidemiological studies, and meta-analysis data. Here we depict the need to develop a novel drug delivery system to achieve brain-specific delivery of beta2AR agonists to overcome peripheral side effects and also propose various nano delivery strategies to achieve the same. Expert opinion: Brain targeted delivery of beta2AR agonists via various nano delivery systems will significantly downregulate SNCA gene expression in PD and also overcomes peripheral side effects of beta2AR agonists.Copyright © 2020 Informa UK Limited, trading as Taylor & Francis Group.

52. Lv, B., Jiang, X.-M., Wang, D.-W., Han, D.-F., Liu, X.-L., & Chen, J. (2020). **Protective effects and mechanisms of action of ulinastatin against cerebral ischemia-reperfusion injury**. *Current Pharmaceutical Design*, *26*(27), 3340–3348. https://doi.org/http://dx.doi.org/10.2174/1381612826666200303114955
Background: Cerebral ischemia-reperfusion injury is an extremely complicated pathological process that is clinically characterized by high rates of disability and mortality. It is imperative to explore some effective neuroprotective agents for its treatment. Ulinastatin is a protease inhibitor with anti-inflammatory and antioxidant activity. For the past few years, new studies of ulinastatin for the treatment of ischemic brain injury have emerged. Objective(s): We conducted a review to summarize the mechanisms of ulinastatin and analyze its neuroprotective action against cerebral ischemia-reperfusion injury. Method(s): We reviewed and summarized pertinent reports published between 1993 and 2019 from PubMed, Web of Science, and Embaseby searching for the scientific terms ulinastatin, cerebral ischemia-reperfusion injury, neuroprotective, stroke, cardiac arrest, and brain edema. Result(s): The protective mechanisms of ulinastatin in the key steps of cerebral ischemia-reperfusion injury in-clude inhibition of inflammatory response, oxidative stress, neuronal apoptosis, neuronal autophagy, and aq-uaporin-4 expression as well as improvement in blood-brain barrier permeability. In addition, we provide a per-spective on potential research directions and clinical safety. Conclusion(s): Ulinastatin seems to have the potential to alleviate cerebral ischemia-reperfusion injury. These findings may be valuable to further promote the research and development of drug candidates and provide novel and reliable references for rational drug use.Copyright © 2020 Bentham Science Publishers.

53. Shah, V. A., & Geocadin, R. G. (2020). **Therapeutic Hypothermia in Neurocritical Care**. *Current Clinical Neurology*, 57–72. https://doi.org/http://dx.doi.org/10.1007/978-3-030-36548-6_5
The neuroprotective benefits of therapeutic hypothermia (TH) have been known for several decades but have become conventional in clinical practice only over the last decade, after large randomized clinical trials (RCT) affirmed the benefits of TH in preventing neurologic injury, predominantly in cardiac arrest survivors. In neurocritical care, the applications of TH have been extended to several critical neurologic illnesses including refractory elevation in intracranial pressure (ICP), status epilepticus, ischemic stroke, and traumatic brain injury among others. The appropriate clinical use of TH requires understanding the postulated pathophysiologic mechanisms behind TH-induced neuroprotection, associated wanted and unwanted physiologic effects of TH, methods of inducing TH, and clinical care of patients during TH. All of these aspects will be addressed in this chapter.Copyright © 2020, Springer Nature Switzerland AG.

54. Pribish, A., Wood, N., & Kalava Abby; ORCID: http://orcid.org/0000-0003-2229-7960, A. A. O.-P. (2020). **A Review of Nonanesthetic Uses of Ketamine**. *Anesthesiology Research and Practice*, *2020*, 5798285. https://doi.org/http://dx.doi.org/10.1155/2020/5798285
Ketamine, a nonselective NMDA receptor antagonist, is used widely in medicine as an anesthetic agent. However, ketamine's mechanisms of action lead to widespread physiological effects, some of which are now coming to the forefront of research for the treatment of diverse medical disorders. This paper aims at reviewing recent data on key nonanesthetic uses of ketamine in the current literature. MEDLINE, CINAHL, and Google Scholar databases were queried to find articles related to ketamine in the treatment of depression, pain syndromes including acute pain, chronic pain, and headache, neurologic applications including neuroprotection and seizures, and alcohol and substance use disorders. It can be concluded that ketamine has a potential role in the treatment of all of these conditions. However, research in this area is still in its early stages, and larger studies are required to evaluate ketamine's efficacy for nonanesthetic purposes in the general population.Copyright © 2020 Abby Pribish et al.

55. Goodfellow, M. J., Borcar, A., Proctor, J. L., Greco, T., Fiskum, G., & Rosenthal, R. E. (2020). **Transcriptional activation of antioxidant gene expression by Nrf2 protects against mitochondrial dysfunction and**

neuronal death associated with acute and chronic neurodegeneration. *Experimental Neurology*, *328*, 113247. https://doi.org/http://dx.doi.org/10.1016/j.expneurol.2020.113247
Mitochondria are both a primary source of reactive oxygen species (ROS) and a sensitive target of oxidative stress; damage to mitochondria can result in bioenergetic dysfunction and both necrotic and apoptotic cell death. These relationships between mitochondria and cell death are particularly strong in both acute and chronic neurodegenerative disorders. ROS levels are affected by both the production of superoxide and its toxic metabolites and by antioxidant defense mechanisms. Mitochondrial antioxidant activities include superoxide dismutase 2, glutathione peroxidase and reductase, and intramitochondrial glutathione. When intracellular conditions disrupt the homeostatic balance between ROS production and detoxification, a net increase in ROS and an oxidized shift in cellular redox state ensues. Cells respond to this imbalance by increasing the expression of genes that code for proteins that protect against oxidative stress and inhibit cytotoxic oxidation of proteins, DNA, and lipids. If, however, the genomic response to mitochondrial oxidative stress is insufficient to maintain homeostasis, mitochondrial bioenergetic dysfunction and release of pro-apoptotic mitochondrial proteins into the cytosol initiate a variety of cell death pathways, ultimately resulting in potentially lethal damage to vital organs, including the brain. Nuclear factor erythroid 2-related factor 2 (Nrf2) is a translational activating protein that enters the nucleus in response to oxidative stress, resulting in increased expression of numerous cytoprotective genes, including genes coding for mitochondrial and non-mitochondrial antioxidant proteins. Many experimental and some FDA-approved drugs promote this process. Since mitochondria are targets of ROS, it follows that protection against mitochondrial oxidative stress by the Nrf2 pathway of gene expression contributes to neuroprotection by these drugs. This document reviews the evidence that Nrf2 activation increases mitochondrial antioxidants, thereby protecting mitochondria from dysfunction and protecting neural cells from damage and death. New experimental results are provided demonstrating that post-ischemic administration of the Nrf2 activator sulforaphane protects against hippocampal neuronal death and neurologic injury in a clinically-relevant animal model of cardiac arrest and resuscitation.Copyright © 2020

56. Sabharwal, V., Talahma, M., Iwuchukwu, I. O., Ciccotto, G., Khandker, N., McGrade, H., … Menon, U. (2020). **Secondary hypothermia in patients with super-refractory status epilepticus managed with propofol and ketamine**. *Epilepsy and Behavior*, *105*, 106960. https://doi.org/http://dx.doi.org/10.1016/j.yebeh.2020.106960
Background: Therapeutic hypothermia as a potent nonpharmacologic antiseizure therapy has been investigated experimentally in animal models and humans. Although induced hypothermia has been shown to be neuroprotective in acute convulsive status epilepticus, whether its use will translate into improved outcomes for patients with super-refractory nonconvulsive status epilepticus (SRNCSE) has been debated. No clinical data are available on the occurrence and prognostic impact of secondary hypothermia (s-HT) in patients with SRNCSE. With the possibility of core to periphery redistribution of heat with propofol and a centrally mediated dose-dependent fall in body temperature with ketamine, we aimed to investigate the incidence of s-HT events in patients with SRNCSE managed with propofol and ketamine and their impact on clinical outcomes. Method(s): We performed a retrospective observational analysis of consecutive patients with SRNCSE managed with propofol and/or ketamine in a single-center neurological intensive care unit between December 1, 2012 and December 31, 2015. Patients were divided according to the occurrence of hypothermia (temperature < 35.0 degreeC) into an s-HT group and a nonhypothermia (n-HT) group. Patients who received targeted temperature management therapy were excluded. We compared the demographics, comorbidities, treatment characteristics, and outcomes between groups. Result(s): Ninety-nine consecutive patients with SRNCSE managed with propofol and/or ketamine were identified during the study period. Twenty patients who received targeted temperature management were excluded, leaving a total of 79 patients for analysis. Hypothermia was observed in 52% (41/79) of the study population. Ketamine was used in 63/79 patients (80%). Ketamine infusion rates were higher and of longer duration among patients who developed s-HT compared with those who did not (mean dosage: 57.35 +/- 26.6 mcg/kg/min vs 37.17 +/- 15 mcg/kg/min, P = 0.001; duration: 116.36 +/- 81.9 h vs 88 +/- 89.7 h, P = 0.048). Propofol was used in 78/79 patients (99%), with no significant differences in characteristics between groups (mean dosage: 46.44 +/- 20.2 mcg/kg/min vs 36.9 +/- 12.9 mcg/kg/min, P = 0.058; duration: 125.43 +/- 96.4 h vs 102.3 +/- 87.1 h, P = 0.215). No significant differences in demographics, comorbidities, status epilepticus duration and resolution rates, and outcomes were observed between groups. Conclusion(s): In this single-center retrospective analysis of…

57. Juul, S. E., Comstock, B. A., Vu, P. T., Heagerty, P. J., Wadhawan, R., Weiss, M., … Lowe, J. (2020). **A randomized trial of erythropoietin for neuroprotection in preterm infants**. *New England Journal of Medicine*, *382*(3), 233–243. https://doi.org/http://dx.doi.org/10.1056/NEJMoa1907423
BACKGROUND High-dose erythropoietin has been shown to have a neuroprotective effect in preclinical models of neonatal brain injury, and phase 2 trials have suggested possible efficacy; however, the benefits and safety of this therapy in extremely preterm infants have not been established. METHODS In this multicenter, randomized, double-blind trial of high-dose erythropoietin, we assigned 941 infants who were born at 24 weeks 0 days to 27 weeks 6 days of gestation to receive erythropoietin or placebo within 24 hours after birth. Erythropoietin was administered intravenously at a dose of 1000 U per kilogram of body weight every 48 hours for a total of six doses, followed by a maintenance dose of 400 U per kilogram three times per week by subcutaneous injection through 32 completed weeks of postmenstrual age. Placebo was administered as intravenous saline followed by sham injections. The primary outcome was death or severe neurodevelopmental impairment at 22 to 26 months of postmenstrual age. Severe neurodevelopmental impairment was defined as severe cerebral palsy or a composite motor or composite cognitive score of less than 70

(which corresponds to 2 SD below the mean, with higher scores indicating better performance) on the Bayley Scales of Infant and Toddler Development, third edition. RESULTS A total of 741 infants were included in the per-protocol efficacy analysis: 376 received erythropoietin and 365 received placebo. There was no significant difference between the erythropoietin group and the placebo group in the incidence of death or severe neurodevelopmental impairment at 2 years of age (97 children [26%] vs. 94 children [26%]; relative risk, 1.03; 95% confidence interval, 0.81 to 1.32; P = 0.80). There were no significant differences between the groups in the rates of retinopathy of prematurity, intracranial hemorrhage, sepsis, necrotizing enterocolitis, bronchopulmonary dysplasia, or death or in the frequency of serious adverse events. CONCLUSIONS High-dose erythropoietin treatment administered to extremely preterm infants from 24 hours after birth through 32 weeks of postmenstrual age did not result in a lower risk of severe neurodevelopmental impairment or death at 2 years of age.Copyright © 2020 Massachusetts Medical Society.

58. Taccone, F. S., Vincent, J.-L., & Picetti, E. (2020). **High Quality Targeted Temperature Management (TTM) after Cardiac Arrest**. *Critical Care*, *24*(1), 6. https://doi.org/http://dx.doi.org/10.1186/s13054-019-2721-1
Targeted temperature management (TTM) is a complex intervention used with the aim of minimizing post-anoxic injury and improving neurological outcome after cardiac arrest. There is large variability in the devices used to achieve cooling and in protocols (e.g., for induction, target temperature, maintenance, rewarming, sedation, management of post-TTM fever). This variability can explain the limited benefits of TTM that have sometimes been reported. We therefore propose the concept of "high-quality TTM" as a way to increase the effectiveness of TTM and standardize its use in future interventional studies.Copyright © 2020 The Author(s).

59. Mora, A. G., Maddry, J. K., Bebarta, V. S., Reeves, L. K., Bebarta, E. K., Schauer, S. G., & Lairet, J. R. (2020). **Prehospital Use of Ketamine in the Combat Setting: A Sub-Analysis of Patients With Head Injuries Evaluated in the Prospective Life Saving Intervention Study**. *Military Medicine*, *185*(Supplement 1), 136–142. https://doi.org/http://dx.doi.org/10.1093/milmed/usz302
OBJECTIVES: Ketamine is used as an analgesic for combat injuries. Ketamine may worsen brain injury, but new studies suggest neuroprotection. Our objective was to report the outcomes of combat casualties with traumatic brain injury (TBI) who received prehospital ketamine. METHOD(S): This was a post hoc, sub-analysis of a larger prospective, multicenter study (the Life Saving Intervention study [LSI]) evaluating prehospital interventions performed in Afghanistan. A DoD Trauma Registry query provided disposition at discharge and outcomes to be linked with the LSI data. RESULT(S): For this study, we enrolled casualties that were suspected to have TBI (n = 160). Most were 26-year-old males (98%) with explosion-related injuries (66%), a median injury severity score of 12, and 5% mortality. Fifty-seven percent (n = 91) received an analgesic, 29% (n = 46) ketamine, 28% (n = 45) other analgesic (OA), and 43% (n = 69) no analgesic (NA). The ketamine group had more pelvic injuries (P = 0.0302) and tourniquets (P = 0.0041) compared to OA. In comparison to NA, the ketamine group was more severely injured and more likely to require LSI procedures, yet, had similar vital signs at admission and disposition at discharge. CONCLUSION(S): We found that combat casualties with suspected TBI that received prehospital ketamine had similar outcomes to those that received OAs or NAs despite injury differences.Copyright © Association of Military Surgeons of the United States 2020. All rights reserved. For permissions, please e-mail: journals.permissions@oup.com.

60. Suzuki, M., Hatakeyama, T., Nakamura, R., Saiki, T., Kamisasanuki, T., Sugiki, D., & Matsushima, H. (2020). **Serum Magnesium Levels and Neurological Outcomes in Patients Undergoing Targeted Temperature Management After Cardiac Arrest**. *Journal of Emergency Nursing*, *46*(1), 59–65. https://doi.org/http://dx.doi.org/10.1016/j.jen.2019.10.006
INTRODUCTION: Magnesium plays a neuroprotective role at the physiologic level, but its neuroprotective role in patients undergoing targeted temperature management for cardiac arrest is not well established. We performed multiple logistic regression analysis to evaluate whether magnesium levels can predict neurological outcomes in patients undergoing targeted temperature management after cardiac arrest. METHOD(S): We retrospectively investigated data on 86 patients who had undergone targeted temperature management after cardiac arrest between December 2015 and November 2017. The primary outcome was to determine whether magnesium levels predict unfavorable neurological outcomes for patients with return of spontaneous circulation after targeted temperature management. Cerebral Performance Category 3, 4, or 5 indicated unfavorable neurological outcomes. We performed multiple logistic regression to evaluate the primary outcome, adjusting for the time to return of spontaneous circulation, motor score of the Glasgow Coma Scale, first-recorded cardiac rhythm, pH, and magnesium levels. RESULT(S): Of the 86 patients, 58 had unfavorable neurological outcomes. The mean hospital stay was 19 days. Multivariable analysis indicated that magnesium levels were not associated with an unfavorable neurological outcome. In contrast, a time to return of spontaneous circulation greater than 30 minutes and Glasgow Coma Scale motor score of 1 were significantly associated with an unfavorable neurological outcome. DISCUSSION: Magnesium levels were not associated with an unfavorable neurological outcome according to multivariable analysis. We found that a time to return of spontaneous circulation greater than 30 minutes and Glasgow Coma Scale motor score of 1 might predict an unfavorable neurological outcome.Copyright © 2019 Emergency Nurses Association. Published by Elsevier Inc. All rights reserved.

61. Salamah, A., Mehrez, M., El Amrousy, D., Faheem Doaa; ORCID: http://orcid.org/0000-0001-7836-1170, A. A. O.-E. A., & Salamah A Faheem A, El Amrousy D, M. M. (2020). **Efficacy of Citicoline as a Neuroprotector in children**

with post cardiac arrest: a randomized controlled clinical trial. *European Journal of Pediatrics*, *180*(4), 1249–1255. https://doi.org/http://dx.doi.org/10.1007/s00431-020-03871-6
Brain hypoxia after cardiac arrest leads to damage of the neuronal cell membrane. Citicoline is necessary for the synthesis of cell membrane. We planned to assess the neuroprotective effect of citicoline in children after cardiac arrest. This randomized controlled trial was carried out at pediatric intensive care units (PICU) and surgical ICU at Tanta university hospital on 80 consecutive children surviving in-hospital cardiac arrest who were subdivided into two groups. Group I (citicoline group) included 40 children with post-cardiac arrest who received citicoline 10 mg /kg /12 h IV for 6 weeks plus other supportive measures and group II (control group) included 40 children with post-cardiac arrest who were managed with only supportive measures. All patients were evaluated for Glasgow coma score (GCS), modified Rankin scale (mRS) for children, seizures frequency, type and duration, and serum neuron-specific enolase (NSE) before and 3 months after the treatment. GCS and mRS significantly improved in citicholine group compared to the control group. Seizure frequency and duration, mortality, PICU and hospital stay significantly decreased in citicholine group compared to the control group. Serum NSE levels significantly decreased in citicholine group only. No side effects were recorded. Conclusion(s): Citicoline is a promising neuroprotective drug in children with post-cardiac arrest. Trial Registration: The study was registered at Pan African Clinical Trials Registry (PACTR) www.pactr.samrc.ac.za with trial number PACTR201907742119058.What is known?* Post-resuscitation brain injury is one of the major complications that can lead to death or disability.* CDP-choline has been studied for acute ischemic stroke in several adult studies because of its reparative effect.What is new?* Our study was the first in pediatrics that assessed the neuroprotective effect of CDP-choline on the brain in children after cardiac arrest.* We found that Citicoline is a promising neuroprotective drug in children with post-cardiac arrest.Copyright © 2020, Springer-Verlag GmbH Germany, part of Springer Nature.

62. Lee, R. H.-C., Grames, M. S., Wu, C. Y.-C., Lien, C.-F., Couto E Silva, A., Possoit, H. E., … Lin, H. W. (2020). **Upregulation of serum and glucocorticoid-regulated kinase 1 exacerbates brain injury and neurological deficits after cardiac arrest.** *American Journal of Physiology. Heart and Circulatory Physiology*, *319*(5), H1044–H1050. https://doi.org/https://dx.doi.org/10.1152/ajpheart.00399.2020
Cardiopulmonary arrest (CA) is the leading cause of death and disability in the United States. CA-induced brain injury is influenced by multifactorial processes, including reduced cerebral blood flow (hypoperfusion) and neuroinflammation, which can lead to neuronal cell death and functional deficits. We have identified serum and glucocorticoid-regulated kinase-1 (SGK1) as a new target in brain ischemia previously described in the heart, liver, and kidneys (i.e., diabetes and hypertension). Our data suggest brain SGK1 mRNA and protein expression (i.e., hippocampus), presented with hypoperfusion (low cerebral blood flow) and neuroinflammation, leading to further studies of the potential role of SGK1 in CA-induced brain injury. We used a 6-min asphyxia cardiac arrest (ACA) rat model to induce global cerebral ischemia. Modulation of SGK1 was implemented via GSK650394, a SGK1-specific inhibitor (1.2 mug/kg icv). Accordingly, treatment with GSK650394 attenuated cortical hypoperfusion and neuroinflammation (via Iba1 expression) after ACA, whereas neuronal survival was enhanced in the CA1 region of the hippocampus. Learning/memory deficits were observed 3 days after ACA but ameliorated with GSK650394. In conclusion, SGK1 is a major contributor to ACA-induced brain injury and neurological deficits, while inhibition of SGK1 with GSK650394 provided neuroprotection against CA-induced hypoperfusion, neuroinflammation, neuronal cell death, and learning/memory deficits. Our studies could lead to a novel, therapeutic target for alleviating brain injury following cerebral ischemia. NEW & NOTEWORTHY Upregulation of SGK1 exacerbates brain injury during cerebral ischemia. Inhibition of SGK1 affords neuroprotection against cardiac arrest-induced hypoperfusion, neuroinflammation, neuronal cell death, and neurological deficits.

63. Chen, Y., Yang, Z., Wei, L., Wang, J., Xuan, W., Wang, Y., … Li, Y. (2020). **Yes-associated protein protects and rescues SH-SY5Y cells from ketamine-induced apoptosis.** *Molecular Medicine Reports*, *22*(3), 2342–2350. https://doi.org/https://dx.doi.org/10.3892/mmr.2020.11328
Ketamine is a widely used intravenous anesthetic; however, basic and clinical studies have demonstrated that prolonged exposure can cause irreversible injury to the immature human brain. Yes-associated protein (YAP) is the main effector of the Hippo signaling pathway, which serves an important role in regulating tissue homeostasis and organ size during development. However, whether YAP mediates ketamine-induced apoptosis is not completely understood. Based on the functions of YAP during apoptosis resistance and cell self-renewal regulation, the present study hypothesized that YAP serves a role during ketamine-induced apoptosis. An in vitro model was utilized to investigate the effects of ketamine on neurotoxicity and to further investigate the role of YAP in ketamine-induced apoptosis using techniques including CCK-8 assay, flow cytometry and western blotting. The present study assessed the effects of YAP overexpression and knockdown on the expression of typical apoptotic markers in SH-SY5Y cells. Ketamine induced apoptosis in a dose-dependent manner, which was regulated by YAP. Following YAP overexpression, ketamine-treated SH-SY5Y cells displayed increased activity and viability, whereas expression levels of the apoptotic markers were decreased compared with the negative control group. By contrast, ketamine-induced apoptosis was enhanced following YAP knockdown. Collectively, the results of the present study indicated that YAP may serve an important role during ketamine-induced neurotoxicity, and alterations to YAP signaling may counteract ketamine-induced apoptosis. The neuroprotective effect of YAP activation may serve as a novel pharmacological target for the treatment of ketamine-induced neurotoxicity via neurogenesis normalization.

64. Gursoy, I. D., Barun, S., Erdem, S. R., Keskin, U., Kiziltas, M., Atilla, P., … Canpinar, H. (2020). **Investigation of**

the Possible Protective Effects of Ketamine and Dantrolene on the Hippocampal Apoptosis and Spatial Learning in Rats Exposed to Repeated Electroconvulsive Seizures as a Model of Status Epilepticus. *Turkish Neurosurgery*, *30*(6), 871–884. https://doi.org/https://dx.doi.org/10.5137/1019-5149.JTN.27023-19.3
AIM: To evaluate the possible neuroprotective effects of ketamine and dantrolene on the hippocampal apoptosis and spatial learning in rats exposed to repeated electroconvulsive seizures (ECS) as a model of status epilepticus (SE)., MATERIAL AND METHODS: Twenty-four rats were assigned to 4 groups. 1st Group was Sham. 2nd Group was ECS: ECS was induced by ear electrodes via electrical stimulation. The same ECS protocol was applied to the 3th and 4th Groups which received ketamine (40 mg/kg s.c.) or dantrolene (5 mg/kg i.p.) 1 h before each ECS, respectively. Following 30 days of recovery, the cognitive status of the animals was evaluated via Morris Water Maze (MWM). The same experimental protocol was repeated 14 days afterward to evaluate the retention of the memory. Hippocampal apoptosis was examined in corresponding experimental groups., RESULTS: All the animals in four groups learned the task with no significant difference between groups in MWM. The ECS+ketamine group showed memory impairment 14 days afterward. ECS+dantrolene group was not different from controls. ECS caused long term apoptotic processes in dentate gyrus (DG) and non-apoptotic neuronal injury in CA1 and CA2., CONCLUSION: Dantrolene and ketamine inhibited apoptosis and showed neuroprotective effects. Although ketamine and dantrolene inhibited ECS-induced apoptosis and non-apoptotic injury, they did not produce similar effects on memory retention. It will be warranted to evaluate cognitive dysfunction by taking into consideration the other factors in addition to apoptosis and neurodegenerative changes.

65. Boissady, E., Kohlhauer, M., Lidouren, F., Hocini, H., Lefebvre, C., Chateau-Jouber, S., … Tissier, R. (2020). **Ultrafast Hypothermia Selectively Mitigates the Early Humoral Response After Cardiac Arrest.** *Journal of the American Heart Association*, *9*(23), e017413. https://doi.org/https://dx.doi.org/10.1161/JAHA.120.017413
Background Total liquid ventilation (TLV) has been shown to prevent neurological damage though ultrafast cooling in animal models of cardiac arrest. We investigated whether its neuroprotective effect could be explained by mitigation of early inflammatory events. Methods and Results Rabbits were submitted to 10 minutes of ventricular fibrillation. After resuscitation, they underwent normothermic follow-up (control) or ultrafast cooling by TLV and hypothermia maintenance for 3 hours (TLV). Immune response, survival, and neurological dysfunction were assessed for 3 days. TLV improved neurological recovery and reduced cerebral lesions and leukocyte infiltration as compared with control (eg, neurological dysfunction score=34+/-6 versus 66+/-6% at day 1, respectively). TLV also significantly reduced interleukin-6 blood levels during the hypothermic episode (298+/-303 versus 991+/-471 pg/mL in TLV versus control at 3 hours after resuscitation, respectively), but not after rewarming (752+/-563 versus 741+/-219 pg/mL in TLV versus control at 6 hours after resuscitation, respectively). In vitro assays confirmed the high temperature sensitivity of interleukin-6 secretion. Conversely, TLV did not modify circulating high-mobility group box 1 levels or immune cell recruitment into the peripheral circulation. The link between interleukin-6 early transcripts (<8 hours) and neurological outcome in a subpopulation of the previously described Epo-ACR-02 (High Dose of Erythropoietin Analogue After Cardiac Arrest) trial confirmed the importance of this cytokine at the early stages as compared with delayed stages (>8 hours). Conclusions The neuroprotective effect of hypothermic TLV was associated with a mitigation of humoral interleukin-6 response. A temperature-dependent attenuation of immune cell reactivity during the early phase of the post-cardiac arrest syndrome could explain the potent effect of rapid hypothermia. Registration URL: https://www.clinicaltrials.gov; Unique identifier: NCT00999583.

66. Fumagalli, F., Olivari, D., Boccardo, A., De Giorgio, D., Affatato, R., Ceriani, S., … Ristagno, G. (2020). **Ventilation With Argon Improves Survival With Good Neurological Recovery After Prolonged Untreated Cardiac Arrest in Pigs.** *Journal of the American Heart Association*, *9*(24), e016494. https://doi.org/https://dx.doi.org/10.1161/JAHA.120.016494
Background Ventilation with the noble gas argon (Ar) has shown neuroprotective and cardioprotective properties in different in vitro and in vivo models. Hence, the neuroprotective effects of Ar were investigated in a severe, preclinically relevant porcine model of cardiac arrest. Methods and Results Cardiac arrest was ischemically induced in 36 pigs and left untreated for 12 minutes before starting cardiopulmonary resuscitation. Animals were randomized to 4-hour post-resuscitation ventilation with: 70% nitrogen-30% oxygen (control); 50% Ar-20% nitrogen-30% oxygen (Ar 50%); and 70% Ar-30% oxygen (Ar 70%). Hemodynamic parameters and myocardial function were monitored and serial blood samples taken. Pigs were observed up to 96 hours for survival and neurological recovery. Heart and brain were harvested for histopathology. Ten animals in each group were successfully resuscitated. Ninety-six-hour survival was 60%, 70%, and 90%, for the control, Ar 50%, and Ar 70% groups, respectively. In the Ar 50% and Ar 70% groups, 60% and 80%, respectively, achieved good neurological recovery, in contrast to only 30% in the control group (P<0.0001). Histology showed less neuronal degeneration in the cortex (P<0.05) but not in the hippocampus, and less reactive microglia activation in the hippocampus (P=0.007), after Ar compared with control treatment. A lower increase in circulating biomarkers of brain injury, together with less kynurenine pathway activation (P<0.05), were present in Ar-treated animals compared with controls. Ar 70% pigs also had complete left ventricular function recovery and smaller infarct and cardiac troponin release (P<0.01). Conclusions Post-resuscitation ventilation with Ar significantly improves neurologic recovery and ameliorates brain injury after cardiac arrest with long no-flow duration. Benefits are greater after Ar 70% than Ar 50%.

67. Li, J., Wang, J., Shen, Y., Dai, C., Chen, B., Huang, Y., … Li, Y. (2020). **Hyperoxygenation With**

Cardiopulmonary Resuscitation and Targeted Temperature Management Improves Post-Cardiac Arrest Outcomes in Rats. *Journal of the American Heart Association*, *9*(19), e016730. https://doi.org/https://dx.doi.org/10.1161/JAHA.120.016730
Background Oxygen plays a pivotal role in cardiopulmonary resuscitation (CPR) and postresuscitation intervention for cardiac arrest. However, the optimal method to reoxygenate patients has not been determined. This study investigated the effect of timing of hyperoxygenation on neurological outcomes in cardiac arrest/CPR rats treated with targeted temperature management. Methods and Results After induction of ventricular fibrillation, male Sprague-Dawley rats were randomized into 4 groups (n=16/group): (1) normoxic control; (2) O2_CPR, ventilated with 100% O2 during CPR; (3) O2_CPR+postresuscitation, ventilated with 100% O2 during CPR and the first 3 hours of postresuscitation; and (4) O2_postresuscitation, ventilated with 100% O2 during the first 3 hours of postresuscitation. Targeted temperature management was induced immediately after resuscitation and maintained for 3 hours in all animals. Postresuscitation hemodynamics, neurological recovery, and pathological analysis were assessed. Brain tissues of additional rats undergoing the same experimental procedure were harvested for ELISA-based quantification assays of oxidative stress-related biomarkers and compared with the sham-operated rats (n=6/group). We found that postresuscitation mean arterial pressure and quantitative electroencephalogram activity were significantly increased, whereas astroglial protein S100B, degenerated neurons, oxidative stress-related biomarkers, and neurologic deficit scores were significantly reduced in the O2_CPR+postresuscitation group compared with the normoxic control group. In addition, 96-hour survival rates were significantly improved in all of the hyperoxygenation groups. Conclusions In this cardiac arrest/CPR rat model, hyperoxygenation coupled with targeted temperature management attenuates ischemia/reperfusion-induced injuries and improves survival rates. The beneficial effects of high-concentration oxygen are timing and duration dependent. Hyperoxygenation commenced with CPR, which improves outcomes when administered during hypothermia.

68. Wang, C.-H., Chang, W.-T., Huang, C.-H., Tsai, M.-S., Liu, S.-H., & Chen, W.-J. (2020). **Cerebral Blood Flow-Guided Manipulation of Arterial Blood Pressure Attenuates Hippocampal Apoptosis After Asphyxia-Induced Cardiac Arrest in Rats.** *Journal of the American Heart Association*, *9*(13), e016513. https://doi.org/https://dx.doi.org/10.1161/JAHA.120.016513
Background In most post-cardiac arrest patients, the autoregulation mechanism of cerebral blood flow (CBF) is dysregulated. We examined whether recovery of CBF by adjusting mean arterial pressure mitigates post-cardiac arrest neuronal damage. Methods and Results Wistar rats that underwent 8-minute asphyxia-induced cardiac arrest and resuscitation were computer-randomized to norepinephrine or control groups. The CBF was measured at the dorsal hippocampal CA1 region of the left hemisphere. In the norepinephrine group, the mean arterial pressure was adjusted to recover CBF to 80% to 100% of baseline. Twenty-four hours following resuscitation, neurological outcomes were assessed, and brain tissues and blood samples were harvested for neuronal apoptosis and injury assessment. Thirty resuscitated rats were randomized into 2 groups, each containing 12 rats that completed the experiments. Norepinephrine infusion effectively prevented posthyperemia hypoperfusion and recovered CBF to pre-arrest baseline levels; a moderate positive linear correlation between mean arterial pressure and CBF during this period was also observed (P<0.001). There were no significant between-group differences in neurological recovery. In the norepinephrine group compared with the control group, upregulated cleaved caspase-3 protein expression in brain tissue determined by Western blot was reduced (P=0.02) and the densities of apoptotic cells in hippocampal CA1 and CA3 regions determined by terminal deoxynucleotidyl transferase-mediated dUTP biotin nick-end labeling were decreased (P<0.001). No significant differences in serum neuron-specific enolase or S100beta levels were detected between the 2 groups. Conclusions CBF recovery demonstrated neuroprotective effects by reducing activation of cerebral apoptosis and number of apoptotic neurons. However, these effects did not significantly improve clinical neurological function, necessitating further investigation.

69. Zhou, X., Lv, X., Zhang, L., Yan, J., Hu, R., Sun, Y., … Jiang, H. (2020). **Ketamine promotes the neural differentiation of mouse embryonic stem cells by activating mTOR.** *Molecular Medicine Reports*, *21*(6), 2443–2451. https://doi.org/https://dx.doi.org/10.3892/mmr.2020.11043
Ketamine is a widely used general anesthetic and has been reported to demonstrate neurotoxicity and neuroprotection. Investigation into the regulatory mechanism of ketamine on influencing neural development is of importance for a better and safer way of relieving pain. Reverse transcription-quantitative polymerase chain reaction and western blotting were used to detect the critical neural associated gene expression, and flow cytometry to detect the neural differentiation effect. Hence, in the present study the underlying mechanism of ketamine (50 nM) on neural differentiation of the mouse embryonic stem cell (mESC) line 46C was investigated. The results demonstrated that a low dose of ketamine (50 nM) promoted the differentiation of mESCs to neural stem cells (NSCs) and activated mammalian target of rapamycin (mTOR) by upregulating the expression levels of phosphorylated (p)-mTOR. Furthermore, inhibition of the mTOR signaling pathway by rapamycin or knockdown of mTOR suppressed neural differentiation. A rescue experiment further confirmed that downregulation of mTOR inhibited the promotion of neural differentiation induced by ketamine. Taken together, the present study indicated that a low level of ketamine upregulated p-mTOR expression levels, promoting neural differentiation.

70. Hu, Y., Sun, D., Li, Y., Wang, X., Jiang, W., Shi, H., & Cui, D. (2020). **Increased PINK1/Parkin-mediated mitophagy explains the improved brain protective effects of slow rewarming following hypothermia after**

cardiac arrest in rats. *Experimental Neurology*, *330*, 113326. https://doi.org/https://dx.doi.org/10.1016/j.expneurol.2020.113326
Cerebral ischemia-reperfusion (I/R) after cardiac arrest (CA) induces mitochondrial dysfunction, and the timely removal of damaged mitochondria by mitophagy is reported to protect against cerebral I/R injury. Therapeutic hypothermia (TH) has become an important component of postresuscitation care for patients who return to spontaneous circulation after CA. Previous studies have shown that TH can activate mitophagy and can contribute a protective effect; however, the optimal rewarming rate and underlying mechanism of rewarming following TH remain largely unexplained. Here, we investigated the effects of different rewarming rates and whether mitophagy is involved in rewarming. After 5 min of asphyxial CA following 4 h of cooling, Sprague-Dawley rats were randomized into the normothermia, hypothermia, slow rewarming (0.5 degreeC/h) and fast rewarming (4 degreeC/h) groups. The hypothermia group was kept cool until tissue harvest, the rewarming duration for the slow rewarming group and fast rewarming group was 6 h and 45 min, respectively. We found that slowly rewarmed rats had better survival at 72 h than normothermic rats and fast-rewarmed rats (70%, 25.71%, and 50%, respectively) and higher neurological deficit scores (NDSs), in which the medians were 57.33, 26, and 28.83, respectively. In addition, we explored the underlying mechanism during this process and found that PINK1/Parkin-mediated mitophagy was activated during hypothermia in the slow rewarming group but was inhibited in the fast rewarming group. Further inhibition of mitophagy in the slowly rewarmed rats resulted in severe apoptosis and decreased the mean NDS from 58.39 to 33.11, indicating the protective role of mitophagy. Moreover, the fast rewarming group exhibited deficiencies in PINK1 expression and mitophagy activity and marked accumulation of reactive oxygen species (ROS). Overall, our results highlighted a neuroprotective role of PINK1/Parkin-mediated mitophagy during slow rewarming after hypothermia. Copyright © 2020. Published by Elsevier Inc.

71. Alshami, A., Einav, S., Skrifvars, M. B., & Varon, J. (2020). **Administration of inhaled noble and other gases after cardiopulmonary resuscitation: A systematic review.** *The American Journal of Emergency Medicine*, *38*(10), 2179–2184. https://doi.org/https://dx.doi.org/10.1016/j.ajem.2020.06.066
OBJECTIVE: Inhalation of noble and other gases after cardiac arrest (CA) might improve neurological and cardiac outcomes. This article discusses up-to-date information on this novel therapeutic intervention., DATA SOURCES: CENTRAL, MEDLINE, online published abstracts from conference proceedings, clinical trial registry clinicaltrials.gov, and reference lists of relevant papers were systematically searched from January 1960 till March 2019., STUDY SELECTION: Preclinical and clinical studies, irrespective of their types or described outcomes, were included., DATA EXTRACTION: Abstract screening, study selection, and data extraction were performed by two independent authors. Due to the paucity of human trials, risk of bias assessment was not performed DATA SYNTHESIS: After screening 281 interventional studies, we included an overall of 27. Only, xenon, helium, hydrogen, and nitric oxide have been or are being studied on humans. Xenon, nitric oxide, and hydrogen show both neuroprotective and cardiotonic features, while argon and hydrogen sulfide seem neuroprotective, but not cardiotonic. Most gases have elicited neurohistological protection in preclinical studies; however, only hydrogen and hydrogen sulfide appeared to preserve CA1 sector of hippocampus, the most vulnerable area in the brain for hypoxia., CONCLUSION: Inhalation of certain gases after CPR appears promising in mitigating neurological and cardiac damage and may become the next successful neuroprotective and cardiotonic interventions. Copyright © 2020 Elsevier Inc. All rights reserved.

72. Bialon, M., Zarnowska, M., Antkiewicz-Michaluk, L., & Wasik, A. (2020). **Pro-cognitive effect of 1MeTIQ on recognition memory in the ketamine model of schizophrenia in rats: the behavioural and neurochemical effects.** *Psychopharmacology*, *237*(6), 1577–1593. https://doi.org/https://dx.doi.org/10.1007/s00213-020-05484-1
RATIONALE: Schizophrenia is a mental illness which is characterised by positive and negative symptoms and by cognitive impairments. While the major prevailing hypothesis is that altered dopaminergic and/or glutamatergic transmission contributes to this disease, there is evidence that the noradrenergic system also plays a role in its major symptoms., OBJECTIVES: In the present paper, we investigated the pro-cognitive effect of 1-methyl-1,2,3,4-tetrahydroisoquinoline (1MeTIQ) an endogenous neuroprotective compound, on ketamine-modelled schizophrenia in rats., METHODS: We used an antagonist of NMDA receptors (ketamine) to model memory deficit symptoms in rats. Using the novel object recognition (NOR) test, we investigated the pro-cognitive effect of 1MeTIQ. Additionally, olanzapine, an atypical antipsychotic drug, was used as a standard to compare the pro-cognitive effects of the substances. In vivo microdialysis studies allowed us to verify the changes in the release of monoamines and their metabolites in the rat striatum., RESULTS: Our study demonstrated that 1MeTIQ, similarly to olanzapine, exhibits a pro-cognitive effect in NOR test and enhances memory disturbed by ketamine treatment. Additionally, in vivo microdialysis studies have shown that ketamine powerfully increased noradrenaline release in the rat striatum, while 1MeTIQ and olanzapine completely antagonised this neurochemical effect., CONCLUSIONS: 1MeTIQ, as a possible pro-cognitive drug, in contrast to olanzapine, expresses beneficial neuroprotective activity in the brain, increasing concentration of the extraneuronal dopamine metabolite, 3-methoxytyramine (3-MT), which plays an important physiological role in the brain as an inhibitory regulator of catecholaminergic activity. Moreover, we first demonstrated the essential role of noradrenaline release in memory disturbances observed in the ketamine-model of schizophrenia, and its possible participation in negative symptoms of the schizophrenia.

73. Ameloot K Hastbacka J, Reinikainen M, Pettila V, Loisa P, Tiainen M, Bendel S, Birkelund T, Belmans A, Palmers PJ, Bogaerts E, Lemmens R, De Deyne C, Ferdinande B, Dupont M, Janssens S, Dens J, Skrifvars MB, J. P., Ameloot, K.,

Literature Search Results

Jakkula, P., Hastbacka, J., Reinikainen, M., Pettila, V., … Skrifvars, M. B. (2020). **Optimum Blood Pressure in Patients With Shock After Acute Myocardial Infarction and Cardiac Arrest.** *Journal of the American College of Cardiology*, *76*(7), 812. https://doi.org/https://dx.doi.org/10.1016/j.jacc.2020.06.043

Background: In patients with shock after acute myocardial infarction (AMI), the optimal level of pharmacologic support is unknown. Whereas higher doses may increase myocardial oxygen consumption and induce arrhythmias, diastolic hypotension may reduce coronary perfusion and increase infarct size. Objectives: This study aimed to determine the optimal mean arterial pressure (MAP) in patients with AMI and shock after cardiac arrest. Methods: This study used patient-level pooled analysis of post-cardiac arrest patients with shock after AMI randomized in the Neuroprotect (Neuroprotective Goal Directed Hemodynamic Optimization in Post-cardiac Arrest Patients; NCT02541591) and COMACARE (Carbon Dioxide, Oxygen and Mean Arterial Pressure After Cardiac Arrest and Resuscitation; NCT02698917) trials who were randomized to MAP 65 mm Hg or MAP 80/85 to 100 mm Hg targets during the first 36 h after admission. The primary endpoint was the area under the 72-h high-sensitivity troponin-T curve. Results: Of 235 patients originally randomized, 120 patients had AMI with shock. Patients assigned to the higher MAP target (n = 58) received higher doses of norepinephrine (p = 0.004) and dobutamine (p = 0.01) and reached higher MAPs (86 +/- 9 mm Hg vs. 72 +/- 10 mm Hg, p < 0.001). Whereas admission hemodynamics and angiographic findings were all well-balanced and revascularization was performed equally effective, the area under the 72-h high-sensitivity troponin-T curve was lower in patients assigned to the higher MAP target (median: 1.14 [mu]g.72 h/l [interquartile range: 0.35 to 2.31 [mu]g.72 h/l] vs. median: 1.56 [mu]g.72 h/l [interquartile range: 0.61 to 4.72 [mu]g. 72 h/l]; p = 0.04). Additional pharmacologic support did not increase the risk of a new cardiac arrest (p = 0.88) or atrial fibrillation (p = 0.94). Survival with good neurologic outcome at 180 days was not different between both groups (64% vs. 53%, odds ratio: 1.55; 95% confidence interval: 0.74 to 3.22). Conclusions: In post-cardiac arrest patients with shock after AMI, targeting MAP between 80/85 and 100 mm Hg with additional use of inotropes and vasopressors was associated with smaller myocardial injury.
