# Supplementary material for: The use of ketamine as a neuroprotective agent following cardiac arrest: A scoping review of current literature
Source: CNS Neurosci Ther. 2022 Oct 2;29(1):104–10. doi: 10.1111/cns.13983 (PMC9804040; doi:10.1111/cns.13983)
Supplement: Supplementary file 3 — Appendix S3 [file CNS-29-104-s003.pdf]

Date Completed: 2020-05-28

Requestor: Marlena Ornowska

Request:

1) information about cardiac arrest as a model used for neuro-protection studies. Most of the time in these types of studies, a stroke model is used. We are interested in finding out whether anyone has investigated usage of cardiac arrest as a model for neurological injury.

2) Neuronal injury following cardiac arrest and neuro-protection.

3) General literature search about ketamine in clinical practice in neuroprotection. Perhaps this search can include the following terms: ketamine, neuroprotection, experimental (any work)?

Completed By: Erin Brady-Randle

Search Strategy:

Databases Searched:
Medline
CINAHL
EMBASE
Cochrane Database of Systematic Reviews
Cochrane Central Register of Controlled Trials

Terms searched:
MeSH:
Ketamine
Neuroprotective Agents
Neuroprotection
Heart Arrest

CINAHL:
Ketamine
Neuroprotective Agents
Heart Arrest

Embase SH:
ketamine
neuroprotection
heart arrest

Keywords:
neuron* n3 injur*
neuroprotect*

Results:

Cardiac Arrest Models for Neuroprotection Studies (A)

1. A1. J, Z., Y, W., M, J., J, S., Y, Z., S, L., D, Z., L, W., M, Z., S, P., & G, Y. (2020). Neuroprotective Effect of the Inhibitor Salubrinal After Cardiac Arrest in a Rodent Model. Oxidative Medicine and Cellular Longevity, 2020. https://doi.org/10.1155/2020/7468738

Cardiac arrest (CA) yields poor neurological outcomes. Salubrinal (Sal), an endoplasmic reticulum (ER) stress inhibitor, has been shown to have neuroprotective effects in both in vivo and in vitro brain injury models. This study investigated the neuroprotective mechanisms of Sal in postresuscitation brain damage in a rodent model of CA. In the present study, rats were subjected to 6 min of CA and then successfully resuscitated. Either Sal (1 mg/kg) or vehicle (DMSO) was injected blindly 30 min before the induction of CA. Neurological status was assessed 24 h after CA, and the cortex was collected for analysis. As a result, we observed that, compared with the vehicle-treated animals, the rats pretreated with Sal exhibited markedly improved neurological performance and cortical mitochondrial morphology 24 h after CA. Moreover, Sal pretreatment was associated with the following: (1) upregulation of superoxide dismutase activity and a reduction in maleic dialdehyde content; (2) preserved mitochondrial membrane potential; (3) amelioration of the abnormal distribution of cytochrome C; and (4) an

increased Bcl-2/Bax ratio, decreased cleaved caspase 3 upregulation, and enhanced HIF-1α expression. Our findings suggested that Sal treatment improved neurological dysfunction 24 h after CPR (cardiopulmonary resuscitation), possibly through mitochondrial preservation and stabilizing the structure of HIF-1α.

1. A2. Yang, X., Wang, Z., & Jia, X. (2019). Neuroprotection of Glibenclamide against Brain Injury after Cardiac Arrest via Modulation of NLRP3 Inflammasome. Proceedings of the Annual International Conference of the IEEE Engineering in Medicine and Biology Society, EMBS, 2019, 4209–4212. https://doi.org/10.1109/EMBC.2019.8857285

Glibenclamide (GBC) improves cerebral outcome after cardiac arrest (CA) in rats. We aim to investigate the effect of GBC on electrophysiological recovery and to explore the mechanism of neuroprotective effects of GBC on the acute stage of brain injury after the return of spontaneous circulation (ROSC) in a rodent model of CA. 16 anesthetized male Wistar rats subjected to 8-min asphyxia-CA were randomly assigned to the GBC or control group (N=8 each group). GBC was administered with a loading dose of 10ug/kg i. p. injection 10 min after ROSC and followed with a maintaining dose of 1.6ug/kg per 8 hours throughout the first 24 hours. Quantitative measures of EEG-information quantity (qEEG-IQ) and neurological deficit score (NDS) were used to predict and evaluate the functional outcome. There was a significant improvement of NDS in rats treated with GBC compared with the control group (p <; 0.01). Compared to the control group, the rats treated with GBC showed qEEG-IQ scores that indicated better recovery (p <; 0.001). Meanwhile, early QEEG-IQ was significantly correlated with 72-hr NDS as early as 45min after ROSC. Furthermore, on the molecular basis, the NLRP3 inflammasome was strongly activated in the hippocampal CA1 area 3 days after CA in control rats, which was suppressed with GBC treatment. Taken together, GBC treatment markedly improved electrophysiological and neurologic outcomes of the acute brain injury after CA. These neuroprotective effects may be associated with the attenuation of inflammatory response via down-regulation of NLRP3 inflammasome signal.

1. A3. Qin, J., Wang, P., Li, Y., Yao, L., Liu, Y., Yu, T., Lin, J., Fang, X., & Huang, Z. (2019). Activation of Sigma-1 Receptor by Cutamesine Attenuates Neuronal Apoptosis by Inhibiting Endoplasmic Reticulum Stress and Mitochondrial Dysfunction in a Rat Model of Asphyxia Cardiac Arrest. SHOCK, 51(1), 105–113. https://doi.org/10.1097/SHK.0000000000001119

Background: Global cerebral ischemic/reperfusion (I/R) injury after cardiac arrest (CA) is a major cause of mortality and morbidity in survivors of resuscitation. We utilized a rat model of asphyxia CA to explore the functional effects and mechanisms of Sigma-1 receptor (Sig-1R) activation in cerebral protection using the Sig-1R agonist cutamesine (SA-4503).

Methods: After resuscitation, the surviving rats were randomly divided into three groups (n = 18 each): the cardiopulmonary resuscitation (CPR) group (0.9% saline at 1 mL/kg); the SA4503 low-dose group (1 mg/kg SA4503); and the SA4503 high-dose group (2.5 mg/kg SA4503). The neurological deficit scores were recorded, and the cerebral cortex was harvested for western blotting. Mitochondrial transmembrane potential, adenosine triphosphate (ATP) concentrations, calcium homeostasis, and mitochondrial ultrastructure were also studied.

Results: The SA4503 treatment groups exhibited improved neurological outcomes compared with the CPR group. The protein levels of caspase-3 and the endoplasmic reticulum stress markers C/EBP homologous protein and caspase-12 were lower in the SA4503 treatment groups compared with the CPR group. SA4503 treatment also normalized mitochondrial membrane potential, tissue ATP concentrations, intracellular $Ca2+$ overload, and upregulated Sig-1R protein level compared with the CPR group. The SA4503 high dose treatment showed significant cerebral protective effects compared with the SA4503 low dose treatment. The therapeutic effect of SA4503 was dose-dependent.

Conclusions: CA downregulated Sig-1R protein expression. Activating Sig-1R using SA4503 protected against global cerebral I/R injury in a rat model of asphyxia CA by alleviating endoplasmic reticulum stress and mitochondrial dysfunction and eventually inhibiting neuronal apoptosis.

1. A4. Qin, S., Chen, M. H., Fang, W., Tan, X. F., Xie, L., Yang, Y. G., Qin, T., & Li, N. (2019). Cerebral protection of epigallocatechin gallate (EGCG) via preservation of mitochondrial function and ERK inhibition in a rat resuscitation model. Drug Design, Development and Therapy, 13, 2759–2768. https://doi.org/10.2147/DDDT.S215358

Background: Various and opposite roles of epigallocatechin gallate (EGCG) have been reported in different studies. We aimed to investigate how EGCG affects the cerebral injury in a cardiac arrest/cardiopulmonary resuscitation (CA/CPR) model of rat.

Methods: The rats which were subjected to CA/CPR randomly received low dose of EGCG (3 mg/kg, Low-EGCG group, n=16), high dose of EGCG (9 mg/kg, High-EGCG group, n=16) and equal volume of 0.9% saline solution (NS group, n=16) at the first minute after return of spontaneous circulation (ROSC). The rats underwent anesthesia and intubation were defined as Sham group (n=16). Twenty-four hours after ROSC, neural defect score (NDS), ROS fluorescence intensity, degree of mitochondrial permeability transition pore (mPTP) opening, ATP contents and mitochondrial ATP synthase expression were evaluated in the four groups. The expression of extracellular signal-regulated kinase (ERK) activity and cleaved-caspase 3 were also detected by Western blot.

Results: CA/CPR induced severe ischemia-reperfusion injury (IRI), resulted in mitochondrial dysfunction and upregulated phosphorylation of ERK. EGCG dose-dependently alleviated the IRI after CA/CPR, inhibited ERK activity and restored mitochondrial function and, as indicated by improved NDS, reduced ROS level, decreased mPTP opening, elevated ATP

content, increased ATPase expression and downregulated cleaved-caspase 3 level.

Conclusion: EGCG alleviated global cerebral IRI by restoring mitochondrial dysfunction and ERK modulation in a rat CA/CPR model, which might make it a potential candidate agent against IRI after CA/CPR in the future. Further study is needed to determine whether higher dosage of EGCG might aggravate cerebral IRI post-CA/CPR.

1. A5. Li, F., Wei, H., Li, H., Li, X., Hu, C., Zhang, J., Deng, Y., & Liao, X. (2019). MiR-26a prevents neural stem cells from apoptosis via β-catenin signaling pathway in cardiac arrest-induced brain damage. Bioscience Reports, 39(5). https://doi.org/10.1042/BSR20181635

Neural stem cells (NSCs) transplantation is one of the most promising strategies for the treatment of CA-induced brain damage. The transplanted NSCs could differentiate into new neuron and replace the damaged one. However, the poor survival of NSCs in severe hypoxic condition is the limiting step to make the best use of this kind of therapy. In the present study, we investigated whether the overexpression of miR-26a improves the survival of NSCs in hypoxic environment in vitro and in vivo. In vitro hypoxia injury model is established in NSCs by $CoCl_2$ treatment, and in vivo cardiac arrest (CA) model is established in Sprague-Dawley (SD) rats. Quantitative real-time polymerase chain reaction is used to detect the mRNA level and Western blot is used to examine the protein level of indicated genes. TUNEL staining and flow cytometry are applied to evaluate apoptosis. Dual-luciferase reporter assay is utilized to analyze the target gene of miR-26a. The expression of miR-26a is reduced in both in vitro and in vivo hypoxic model. MiR-26a directly targets 3′-UTR of glycogen synthase kinase 3β (GSK-3β), resulting in increased β-catenin expression and decreased apoptosis of NSCs. Overexpression of miR-26a in transplanted NSCs improves the survival of NSCs and neurological function in CA rats. MiR-26a prevents NSCs from apoptosis by activating β-catenin signaling pathway in CA-induced brain damage model. Modulating miR-26a expression could be a potential strategy to attenuate brain damage induced by CA.

1. A6. Wu, J., Li, Y., Yang, P., Huang, Y., Lu, S., & Xu, F. (2019). Novel Role of Carbon Monoxide in Improving Neurological Outcome After Cardiac Arrest in Aged Rats: Involvement of Inducing Mitochondrial Autophagy. Journal of the American Heart Association, 8(9). https://doi.org/10.1161/JAHA.118.011851

Background: Dysfunctional mitochondria are associated with neurological injury after cardiac arrest (CA). Although carbon monoxide (CO) has shown various potential therapeutic effects in preclinical tissue injury models, its mechanism of action in CA remains unclear. We sought to investigate the effects of a novel CO-releasing molecule on cerebral mitochondrial dysfunction and neurological injury after CA.

Methods and Results: Male Sprague-Dawley rats aged 20 to 22 months were subjected to 6-minute asphyxia CA before receiving CO treatment. Survival, neurologic deficit scores, neuronal death, mitochondrial function, and autophagy were evaluated after the return of spontaneous circulation. Results showed that CO post-treatment increased 3-day survival rate from 25% to 70.83% and reduced neurologic deficit scores. CO also ameliorated CA-induced neuronal apoptosis and necrosis in the cerebral cortex and improved cerebral mitochondrial function by reducing reactive oxygen species, reversing mitochondrial membrane potential depolarization, and preventing cytochrome C release. Furthermore, CO increased mitochondrial autophagy by inducing mitochondrial accumulation of PINK1 (PTEN-induced putative kinase 1) and Parkin. Downregulation of PINK1 with genetic silencing siRNA abolished CO-afforded mitochondrial autophagy.

Conclusions: Taken together, our results indicate, for the first time, that CO treatment confers neuroprotection against ischemic neurological injury after CA possibly by promoting mitochondrial autophagy.

1. A7. Keilhoff, G., Titze, M., Rathert, H., Lucas, B., Esser, T., & Ebmeyer, U. (2020). Normoxic post-ROSC ventilation delays hippocampal CA1 neurodegeneration in a rat cardiac arrest model, but does not prevent it. Experimental Brain Research, 238(4), 807–824. https://doi.org/10.1007/s00221-020-05746-6

The European Resuscitation Guidelines recommend that survivors of cardiac arrest (CA) be resuscitated with 100% O_2 and undergo subsequent—post-return of spontaneous circulation (ROSC)—reduction of O_2 supply to prevent hyperoxia. Hyperoxia produces a "second neurotoxic hit," which, together with the initial ischemic insult, causes ischemia–reperfusion injury. However, heterogeneous results from animal studies suggest that normoxia can also be detrimental. One clear reason for these inconsistent results is the considerable heterogeneity of the models used. In this study, the histological outcome of the hippocampal CA1 region following resuscitation with 100% O_2 combined with different post-ROSC ventilation regimes (21%, 50%, and 100% O_2) was investigated in a rat CA/resuscitation model with survival times of 7 and 21 days. Immunohistochemical stainings of NeuN, MAP2, GFAP, and IBA1 revealed a neuroprotective potency of post-ROSC ventilation with 21% O_2, although it was only temporary. This limitation should be because of the post-ROSC intervention targeting only processes of ischemia-induced secondary injury. There were no ventilation-dependent effects on either microglial activation, reduction of which is accepted as being neuroprotective, or astroglial activation, which is accepted as being able to enhance neurons' resistance to ischemia/reperfusion injury. Furthermore, our findings verify the limited comparability of animal studies because of the individual heterogeneity of the animals, experimental regimes, and evaluation procedures used.

1. A8. Taccone, F. S., Picetti, E., & Vincent, J. L. (2020). High Quality Targeted Temperature Management (TTM) after Cardiac Arrest. Critical Care, 24(1). https://doi.org/10.1186/s13054-019-2721-1

Targeted temperature management (TTM) is a complex intervention used with the aim of minimizing post-anoxic injury and improving neurological outcome after cardiac arrest. There is large variability in the devices used to achieve cooling and in protocols (e.g., for induction, target temperature, maintenance, rewarming, sedation, management of post-TTM fever). This variability can explain the limited benefits of TTM that have sometimes been reported. We therefore propose the concept of "high-quality TTM" as a way to increase the effectiveness of TTM and standardize its use in future interventional studies.

1. A9. Chalkias, A., Arnaoutoglou, E., & Xanthos, T. (2019). Personalized physiology-guided resuscitation in highly monitored patients with cardiac arrest—the PERSEUS resuscitation protocol. In Heart Failure Reviews (Vol. 24, Issue 4, pp. 473–480). Springer New York LLC. https://doi.org/10.1007/s10741-019-09772-7

Resuscitation guidelines remain uniform across all cardiac arrest patients, focusing on the delivery of chest compressions to a standardized rate and depth and algorithmic vasopressor dosing. However, individualizing resuscitation to the appropriate hemodynamic and ventilatory goals rather than a standard "one-size-fits-all" treatment seems a promising new therapeutic strategy. In this article, we present a new physiology-guided treatment strategy to titrate the resuscitation efforts to patient's physiologic response after cardiac arrest. This approach can be applied during resuscitation attempts in highly monitored patients, such as those in the operating room or the intensive care unit, and could serve as a method for improving tissue perfusion and oxygenation while decreasing post-resuscitation adverse effects.

1. A10. Wollborn, J., Steiger, C., Doostkam, S., Schallner, N., Schroeter, N., Kari, F. A., Meinel, L., Buerkle, H., Schick, M. A., & Goebel, U. (2020). Carbon monoxide exerts functional neuroprotection after cardiac arrest using extracorporeal resuscitation in pigs. Critical Care Medicine, 48(4), E299–E307. https://doi.org/10.1097/CCM.0000000000004242

Objectives: Neurologic damage following cardiac arrest remains a major burden for modern resuscitation medicine. Cardiopulmonary resuscitation with extracorporeal circulatory support holds the potential to reduce morbidity and mortality. Furthermore, the endogenous gasotransmitter carbon monoxide attracts attention in reducing cerebral injury. We hypothesize that extracorporeal resuscitation with additional carbon monoxide application reduces neurologic damage.

Design: Randomized, controlled animal study.

Setting: University research laboratory.

Subjects: Landrace-hybrid pigs.

Interventions: In a porcine model, carbon monoxide was added using a novel extracorporeal releasing system after resuscitation from cardiac arrest.

Measurements and Main Results: As markers of cerebral function, neuromonitoring modalities (somatosensory-evoked potentials, cerebral oximetry, and transcranial Doppler ultrasound) were used. Histopathologic damage and molecular markers (caspase-3 activity and heme oxygenase-1 expression) were analyzed. Cerebral oximetry showed fast rise in regional oxygen saturation after carbon monoxide treatment at 0.5 hours compared with extracorporeal resuscitation alone (regional cerebral oxygen saturation, 73% ± 3% vs 52% ± 8%; $p < 0.05$). Median nerve somatosensory-evoked potentials showed improved activity upon carbon monoxide treatment, whereas post–cardiac arrest cerebral perfusion differences were diminished. Histopathologic damage scores were reduced compared with customary resuscitation strategies (hippocampus: sham, 0.4 ± 0.2; cardiopulmonary resuscitation, 1.7 ± 0.4; extracorporeal cardiopulmonary resuscitation, 2.3 ± 0.2; extracorporeal cardiopulmonary resuscitation with carbon monoxide application [CO-E-CPR], 0.9 ± 0.3; $p < 0.05$). Furthermore, ionized calcium-binding adaptor molecule 1 staining revealed reduced damage patterns upon carbon monoxide treatment. Caspase-3 activity (cardiopulmonary resuscitation, 426 ± 169 pg/mL; extracorporeal cardiopulmonary resuscitation, 240 ± 61 pg/mL; CO-E-CPR, 89 ± 26 pg/mL; $p < 0.05$) and heme oxygenase-1 (sham, 1 ± 0.1; cardiopulmonary resuscitation, 2.5 ± 0.4; extracorporeal cardiopulmonary resuscitation, 2.4 ± 0.2; CO-E-CPR, 1.4 ± 0.2; $p < 0.05$) expression were reduced after carbon monoxide exposure.

Conclusions: Carbon monoxide application during extracorporeal resuscitation reduces injury patterns in neuromonitoring and decreases histopathologic cerebral damage by reducing apoptosis. This may lay the basis for further clinical translation of this highly salutary substance.

1. A11. Hu, Y., Sun, D., Li, Y., Wang, X., Jiang, W., Shi, H., & Cui, D. (2020). Increased PINK1/Parkin-mediated mitophagy explains the improved brain protective effects of slow rewarming following hypothermia after cardiac arrest in rats. Experimental Neurology, 330. https://doi.org/10.1016/j.expneurol.2020.113326

Cerebral ischemia-reperfusion (I/R) after cardiac arrest (CA) induces mitochondrial dysfunction, and the timely removal of damaged mitochondria by mitophagy is reported to protect against cerebral I/R injury. Therapeutic hypothermia (TH) has become an important component of postresuscitation care for patients who return to spontaneous circulation after CA. Previous studies have shown that TH can activate mitophagy and can contribute a protective effect; however, the optimal rewarming rate and underlying mechanism of rewarming following TH remain largely unexplained. Here, we investigated the effects of different rewarming rates and whether mitophagy is involved in rewarming. After 5 min of asphyxial CA following 4 h of cooling, Sprague-Dawley rats were randomized into the normothermia, hypothermia, slow rewarming (0.5 °C/h) and fast rewarming (4 °C/h) groups. The hypothermia group was kept cool until tissue harvest, the rewarming duration for the slow

rewarming group and fast rewarming group was 6 h and 45 min, respectively. We found that slowly rewarmed rats had better survival at 72 h than normothermic rats and fast-rewarmed rats (70%, 25.71%, and 50%, respectively) and higher neurological deficit scores (NDSs), in which the medians were 57.33, 26, and 28.83, respectively. In addition, we explored the underlying mechanism during this process and found that PINK1/Parkin-mediated mitophagy was activated during hypothermia in the slow rewarming group but was inhibited in the fast rewarming group. Further inhibition of mitophagy in the slowly rewarmed rats resulted in severe apoptosis and decreased the mean NDS from 58.39 to 33.11, indicating the protective role of mitophagy. Moreover, the fast rewarming group exhibited deficiencies in PINK1 expression and mitophagy activity and marked accumulation of reactive oxygen species (ROS). Overall, our results highlighted a neuroprotective role of PINK1/Parkin-mediated mitophagy during slow rewarming after hypothermia.

1. A12. Suzuki, M., Hatakeyama, T., Nakamura, R., Saiki, T., Kamisasanuki, T., Sugiki, D., & Matsushima, H. (2020). Serum Magnesium Levels and Neurological Outcomes in Patients Undergoing Targeted Temperature Management After Cardiac Arrest. JEN: Journal of Emergency Nursing, 46(1), 59–65. https://doi.org/10.1016/j.jen.2019.10.006

Introduction: Magnesium plays a neuroprotective role at the physiologic level, but its neuroprotective role in patients undergoing targeted temperature management for cardiac arrest is not well established. We performed multiple logistic regression analysis to evaluate whether magnesium levels can predict neurological outcomes in patients undergoing targeted temperature management after cardiac arrest.

Methods: We retrospectively investigated data on 86 patients who had undergone targeted temperature management after cardiac arrest between December 2015 and November 2017. The primary outcome was to determine whether magnesium levels predict unfavorable neurological outcomes for patients with return of spontaneous circulation after targeted temperature management. Cerebral Performance Category 3, 4, or 5 indicated unfavorable neurological outcomes. We performed multiple logistic regression to evaluate the primary outcome, adjusting for the time to return of spontaneous circulation, motor score of the Glasgow Coma Scale, first-recorded cardiac rhythm, pH, and magnesium levels.

Results: Of the 86 patients, 58 had unfavorable neurological outcomes. The mean hospital stay was 19 days. Multivariable analysis indicated that magnesium levels were not associated with an unfavorable neurological outcome. In contrast, a time to return of spontaneous circulation greater than 30 minutes and Glasgow Coma Scale motor score of 1 were significantly associated with an unfavorable neurological outcome.

Discussion: Magnesium levels were not associated with an unfavorable neurological outcome according to multivariable analysis. We found that a time to return of spontaneous circulation greater than 30 minutes and Glasgow Coma Scale motor score of 1 might predict an unfavorable neurological outcome.

1. A13. Kuklin, V., Akhatov, N., Kondratiev, T., Konkayev, A., Baigenzhin, A., Konkayeva, M., Karibekov, T., Barlow, N., Tveita, T., & Dahl, V. (2019). The influences of morphine or ketamine pre-treatment on hemodynamic, acid-base status, biochemical markers of brain damage and early survival in rats after asphyxial cardiac arrest. BMC Anesthesiology, 19(1), N.PAG-N.PAG. https://doi.org/10.1186/s12871-019-0884-6

Background: In different models of hypoxia, blockade of opioid or N-methyl-D-aspartate (NMDA) receptors shows cardio- and neuroprotective effects with a consequent increase in animal survival. The aim of the study was to investigate effects of pre-treatment with Morphine or Ketamine on hemodynamic, acid-base status, early survival, and biochemical markers of brain damage in a rat model of asphyxial cardiac arrest (ACA).

Methods: Under anaesthesia with Thiopental Sodium 60 mg/kg, i.p., Wistar rats (n = 42) were tracheostomized and catheters were inserted in a femoral vein and artery. After randomization, the rats were pre-treated with: Morphine 5 mg/kg i.v. (n = 14); Ketamine 40 mg/kg i.v. (n = 14); or equal volume of i.v. NaCl 0.9% as a Control (n = 14). ACA was induced by corking of the tracheal tube for 8 min, and defined as a mean arterial pressure (MAP) < 20 mmHg. Resuscitation was started at 5 min after cardiac arrest (CA). Invasive MAP was recorded during experiments. Arterial pH and blood gases were sampled at baseline (BL) and 10 min after CA. At the end of experiments, all surviving rats were euthanised, brain and blood samples for measurement of Neuron Specific Enolase (NSE), s100 calcium binding protein B (s100B) and Caspase-3 (CS-3) were retrieved.

Results: At BL no differences between groups were found in hemodynamic or acid-base status. After 3 min of asphyxia, all animals had cardiac arrest (CA). Return of spontaneous circulation (MAP > 60 mmHg) was achieved in all animals within 3 min after CA. At the end of the experiment, the Ketamine pre-treated group had increased survival (13 of 14; 93%) compared to the Control (7 of 14; 50%) and Morphine (10 of 14; 72%) groups (p = 0.035). Biochemical analysis of plasma concentration of NSE and s100B as well as an analysis of CS-3 levels in the brain tissue did not reveal any differences between the study groups.

Conclusion: In rats after ACA, pre-treatment with Morphine or Ketamine did not have any significant influence on hemodynamic and biochemical markers of brain damage. However, significantly better pH level and increased early survival were found in the Ketamine pre-treated group.

1. A14. Gong, B., Dong, Y., He, C., Jiang, W., Shan, Y., Zhou, B. Y., & Li, W. (2019). Intravenous Transplants of Human Adipose-Derived Stem Cell Protect the Rat Brain From Ischemia-Induced Damage. Journal of Stroke &

Cerebrovascular Diseases, 28(3), 595–603. https://doi.org/10.1016/j.jstrokecerebrovasdis.2018.10.037

Background: Survival following cardiac arrest (CA) and subsequent cardiopulmonary resuscitation (CPR), to a great extent, depends on brain damage. Adipose-derived stem cells (ADSCs), as a source of paracrine growth factors and the capacity of neural differentiation may reduce this brain damage.

Objective: The purpose of this study is to evaluate the protection of ADSCs to brain damage following CPR.

Methods: Rats were divided into 3 groups, sham, CA, and ADSCs group. Rats in sham group went through sham surgery. Rats in CA group went through CA, CPR, and injection PBS (phosphate buffer saline). Rats in ADSCs group went through CA, CPR, and intravenous injection of ADSCs. Rats in sham group were sacrificed immediately after operation. At 24, 72, and 168 hours after return of spontaneous circulation operation, rats in CA and ADSCs group were randomly selected and sacrificed. Brain damage was evaluated by using Neurological Deficit Scale (NDS) score, hippocampal pathology, serum level of S100β, and apoptosis ratio of hippocampal neurons. Protein of brain derived neurotrophic factor (BDNF) and IL-6 (interleukin-6) in the hippocampus were detected.

Results: Compared with sham group, CA and ADSCs group showed a decrease in NDS score, an increased apoptosis ratio of hippocampal nerve cells, increased serum level of S100-β, and a significant increase in neuroprotective IL-6 and BDNF. In comparison to CA group, ADSCs group had a mild degree of brain damage and higher expression of IL-6 and BDNF.

Conclusions: In the acute stage of cerebral injury following CA, ADSCs might improve the prognosis of brain damage by stimulating the expression of neuroprotective IL-6 and BDNF.

1. A15. Early Goal-Directed Haemodynamic Optimization of Cerebral Oxygenation in Comatose Survivors After Cardiac Arrest: The Neuroprotect Post-Cardiac Arrest Trial - PubMed. (n.d.). Retrieved May 28, 2020, from https://pubmed.ncbi.nlm.nih.gov/30895296/?from_term=Early+goal-directed+haemodynamic+optimization+of+cerebral+oxygenation+in+comatose+survivors+after+cardiac+arrest%3A+the+Neuroprotect+post-cardiac+arrest+trial&from_pos=1

Aims: During the first 6-12 h of intensive care unit (ICU) stay, post-cardiac arrest (CA) patients treated with a mean arterial pressure (MAP) 65 mmHg target experience a drop of the cerebral oxygenation that may cause additional cerebral damage. Therefore, we investigated whether an early goal directed haemodynamic optimization strategy (EGDHO) (MAP 85-100 mmHg, SVO2 65-75%) is safe and could improve cerebral oxygenation, reduce anoxic brain damage, and improve outcome when compared with a MAP 65 mmHg strategy.

Methods and results: A total of 112 out-of-hospital CA patients were randomly assigned to EGDHO or MAP 65 mmHg strategies during the first 36 h of ICU stay. The primary outcome was the extent of anoxic brain damage as quantified by the percentage of voxels below an apparent diffusion coefficient (ADC) score of 650.10-6 mm2/s on diffusion weighted magnetic resonance imaging (at day 5 ± 2 post-CA). Main secondary outcome was favourable neurological outcome (CPC score 1-2) at 180 days. In patients assigned to EGDHO, MAP (P < 0.001), and cerebral oxygenation during the first 12 h of ICU stay (P = 0.04) were higher. However, the percentage of voxels below an ADC score of 650.10-6 mm2/s did not differ between both groups [16% vs. 12%, odds ratio 1.37, 95% confidence interval (CI) 0.95-0.98; P = 0.09]. Also, the number of patients with favourable neurological outcome at 180 days was similar (40% vs. 38%, odds ratio 0.98, 95% CI 0.41-2.33; P = 0.96). The number of serious adverse events was lower in patients assigned to EGDHO (P = 0.02).

Conclusion: Targeting a higher MAP in post-CA patients was safe and improved cerebral oxygenation but did not improve the extent of anoxic brain damage or neurological outcome.

1. A16. Maze, M., & Laitio, T. (2020). Neuroprotective Properties of Xenon. Molecular Neurobiology, 57(1), 118–124. https://doi.org/10.1007/s12035-019-01761-z

Xenon is a rare noble gas that was introduced into clinical practice more than 70 years ago. Xenon's clinical properties are predicated by its ability to fit into preformed cavities of macromolecules thereby altering their biological functions. One such action targets the NMDA-subtype of the glutamate receptors thereby inhibiting its excitatory action. As the glutamate receptors are pivotal for both anesthesia and acute neurological injury, its clinical use has included both general anesthesia as well as neuroprotection. In this manuscript, the efficacy and safety of xenon in clinical trials that address both the anesthetic and neuroprotective applications are discussed. Because of the clinical safety of this chemically inert monatomic gas, the lack of an alternative for neuroprotection, and encouraging phase 2 trial data, a multinational pivotal randomized clinical trial (XePOHCAS) has been launched to assess the utility of xenon for patients that have been successfully resuscitated following an out of hospital cardiac arrest but still remain comatose, indicating ongoing neurological ischemic-perfusion injury. If successful, the trial will herald a new era of treatments for previously intractable conditions such as traumatic brain injury, ischemic and hemorrhagic strokes, and anesthetic-induced developmental neurotoxicity.

1. A17. Active Temperature Management After Cardiac Surgery and Its Effect on Postoperative Cognitive Dysfunction - Full Text View - ClinicalTrials.gov. (2020, March 26). ClinicalTrials.Gov. https://clinicaltrials.gov/ct2/show/NCT03947671

This study will assess the effect of active postoperative temperature management and its effect on the cognitive function in

patients following coronary artery bypass graft (CABG) surgery to determine if active postoperative temperature management to maintain normothermia reduces postoperative cognitive dysfunction (POCD) in this population. Additionally, the investigators will explore differences in temperature control variability by using temperature management wraps combined with acetaminophen vs. acetaminophen alone in a pilot arm.

1. A18. Meurer, W. J. (2020, April 14). Influence of Cooling Duration on Efficacy in Cardiac Arrest Patients - Full Text View - ClinicalTrials.gov. ClinicalTrials.Gov. https://clinicaltrials.gov/ct2/show/NCT04217551

A multicenter, randomized, adaptive allocation clinical trial to determine if increasing durations of induced hypothermia are associated with an increasing rate of good neurological outcomes and to identify the optimal duration of induced hypothermia for neuroprotection in comatose survivors of cardiac arrest.

1. A19. Endre, Z. (2019). Long term survival of successfully resuscitated patients is primarily determined by their post-cardiac arrest neurological function. Orvosi Hetilap, 160(46), 1840–1844. https://doi.org/10.1556/650.2019.31592

[article in **Hungarian**]

Long term survival of successfully resuscitated patients is primarily determined by their post-cardiac arrest neurological function. If the patient undergoes a long-term resuscitation or remains comatose as part of the post-cardiac arrest syndrome (PCAS), organ-specific intensive care is urged to aim hemodynamic stabilisation, normalisation of organ perfusion and prevention of injuries at cellular level. One of the basic measures of PCAS intensive care is to prevent hypoxic brain injury by mild therapeutic hypothermia (THT). The physiological changes of the human body at hypothermic conditions require high level monitoring and specially focused intensive care limiting its implementation. The multicentric, controlled, randomized targeted temperature management (TTM) trial published in 2013 compared the TTM against the THT in the treatment of PCAS patients. The equal outcome of the 2 methods has partly changed the practice of the intensivists in the treatment of such patients. This manuscript gives the pros and cons for each therapeutic method in post-resuscitation therapy. Nevertheless, the author shows the possible implementations and the DRG (diagnosis-related group) reimbursement of the method in Hungary.

1. A20. Uysal, S., Lin, H. M., Trinh, M., Park, C. H., & Reich, D. L. (2020). Optimizing cerebral oxygenation in cardiac surgery: A randomized controlled trial examining neurocognitive and perioperative outcomes. Journal of Thoracic and Cardiovascular Surgery, 159(3), 943-953.e3. https://doi.org/10.1016/j.jtcvs.2019.03.036

Objective: The study objective was to determine whether targeted therapy to optimize cerebral oxygenation is associated with improved neurocognitive and perioperative outcomes.

Methods: In a prospective trial, intraoperative cerebral oximetry monitoring using bilateral forehead probes was performed in cardiac surgical patients who were randomly assigned to an intervention group in which episodes of cerebral oxygen desaturation (<60% for >60 consecutive seconds at either probe) triggered an intervention protocol or a control group in which the cerebral oximetry data were hidden from the clinical team, and no intervention protocol was applied. Cognitive testing was performed preoperatively and at postoperative months 3 and 6; domains studied were response speed, processing speed, attention, and memory. Perioperative outcomes studied were death, hospital length of stay, intensive care unit length of stay, postoperative day of extubation, time on mechanical ventilation, intensive care unit delirium, Sequential Organ Failure Assessment on intensive care unit admission, and intensive care unit blood transfusion.

Results: Group mean memory change scores were significantly better in the intervention group at 6 months (0.60 [standard error, 0.30] vs −0.17 [standard error, 0.33], adjusted P = .008). However, presence, duration, and severity of cerebral desaturation were not associated with cognitive change scores. Perioperative outcomes did not differ between the intervention and control groups.

Conclusions: Targeted therapy to optimize cerebral oxygenation was associated with better memory outcome in a group of cardiac surgical patients. Some aspects of the protocol other than desaturation duration and severity contributed to the observed neuroprotective effect.

1. A21. Zheng, J.-H., Xie, L., Li, N., Fu, Z.-Y., Tan, X.-F., Tao, R., Qin, T., & Chen, M.-H. (2019). PD98059 protects the brain against mitochondrial-mediated apoptosis and autophagy in a cardiac arrest rat model. Life Sciences, 232, 116618. https://doi.org/10.1016/j.lfs.2019.116618

Aims: Mitochondrial dysfunction has been regarded as one of the hallmarks of cerebral ischemia-reperfusion injury. In previous studies, we have provided evidence that the extracellular signaling pathway (ERK) 1/2 inhibitor PD98059 improved the neurological deficits by modulating antioxidant and anti-apoptotic activities in rats subjected to cardiac arrest/cardiopulmonary resuscitation (CA/CPR). Since oxidative stress can activate mitochondria-dependent apoptosis and autophagy, we further explored the effects of PD98059 on mitochondria involved with apoptosis and autophagy in rat CA model.

Materials and methods: We disposed PD98059 in CA/CPR rats, tested the mitochondrial-mediated apoptosis pathway in brain

tissues at 24 h post-resuscitation by mitochondrial permeability transition pores (MPTP), cytochrome c (CytC), BCL-2, BAX, caspase-3, as well as autophagy by LC3, Beclin-1, and p62. Furthermore, we explored the relationship of dynamin-related protein 1 (Drp1) with apoptosis and autophagy.

Key findings: Our study showed that PD98059 decreased the openings of MPTP, CytC release, caspase3 activation, apoptotic indices, LC3-II, Beclin-1and increased P62. PD98059 also inhibited mitochondria-dependent apoptosis and the activity of autophagy in a dose-dependent manner in rat cerebral cortices at 24 h post-resuscitation. The generation of phosphorylated Drp1-616 was down-regulated accompanied by a decrease of TUNEL-positive cells and LC3 in dual immunostaining after PD98059 inhibited activation of ERK signaling pathway in a dose-dependent manner in rat cerebral cortices at 24 h post-resuscitation.

Significance: PD98059 protects the brain against mitochondrial-mediated apoptosis and autophagy at 24 h post-resuscitation in rats subjected to CA/CPR, which is linked with the downregulation of Drp1 expression.

1. A22. Yong, Y., Guo, J., Zheng, D., Li, Y., Chen, W., Wang, J., Chen, W., Wang, K., & Wang, Y. (2019). Electroacupuncture pretreatment attenuates brain injury in a mouse model of cardiac arrest and cardiopulmonary resuscitation via the AKT/eNOS pathway. Life Sciences, 235, 116821. https://doi.org/10.1016/j.lfs.2019.116821

Aims: This study aims to examine the effects of electroacupuncture (EA) pretreatment on brain injury after cardiac arrest and cardiopulmonary resuscitation (CA/CPR) and its underlying mechanisms.

Materials and methods: Adult male C57BL/6 mice were subjected to 6 min of cardiac arrest induced with a potassium chloride infusion and resuscitated by chest compressions and an epinephrine infusion. During the 3 days prior to CA/CRP, mice received EA pretreatment (1 mA, 2 Hz; daily session of 30 min) at the Baihui acupoint (GV20) once daily. Stimulation at a nonacupoint served as a control. In mechanistic studies, mice received the AKT inhibitor LY294002 or endothelial nitric oxide synthase (eNOS) inhibitor L-NIO 30 min before EA pretreatment. A neurological assessment was conducted 24 h after CA/CRP, followed by animal sacrifice and evaluation of physiological brain damage.

Key findings: CA/CPR resulted in severe brain injury as evidenced by neurological deficits and increased neuronal apoptosis, oxidative stress and the proinflammatory cytokines TNF-α and IL-6. EA pretreatment at the GV20 acupoint but not at a nonacupoint attenuated the neurological deficits and the pathological changes induced by CA/CPR. LY294002 or L-NIO eliminated the neuroprotective effects of the EA pretreatment.

Significance: This study showed that EA pretreatment at the GV20 acupoint can protect the brain from damage associated with globalized ischemia followed by reperfusion and that these protective effects occur via the AKT/eNOS signaling pathway.

1. A23. Lee, R. H.-C., Couto E Silva, A., Possoit, H. E., Lerner, F. M., Chen, P.-Y., Azizbayeva, R., Citadin, C. T., Wu, C. Y.-C., Neumann, J. T., & Lin, H. W. (2019). Palmitic acid methyl ester is a novel neuroprotective agent against cardiac arrest. Prostaglandins, Leukotrienes, and Essential Fatty Acids, 147, 6–14. https://doi.org/10.1016/j.plefa.2018.11.011

We previously discovered that palmitic acid methyl ester (PAME) is a potent vasodilator first identified and released from the superior cervical ganglion and remain understudied. Thus, we investigated PAME's role in modulating cerebral blood flow (CBF) and neuroprotection after 6 min of cardiac arrest (model of global cerebral ischemia). Our results suggest that PAME can enhance CBF under normal physiological conditions, while administration of PAME (0.02 mg/kg) immediately after cardiopulmonary resuscitation can also enhance CBF in vivo. Additionally, functional learning and spatial memory assessments (via T-maze) 3 days after asphyxial cardiac arrest (ACA) suggest that PAME-treated rats have improved learning and memory recovery versus ACA alone. Furthermore, improved neuronal survival in the CA1 region of the hippocampus were observed in PAME-treated, ACA-induced rats. Altogether, our findings suggest that PAME can enhance CBF, alleviate neuronal cell death, and promote functional outcomes in the presence of ACA.

1. A24. Hope Kilgannon, J., Hunter, B. R., Puskarich, M. A., Shea, L., Fuller, B. M., Jones, C., Donnino, M., Kline, J. A., Jones, A. E., Shapiro, N. I., Abella, B. S., Trzeciak, S., & Roberts, B. W. (2019). Partial pressure of arterial carbon dioxide after resuscitation from cardiac arrest and neurological outcome: A prospective multi-center protocol-directed cohort study. Resuscitation, 135, 212–220. https://doi.org/10.1016/j.resuscitation.2018.11.015

Aims: Partial pressure of arterial carbon dioxide ($PaCO_2$) is a regulator of cerebral blood flow after brain injury. We sought to test the association between $PaCO_2$ after resuscitation from cardiac arrest and neurological outcome.

Methods: A prospective protocol-directed cohort study across six hospitals. Inclusion criteria: age ≥18, non-traumatic cardiac arrest, mechanically ventilated after return of spontaneous circulation (ROSC), and receipt of targeted temperature management. Per protocol, $PaCO_2$ was measured by arterial blood gas analyses at one and six hours after ROSC. We determined the mean $PaCO_2$ over this initial six hours after ROSC. The primary outcome was good neurological function at hospital discharge, defined a priori as a modified Rankin Scale ≤3. Multivariable Poisson regression analysis was used to test the association between $PaCO_2$ and neurological outcome.

Results: Of the 280 patients included, the median (interquartile range) $PaCO_2$ was 44 (37–52) mmHg and 30% had good neurological function. We found mean $PaCO_2$ had a quadratic (inverted "U" shaped) association with good neurological

outcome, with a mean PaCO2 of 68 mmHg having the highest predictive probability of good neurological outcome, and worse neurological outcome at higher and lower PaCO2. Presence of metabolic acidosis attenuated the association between PaCO2 and good neurological outcome, with a PaCO2 of 51 mmHg having the highest predictive probability of good neurological outcome among patients with metabolic acidosis.

Conclusion: PaCO2 has a “U” shaped association with neurological outcome, with mild to moderate hypercapnia having the highest probability of good neurological outcome.

1. A25. Cho, J. H., Tae, H.-J., Kim, I.-S., Song, M., Kim, H., Lee, T.-K., Kim, Y.-M., Ryoo, S., Kim, D. W., Lee, C.-H., Hwang, I. K., Yan, B. C., Kang, I. J., Won, M.-H., & Lee, J.-C. (2019). Melatonin alleviates asphyxial cardiac arrest-induced cerebellar Purkinje cell death by attenuation of oxidative stress. Experimental Neurology, 320, 112983. https://doi.org/10.1016/j.expneurol.2019.112983

Although multiple reports using animal models have confirmed that melatonin appears to promote neuroprotective effects following ischemia/reperfusion-induced brain injury, the relationship between its protective effects and activation of autophagy in Purkinje cells following asphyxial cardiac arrest and cardiopulmonary resuscitation (CA/CPR) remains unclear. Rats used in this study were randomly assigned to 6 groups as follows; vehicle-treated sham operated group, vehicle-treated asphyxial CA/CPR operated group, melatonin-treated sham operated group, melatonin-treated asphyxial CA/CPR operated group, PDOT (a MT2 melatonin receptor antagonist) plus (+) melatonin-treated sham operated group and PDOT+melatonin-treated asphyxial CA/CPR operated group. Melatonin (20 mg/kg, i.p., 4 times before CA and 3 times after CA) treatment significantly improved survival rate and neurological deficit compared with the vehicle-treated asphyxial CA/CPR rats (survival rates ≥40% vs 10%), showing that melatonin treatment exhibited protective effect against asphyxial CA/CPR-induced Purkinje cell death. The protective effect of melatonin against CA/CPR-induced Purkinje cell death paralleled a remarkable attenuation of autophagy-like processes (Beclin-1, Atg7 and LC3), as well as a dramatic reduction in superoxide anion radical (O2·-), intense enhancements of CuZn superoxide dismutase (SOD1) and MnSOD (SOD2) expressions. Furthermore, the protective effect was notably reversed by treatment with PDOT, which is a selective MT2 antagonist. In brief, melatonin conferred neuroprotection against asphyxial CA/CPR-induced Purkinje cell death via inhibiting autophagic activation by reducing expressions of O2·- and increasing expressions of antioxidant enzymes, and suggests that MT2 is involved in neuroprotective effect of melatonin against Purkinje cell death caused by asphyxial CA/CPR.

1. A26. Quillinan, N., Dingman, A. L., Deng, G., Tatum, S., Orfila, J. E., Clevenger, A. C., Klawitter, J., Traystman, R. J., & Herson, P. S. (2019). Single dose of 17β-estradiol provides transient neuroprotection in female juvenile mice after cardiac-arrest and cardiopulmonary resuscitation. Neurochemistry International, 127, 80–86. https://doi.org/10.1016/j.neuint.2018.11.013

Each year there are approximately 7000 out of hospital cardiac arrests in the pediatric population, with 30% resuscitation rate and a 6–10% rate of survival to hospital discharge. Survivors of cardiac arrest exhibit learning and memory deficits that are devastating during the school years. Delayed neuronal cell death occurs in the hippocampus following cardiac arrest and likely contributes to memory impairments. Circulating endogenous estrogen in young adult females has been shown to provide protection against ischemic cell death, as does chronic exogenous administration of 17β-estradiol (E2). Chronic estrogen benefit can have undesirable feminizing effects, particularly in pre-adolescents. Here, we tested if a single-dose of E2 is neuroprotective in our pediatric cardiac arrest mouse model performed in juvenile mice. We subjected P21P25 C57Blk6 male and female mice to 8 min of cardiac arrest followed by cardiopulmonary resuscitation (CA/CPR). This developmental stage preceded the hormonal onset and serum estradiol and testosterone levels were not different in males and females. A single dose of E2 (100μg/kg) or vehicle was administered 30 min after resuscitation. Neuronal cell death measured 3 days after CA/CPR showed reduced hippocampal cell death in E2-treated females, but not males. Benefit of E2 in females was blocked by the P38 MAPK inhibitor, SB203580. Hippocampal-dependent memory function was equally impaired in E2-and vehicle-treated females measured in the contextual fear conditioning task at 7 days. Our findings demonstrate female-specific transient neuroprotection with E2 that does not provide sustained functional benefit.

1. A27. Han, R., Zhang, G., Qiao, X., Guo, Y., Sun, L., Li, J., Gao, C., & Sun, X. (2020). α7 Nicotinic Acetylcholine Receptor Mediates the Neuroprotection of Remote Ischemic Postconditioning in a Rat Model of Asphyxial Cardiac Arrest. The Journal of Surgical Research, 246, 6–18. https://doi.org/10.1016/j.jss.2019.07.091

Background: Remote ischemic postconditioning (RIPost) has been shown to reduce the ischemia–reperfusion injury of the heart and brain. However, the protection mechanisms have not yet been fully elucidated. We have observed that RIPost could alleviate the brain injury after cardiac arrest (CA). The aim of this study was to explore whether α7 nicotinic acetylcholine receptor (α7nAChR) mediates the neuroprotection of RIPost in a rat model of asphyxial CA.

Materials and methods: Asphyxial CA model was induced by occlusion of the tracheal tube for 8 min and resuscitated later. RIPost produced by three cycles of 15-min occlusion and 15-min release of the right hind limb by a tourniquet was performed respectively at the moment and the third hour after restoration of spontaneous circulation. The α7nAChR agonist PHA-543613 and the antagonist methyllycaconitine (MLA) were used to investigate the role of α7nAChR in mediating neuroprotective effects.

Results: Results showed that α7nAChR was decreased in hippocampus and cortex after resuscitation, whereas RIPost could attenuate the reduction. The use of PHA-543613 provided neuroprotective effects against cerebral injury after CA.

Furthermore, RIPost decreased the levels of neuron-specific enolase, inflammatory mediators, the number of apoptotic cells, and phosphorylation of nuclear factor-κB while increased the phosphorylation of signal transducer and activator of transcription-3. However, the above effects of RIPost were attenuated by α7nAChR antagonist methyllycaconitine.

Conclusions: Neuroprotection of RIPost was related with the activation of α7nAChR, which could suppress nuclear factor-κB and activate signal transducer and activator of transcription-3 in a rat asphyxial CA model.

1. A28. Węgrzyn, D., Kutwin-Chojnacka, A., Bilski, J., Mroszczyk, K., & Węgrzyn, K. (2019). Neurotrophic Factors in the Treatment of Acute Brain Hypoxia Secondary to Cardiac Arrest: a Case Report. Journal of Medicine and Life, 12(3), 233–235. https://doi.org/10.25122/jml-2019-1007

Finding neuroprotective agents to counteract the deleterious effects of hypoxia on neuronal cells successfully is one of the most critical targets of clinical research since preclinical studies have identified potential neuroprotective strategies. In clinical practice, amantadine and piracetam are used with reasonable success. We present the cases of three patients with acute brain hypoxia secondary to cardiac arrest, to whom Cerebrolysin was added to the standard neuroprotective treatment regimen, leading to a notable improvement in functional outcome.

Neuronal Injury Following Cardiac Arrest and Neuroprotection (B)

1. B1. Passi, N. N., Oliver, C. M., & Dhadwal, K. (2019). Hospital management of the post-cardiac arrest patient: priorities and challenges. British Journal of Hospital Medicine (17508460), 80(2), C22–C27. https://doi.org/10.12968/hmed.2019.80.2.C22

[no abstract provided]

1. B2. Sun, J., He, F., Gao, Y., Zhou, Y., Zhang, H., Huang, M., & Bi, H. (2020). Lipidomics-based study on the neuroprotective effect of geissoschizine methyl ether against oxidative stress-induced cytotoxicity. Journal of Ethnopharmacology, 253, N.PAG-N.PAG. https://doi.org/10.1016/j.jep.2020.112636

Ethnopharmacological relevance: Lipid homoeostasis is important for neurodevelopment, cell signaling and neurotransmission. Alteration of lipid metabolism has been demonstrated in many neurological disorders and neurodegenerative diseases. Geissoschizine methyl ether (GM) is an active alkaloid ingredient in the traditional Chinese medicine Uncaria hook. It has been shown that GM has strong potency in neuroprotective activity and GM reduces the production of reactive oxygen species by regulating glucose metabolism, which protects neurons against oxidative stress-induced cell death. However, it is unknown whether GM could regulate neuronal lipid metabolism during oxidative challenge.

Aim of the study: The current study aimed to explore whether GM regulates lipid metabolism in oxidative damaged neurons and to determine the underlying mechanism involved in this neuro-protection.

Materials and methods: Using a glutamate-induced oxidative toxicity model in mouse hippocampal neuronal cell line (HT-22 cells), we investigated the effect of GM on glutamate-induced lipid peroxidation, lipotoxicity and mitochondrial dysfunction. In order to clarify the mechanism underlying the neuroprotection by GM, lipid metabolomics was performed to investigate whether GM prevent oxidative stress-induced lipid metabolism disruption. Furthermore, the expression of lipid metabolism-related genes was measured.

Results: The results show the protective effect of GM against oxidative stress through blocking glutamate-induced lipid peroxidation and lipotoxicity. Overall, lipidomics analysis revealed that glutamate treatment resulted in different extents of changes in a wide range of lipid classes such as fatty acids (FA), triacylglycerol (TG), sphingomyelin (SM), cardiolipin (CL), lysophosphatidylcholines (LPC). However, GM treatment can significantly reverse glutamate-induced lipids disorder to the homeostasis level. GM prevented the disruption of lipid metabolism by regulating the expression of lipid homeostasis related genes, which contributes to preserve mitochondrial function under oxidative damage.

Conclusion: These findings clearly demonstrated a novel protective mechanism of GM against glutamate-induced oxidative toxicity in neurons via regulating lipid metabolism. GM may provide an effective approach for the prevention and treatment of oxidative damaged neurons.

1. B3. Jang, M.-S., Oh, S. K., Lee, S. W., Jeong, S.-H., & Kim, H. (2019). Moderate brain hypothermia started before resuscitation improves survival and neurobehavioral outcomes after CA/CPR in mice. The American Journal of Emergency Medicine, 37(10), 1942–1948. https://doi.org/10.1016/j.ajem.2019.01.027

Aim of the study: No definitive experimental or clinical evidence exists whether brain hypothermia before, rather than during or after, resuscitation can reduce hypoxic-ischemic brain injury following cardiac arrest/cardiopulmonary resuscitation (CA/CPR) and improve outcomes. We examined the effects of moderate brain hypothermia before resuscitation on survival and histopathological and neurobehavioral outcomes in a mouse model.

Methods: Adult C57BL/6 male mice (age: 8–12 weeks) were subjected to 8-min CA followed by CPR. The animals were

randomly divided into sham, normothermia (NT; brain temperature 37.5 °C), and extracranial hypothermia (HT; brain temperature 28–32 °C) groups. The hippocampal CA1 was assessed 7 day after resuscitation by histochemical staining. Neurobehavioral outcomes were evaluated by the Barnes maze (BMT), openfield (OFT), rotarod, and light/dark (LDT) tests. Cleaved caspase-3 and heat shock protein 60 (HSP70) levels were investigated by western blotting.

Results: The HT group exhibited higher survival and lower CA1 neuronal injury than did the NT group. HT mice showed improved spatial memory in the BMT compared with NT mice. NT mice travelled a shorter distance in the OFT and tended to spend more time in the light compartment in the LDT than did sham and HT mice. The levels of cleaved caspase-3 and HSP70 were non-significantly higher in the NT than in the sham and HT groups.

Conclusions: Moderate brain hypothermia before resuscitation improved survival and reduced histological neuronal injury, spatial memory impairment, and anxiety-like behaviours after CA/CPR in mice.

1. B4. Yuan, J., Yang, M.-C., Wu, M.-J., & Gou, Y.-S. (2019). Sedative depth on neurological outcomes in a juvenile rat model of cardiopulmonary resuscitation. Medical Hypotheses, 132, 109233. https://doi.org/10.1016/j.mehy.2019.109233

The guidelines for cardiopulmonary resuscitation (CPR) in pediatric advanced life support suggest that midazolam is the preferred agent for sedation in patients with mild hypothermia, whereas children with cardiac arrest (CA) are at a crucial stage regarding their immature nervous system. Studies have shown that midazolam may have a detrimental effect on the developmental of the pediatric nervous system. Our previous study found that midazolam induced neuronal damage after CPR in young rats. It is speculated that: midazolam causes the potential injury of neurons by inhibiting mitochondrial autophagy expression and is an important factor for the poor prognosis in children after successful CPR. This project intends to adopt the modified asphyxiant CPR model in juvenile rats. Survival rate, neurological function and histopathological changes were evaluated to determine the protective effects of appropriate sedation depth on cerebral ischemia-reperfusion injury in juvenile rats after CPR. Combined with cell biology and molecular biology related technologies, the mechanism by which the mitochondrial pinkl-parkin signaling pathway induces autophagy to inhibit neuronal apoptosis may be key factor in the protective effects of sedation depth on the brain. The aim of this study is to provide experimental evidence and elucidate the mechanisms of improvement of cerebral ischemia-reperfusion injury by sedation depth in children after successful CPR and to lay a theoretical and experimental basis for clinical treatment.

Ketamine and Neuroprotection (C)

1. C1. Pribish, A., Wood, N., & Kalava, A. (2020). A Review of Nonanesthetic Uses of Ketamine. Anesthesiology Research and Practice, 2020, 1–15. https://doi.org/10.1155/2020/5798285

Ketamine, a nonselective NMDA receptor antagonist, is used widely in medicine as an anesthetic agent. However, ketamine's mechanisms of action lead to widespread physiological effects, some of which are now coming to the forefront of research for the treatment of diverse medical disorders. This paper aims at reviewing recent data on key nonanesthetic uses of ketamine in the current literature. MEDLINE, CINAHL, and Google Scholar databases were queried to find articles related to ketamine in the treatment of depression, pain syndromes including acute pain, chronic pain, and headache, neurologic applications including neuroprotection and seizures, and alcohol and substance use disorders. It can be concluded that ketamine has a potential role in the treatment of all of these conditions. However, research in this area is still in its early stages, and larger studies are required to evaluate ketamine's efficacy for nonanesthetic purposes in the general population.

1. C2. Kuklin, V., Akhatov, N., Kondratiev, T., Konkayev, A., Baigenzhin, A., Konkayeva, M., Karibekov, T., Barlow, N., Tveita, T., & Dahl, V. (2019). The influences of morphine or ketamine pre-treatment on hemodynamic, acid-base status, biochemical markers of brain damage and early survival in rats after asphyxial cardiac arrest. BMC Anesthesiology, 19(1), N.PAG-N.PAG. https://doi.org/10.1186/s12871-019-0884-6

Background: In different models of hypoxia, blockade of opioid or N-methyl-D-aspartate (NMDA) receptors shows cardio- and neuroprotective effects with a consequent increase in animal survival. The aim of the study was to investigate effects of pre-treatment with Morphine or Ketamine on hemodynamic, acid-base status, early survival, and biochemical markers of brain damage in a rat model of asphyxial cardiac arrest (ACA).

Methods: Under anaesthesia with Thiopental Sodium 60 mg/kg, i.p., Wistar rats (n = 42) were tracheostomized and catheters were inserted in a femoral vein and artery. After randomization, the rats were pre-treated with: Morphine 5 mg/kg i.v. (n = 14); Ketamine 40 mg/kg i.v. (n = 14); or equal volume of i.v. NaCl 0.9% as a Control (n = 14). ACA was induced by corking of the tracheal tube for 8 min, and defined as a mean arterial pressure (MAP) < 20 mmHg. Resuscitation was started at 5 min after cardiac arrest (CA). Invasive MAP was recorded during experiments. Arterial pH and blood gases were sampled at baseline (BL) and 10 min after CA. At the end of experiments, all surviving rats were euthanised, brain and blood samples for measurement of Neuron Specific Enolase (NSE), s100 calcium binding protein B (s100B) and Caspase-3 (CS-3) were retrieved.

Results: At BL no differences between groups were found in hemodynamic or acid-base status. After 3 min of asphyxia, all

animals had cardiac arrest (CA). Return of spontaneous circulation (MAP > 60 mmHg) was achieved in all animals within 3 min after CA. At the end of the experiment, the Ketamine pre-treated group had increased survival (13 of 14; 93%) compared to the Control (7 of 14; 50%) and Morphine (10 of 14; 72%) groups (p = 0.035). Biochemical analysis of plasma concentration of NSE and s100B as well as an analysis of CS-3 levels in the brain tissue did not reveal any differences between the study groups.

Conclusion: In rats after ACA, pre-treatment with Morphine or Ketamine did not have any significant influence on hemodynamic and biochemical markers of brain damage. However, significantly better pH level and increased early survival were found in the Ketamine pre-treated group.

1. C3. Wang, R., Zhang, Z., Kumar, M., Xu, G., & Zhang, M. (2019). Neuroprotective potential of ketamine prevents developing brain structure impairment and alteration of neurocognitive function induced via isoflurane through the PI3K/AKT/GSK-3β pathway. Drug Design, Development and Therapy, 13, 501–512. https://doi.org/10.2147/DDDT.S188636

Background: The aim of the current experimental study was to scrutinize the neuroprotective effect of ketamine on the isoflurane (iso)-induced cognitive dysfunction in rats via phosphoinositide 3 kinase (PI3K)/protein kinase B (AKT)/glycogen synthase kinase 3β (GSK-3β) pathway.

Materials and methods: Sprague-Dawley rats were used for the current experimental study. The rats were divided into six groups and rats were treated with ketamine and memantine. For the estimation of cognitive function study, we used the Morris water test. Pro-inflammatory cytokines such as IL-1β, IL-6, tumor necrosis factor-α (TNF-α), and caspase-6; the antioxidant parameters malondialdehyde, glutathione, superoxide dismutase, catalase, and protein carbonyl; acetylcholinesterase, amyloid β, and brain-derived neurotrophic factor were estimated, respectively. The protein expression of AKT, GSK-3β, p21WAF1/CIP1, and p53 was also estimated, respectively.

Results: Ketamine significantly enhanced cognitive function and showed anti-inflammatory and antioxidant effects, and exhibited the neuroprotective effect of ketamine against the isoflurane-induced cognitive impairment. Additionally, ketamine significantly ($P<0.005$) suppressed IL-1β, TNF-α, IL-6, caspase-6 and p21WAF1/CIP1, p53 expression and up-regulated the PI3K/AKT/GSK-3β expression in the group of iso-induced rats.

Conclusion: We can conclude that ketamine prevented the cognitive impairment induced by isoflurane anesthesia through anti-apoptotic, anti-inflammatory, and antioxidant effects via the PI3K/AKT/GSK-3β pathway.

1. C4. Fujikawa, D. G. (2019). Starting ketamine for neuroprotection earlier than its current use as an anesthetic/antiepileptic drug late in refractory status epilepticus. Epilepsia, 60(3), 373–380. https://doi.org/10.1111/epi.14676

Ketamine is currently being used as an anesthetic/antiepileptic drug in refractory status epilepticus. To validate its use, 2 clinical trials are recruiting patients. However, preclinical studies of its use in chemically induced status epilepticus in rodents have shown that it is remarkably neuroprotective, through N -methyl-d -aspartate–receptor blockade, even when given after the onset of status epilepticus. Human studies have shown that status epilepticus–induced brain damage can be caused by a glutamate analogue and that it occurs in the same brain regions as in the animal studies. We therefore propose that ketamine be started early in the course of human status epilepticus as a neuroprotectant and that it be continued until epileptic discharges are eliminated. Using it as an anesthetic/antiepileptic drug late in the course of refractory status epilepticus only ensures that it is given after widespread brain damage has occurred.

1. C5. Kuklin, V. (2019, July 5). Morphine or/and Ketamine in Out-of-hospital Cardiac Arrest - Full Text View - ClinicalTrials.gov. ClinicalTrials.Gov. https://clinicaltrials.gov/ct2/show/NCT04009759

Preclinical studies demonstrate that opioids can preserve cellular integrity status during acute hypoxia in many organs and tissues including: intestine, skeletal muscle, myocardium and brain. Morphine has been shown to significantly increase the survival of mice and rats in acute hypoxia conditions. In the experimental model with rats exposed to hypoxic gas (5% oxygen, 95% N_2) for 70 min, all seven rats in the naloxone pre-treated group died at the end of the experiments while only one out of seven rats died in the Morphine (5 mg/kg) pretreated group, and five from the seven rats died in the control group. In the experiments where the rats were exposed to 8 min anoxia, pre-treatment with Morphine (5mg/kg), or Ketamine (40 mg/kg), resulted in higher survival in both groups as compared to the control group (data not yet published). No publications looking at the survival rate in animals with treatment by Morphine before cardiac arrest have been published yet. Meanwhile, two recent retrospective studies demonstrated that patients who were treated with opioids before or during cardiac arrest had a statistically significantly higher survival rate and much better neurological outcome compared to untreated patients. As it is not possible to apply cardiac arrest to animal without any anaesthesia (main limitation of all experimental models of cardiac arrest), the sympathomimetic effects and possible neuroprotective features of Ketamine should be tested in patients with cardiac arrest. Additional topic for possible clinical research of Ketamine as well as Morphine could be their analgesic effects as vigorous thoracic compression with possible trauma of the ribs may lead to severe pain and stress related negative body responses in patients surviving CPR.
